# HHIPL2 positively governs Hedgehog signaling to accelerate non-small cell lung cancer progression via enhancing HNRNPC-mediated HNF1A mRNA stabilization

**DOI:** 10.1038/s41419-025-08331-3

**Published:** 2025-12-18

**Authors:** Ning Mu, Fanrong Liu, Xiangqing Song, Biao Wang, Yilin Zhu, Lingbing Li, Lingxiao Yang, Huacong Sui, Jinfu Wang, Fengyuan Gao, Yongjia Zhou, Yanfeng Lv, Zhongxian Tian, Peichao Li, Xiaogang Zhao

**Affiliations:** 1 Department of Thoracic Surgery, The Second Hospital, Cheeloo College of Medicine, Shandong University, Jinan, China; 2 Key Laboratory of Precision Diagnosis and Treatment of Lung Tumors in Shandong Provincial Medicine and Health, Shandong University, Jinan, China; 3 Key Laboratory of Basic Research and Clinical Transformation of Thoracic Tumors in Shandong Provincial Colleges and Universities, Shandong University, Jinan, China; 4 Department of Colorectal and Anal Surgery, The Second Hospital, Cheeloo College of Medicine, Shandong University, Jinan, China

**Keywords:** Non-small-cell lung cancer, Non-small-cell lung cancer

## Abstract

Sonic Hedgehog signaling is aberrantly activated in non-small cell lung cancer (NSCLC). However, the regulatory molecules of Sonic Hedgehog signaling during NSCLC development are still largely unknown. Here, we demonstrated that HHIP Like 2 (HHIPL2) is a crucial Sonic Hedgehog signaling regulator, accelerating NSCLC progression via positively governing Sonic Hedgehog signaling. Clinically, HHIPL2 is highly expressed in NSCLC and indicates a poor prognosis. Consistently, via depletion or gain of HHIPL2 in vivo and in vitro, we identified its oncogenic role in NSCLC proliferation and metastasis. Mechanistically, HHIPL2 interacted with the RNA-binding protein HNRNPC to alter its nucleo-cytoplasmic translocation. HHIPL2-regulated HNRNPC accumulation in the cytoplasm promoted the mRNA stability of HNF1A, a transcription factor for SHH, which subsequently enhanced the Sonic Hedgehog signaling activity to facilitate NSCLC progression. Furthermore, we discovered that triptolide (TPL), an HNF1A inhibitor, impeded HHIPL2-mediated Sonic Hedgehog signaling activation and NSCLC malignancy. Therefore, our findings not only uncover a previously unrecognized role for HHIPL2 in regulating the Sonic Hedgehog signaling pathway but also highlight a novel HHIPL2/HNRNPC/HNF1A axis as an attractive target for NSCLC therapy.

**Figure:**
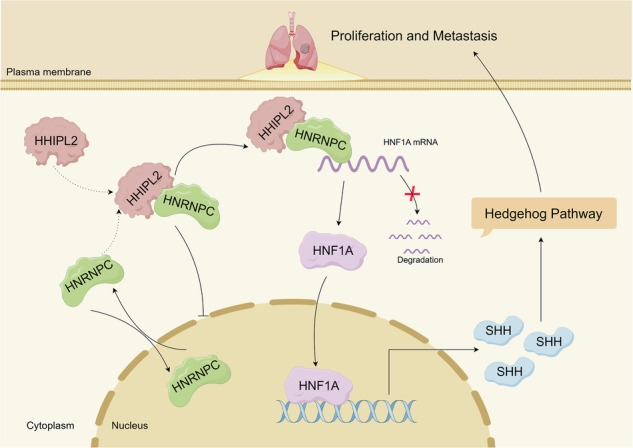
Schematic diagram (created by Figdraw.com) showing that HHIPL2 positively governs Hedgehog signaling to accelerate NSCLC progression via enhancing HNRNPC-mediated HNF1A mRNA stabilization.

Schematic diagram (created by Figdraw.com) showing that HHIPL2 positively governs Hedgehog signaling to accelerate NSCLC progression via enhancing HNRNPC-mediated HNF1A mRNA stabilization.

## Introduction

Lung cancer (LC) is one of the most common malignancies worldwide. Nearly 85% of LC cases are diagnosed with non-small cell lung cancer (NSCLC) [1–3]. Despite the significant progress in NSCLC therapy in the past few decades, high tumor aggressiveness and a lack of effective treatment are the main reasons for its terrible mortality rate, which remains a considerable challenge for human health [4, 5]. Therefore, identifying new molecular markers and achieving detailed molecular mechanisms are essential for developing new targeted therapy strategies and improving NSCLC prognostics.

The Hedgehog signaling pathway controls numerous biological processes throughout embryonic development and exerts critical regulatory functions [6]. Various studies indicate that uncontrolled activation of the Hedgehog pathway initiates or promotes a variety of cancers [7]. Hedgehog signaling has been reported to increase proliferation and tumorigenesis in NSCLC in vitro and in vivo [8–10]. In mammals, the Hedgehog signaling pathway is activated when Hedgehog ligands (Sonic Hedgehog [SHH], Desert Hedgehog [DHH], Indian Hedgehog [IHH]) bind to their repressive receptor Patched (PTCH1), a 12-transmembrane protein, leading to alleviation of its repression on the signaling transducer, Smoothened (SMO), a 7-transmembrane protein. PTCH1 is the receptor for the Hedgehog signaling pathway, whereas SMO is the activator of the downstream signaling. In the absence of Hedgehog ligands, PTCH1 suppresses the activity of SMO, which is released upon Hedgehog ligand binding to PTCH1, allowing SMO to activate its downstream targets [11, 12]. The unleashed SMO can then activate the GLI transcription factors, resulting in the expression of downstream target genes like GLI1 and PTCH1 [13].

Among all the Hedgehog ligands, SHH has been most intensely studied. In brief, the SHH gradient formation is regulated by several cell surface proteins that bind to SHH and limit its distribution [14]. One of these proteins is the Hedgehog-interacting protein (HHIP), which can compete with PTCH1 for Hedgehog ligand binding, thus acting as a negative regulator of Hedgehog signaling [15]. HHIP can perform despite being near the SHH source, where SHH can activate the response by enhancing the degradation of HHIP, while HHIP retains its non-cell autonomous effects on the SHH response [14]. HHIP expression is down-regulated in a variety of tumors, such as esophageal, colorectal, pancreatic, and lung cancer. HHIP down-regulation is due to epigenetic CpG hypermethylation of the HHIP promoter [16–18]. Besides HHIP, also known as HHIP1, HHIP family contains another two members, HHIPL1 (HHIP2) and HHIPL2 (HHIP3) [19]. HHIPL1 is a secreted protein that interacts with SHH and is a positive regulator of SHH signaling in smooth muscle cells to aggravate atherosclerosis [20]. HHIP and HHIPL1 share a sequence homology with HHIP family genes but display a huge variance in their biological effects, which suggests that the exact functions of HHIPL2, as a hitherto uncharacterized gene, in the regulation of Hedgehog signaling and human cancers are almost unpredictable. Although HHIPL2 was reported to act as a potential biomarker for gastric cancer and lung squamous cell carcinoma (LUSC) [21, 22], whether and how HHIPL2 regulates the Hedgehog signaling pathway and the progression of human cancers, especially NSCLC, is totally undiscovered.

RNA-binding proteins (RBPs) are crucial regulatory components for cellular homeostasis, as they control RNA abundances and functions. HNRNPC, named as Heterogeneous nuclear ribonucleoproteins C1/C2 with localization in the nucleus and cytoplasm, acts as an RBP belonging to the hnRNP family, which controls multiple aspects of RNA metabolism, including alternative splicing, mRNA stabilization, and translation [23–25]. It is reported that HNRNPC plays a crucial role in various types of cancer. For instance, silencing of HNRNPC inhibits the metastasis potential of hepatocellular carcinoma (HCC) by decreasing HIF1A mRNA stability [26]. HNRNPC promotes esophageal squamous cell carcinoma (ESCC) proliferation and metastasis by increasing GLI2 mRNA stability [27]. HNRNPC promotes the proliferation and metastasis of NSCLC cells and is associated with CD8 + T cell infiltration [23].

Herein, we reported that HHIPL2 expression positively correlated with the Hedgehog signaling activity, and HHIPL2 was highly upregulated and associated with an unfavorable prognosis for NSCLC patients. We further demonstrated that HHIPL2 promoted NSCLC proliferation and metastasis by accelerating Sonic Hedgehog signaling. Moreover, we showed that HHIPL2 regulated Sonic Hedgehog signaling by enhancing the mRNA stability of Hepatocyte nuclear factor 1-alpha (HNF1A). We also verified that HHIPL2 handled HNF1A mRNA stability by interacting with HNRNPC and increasing its nucleo-cytoplasmic translocation. In addition, we discovered that an HNF1A inhibitor, triptolide, impeded HHIPL2-mediated Sonic Hedgehog signaling and progression. Our findings provide an option for targeting the HNF1A/Sonic Hedgehog signaling axis in NSCLC that may offer therapeutic benefits to patients with high HHIPL2 expression.

## Results

### HHIPL2 expression positively correlates with Hedgehog signaling activity

**Fig. 1:**
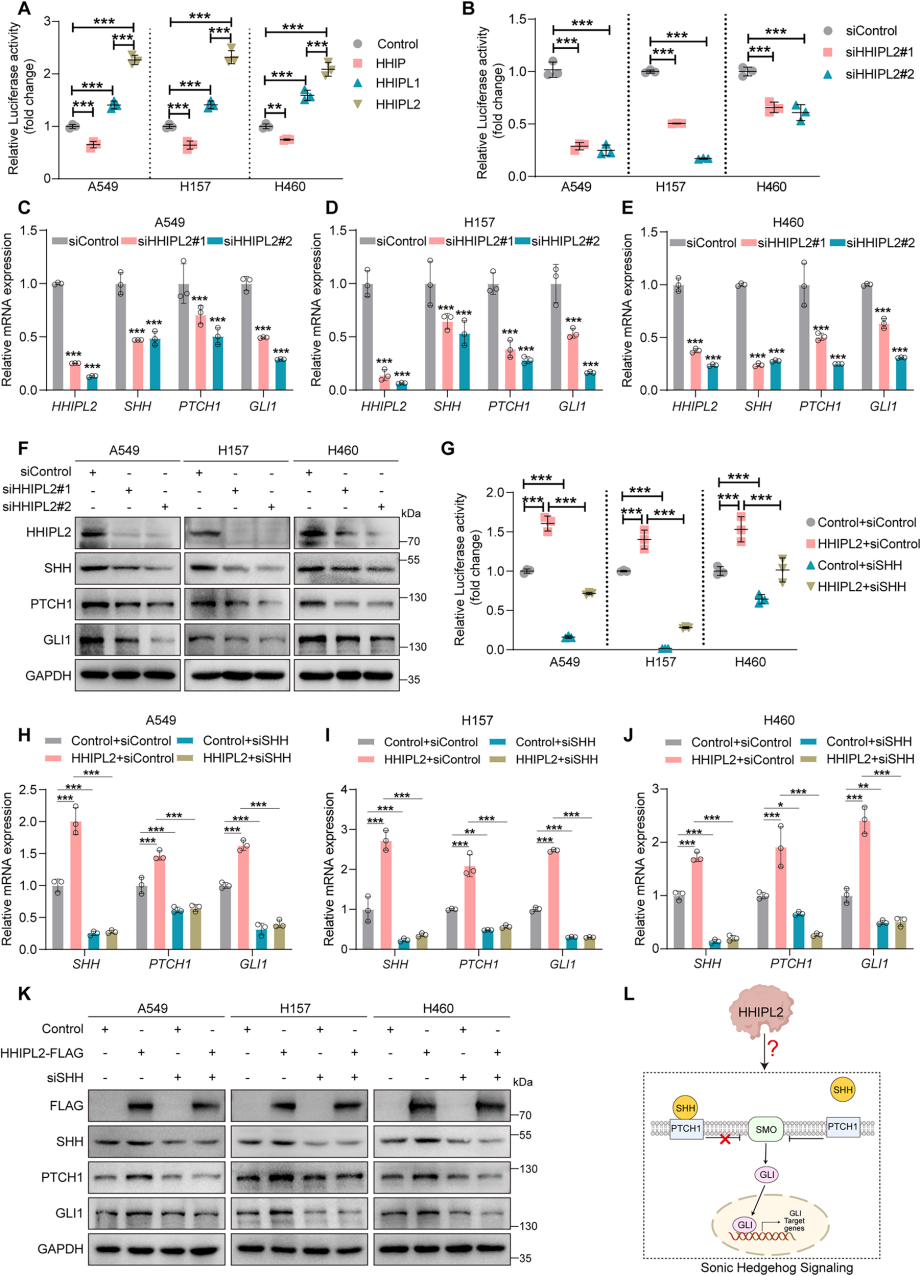
HHIPL2 expression positively correlates with Hedgehog signaling activity. **A** GLI luciferase assays of A549, H157, and H460 cells with HHIP, HHIPL1, and HHIPL2 overexpression. The GLI luciferase activity is normalized to Renilla (*n* = 3 in each group). **B** GLI luciferase assays of A549, H157, and H460 cells with HHIPL2 knockdown. The GLI luciferase activity is normalized to Renilla (*n* = 3 in each group). Relative RT-qPCR analysis of the mRNA levels of the genes related to the Hedgehog signaling in A549 (**C**), H157 (**D**), and H460 (**E**) cells with or without HHIPL2 knockdown (*n* = 3 in each group). **F** Knockdown of HHIPL2 in A549, H157, and H460 cells. Cell lysates were analyzed by Western blot. **G** GLI luciferase assays of A549, H157, and H460 cells overexpressing HHIPL2 and SHH knockdown. The GLI luciferase activity is normalized to Renilla (*n* = 3 in each group). Relative RT-qPCR analysis of the mRNA levels of the genes related to the Hedgehog signaling in A549 (**H**), H157 (**I**), and H460 (**J**) cells overexpressing HHIPL2 and SHH knockdown (*n* = 3 in each group). **K** Overexpression of HHIPL2 in A549, H157, and H460 cells with or without SHH knockdown. Cell lysates were analyzed by Western blot. **L** Schematic showing that HHIPL2, possibly via an unknown mechanism, regulates Sonic Hedgehog signaling. Sonic Hedgehog signaling is roughly as follows: in the absence of SHH, PTCH1 suppresses the activity of SMO, which is released upon SHH binding to PTCH1, allowing SMO to activate its downstream targets. GLI, as the transcription factor, translocates into the nucleus to promote the transcription of related genes, thereby activating Sonic Hedgehog signaling. Data in (**A**–**E, G**–**J**) are presented as the mean ± SD. Statistical significance was assessed by a one-way ANOVA. *P < 0.05, **P < 0.01, ***P < 0.001. Experiments (**A**–**K**) were repeated at least three times.

As mentioned above, it is reported that HHIP and HHIPL1 were involved in the Hedgehog signaling pathway, while it is unclear whether and how HHIPL2 regulates Hedgehog signaling. We first examined the protein level of HHIP, HHIPL1, and HHIPL2 in five different NSCLC cell lines and one lung epithelial cell line, and we chose A549 (a lung adenocarcinoma cell line), H157 (a lung squamous carcinoma cell line), and H460 (a lung large cell carcinoma cell line) cells for the experiments (Fig. S1A, B). Here, to investigate the role of HHIPL2 in Hedgehog signaling, we performed Hedgehog luciferase reporter assays [28, 29] in the condition of HHIP family members overexpression in A549, H157, and H460 cells (Fig. S1C). Among them, HHIP overexpression decreased the Hedgehog luciferase activity, while ectopic expression of HHIPL1 caused an enhanced luciferase activity, which is consistent with reported results [20]. Above all, we found that overexpression of HHIPL2 exhibited stronger positive effects to increase the Hedgehog luciferase activity compared to HHIPL1, suggesting that among the three members in the HHIP gene family, HHIPL2 is a predominantly positive regulator for the Hedgehog signaling pathway activation (Fig. 1A). To further verify the function of HHIPL2 in Hedgehog signaling in NSCLC cells, we knocked down HHIPL2 by transfecting small interfering RNA (siRNA) in A549, H157, and H460 cells. Hedgehog luciferase reporter assays showed that HHIPL2 knockdown dramatically inhibited the Hedgehog luciferase activity (Fig. 1B). In addition, in the three NSCLC cell lines with HHIPL2 depletion, our results showed that HHIPL2 positively decreased the mRNA levels of SHH, as a classical ligand to activate Sonic Hedgehog signaling, as well as PTCH1 and GLI1 (downstream targets of Sonic Hedgehog pathway) (Fig. 1C–E). On the contrary, overexpression of HHIPL2 increased the mRNA of SHH, PTCH1, and GLI1 (Fig. S1D–F). At the same time, we observed that HHIPL2 knockdown impaired the protein levels of SHH, PTCH1, and GLI1 (Fig. 1F). Conversely, overexpression of HHIPL2 remarkably upregulated the protein levels of essential molecules (SHH, PTCH1, and GLI1) in the Sonic Hedgehog signaling pathway (Fig. S1G). To further confirm that HHIPL2 regulates the Hedgehog luciferase activity by affecting the Sonic Hedgehog signaling, we knocked down SHH, a vital initiator for Sonic Hedgehog signaling activation, and used Cyclopamine (Cyc), an inhibitor of SMO, to inhibit the Sonic Hedgehog pathway. Our results showed that the Hedgehog luciferase activity was impaired in HHIPL2 overexpressed and SHH knockdown cells compared with the group of only HHIPL2 overexpression (Fig. 1G). The knockdown of SHH repressed HHIPL2-induced mRNA and protein upregulation of PTCH1 and GLI1 (Fig. 1H–K). Consistent results were obtained with Cyc treatment (Fig. S1H–L). Therefore, these results supported the idea that HHIPL2 actively regulates Sonic Hedgehog signaling in NSCLC (Fig. 1L).

### HHIPL2 is aberrantly elevated in NSCLC and promotes the proliferation and metastasis of NSCLC cells in vitro and in vivo

**Fig. 2:**
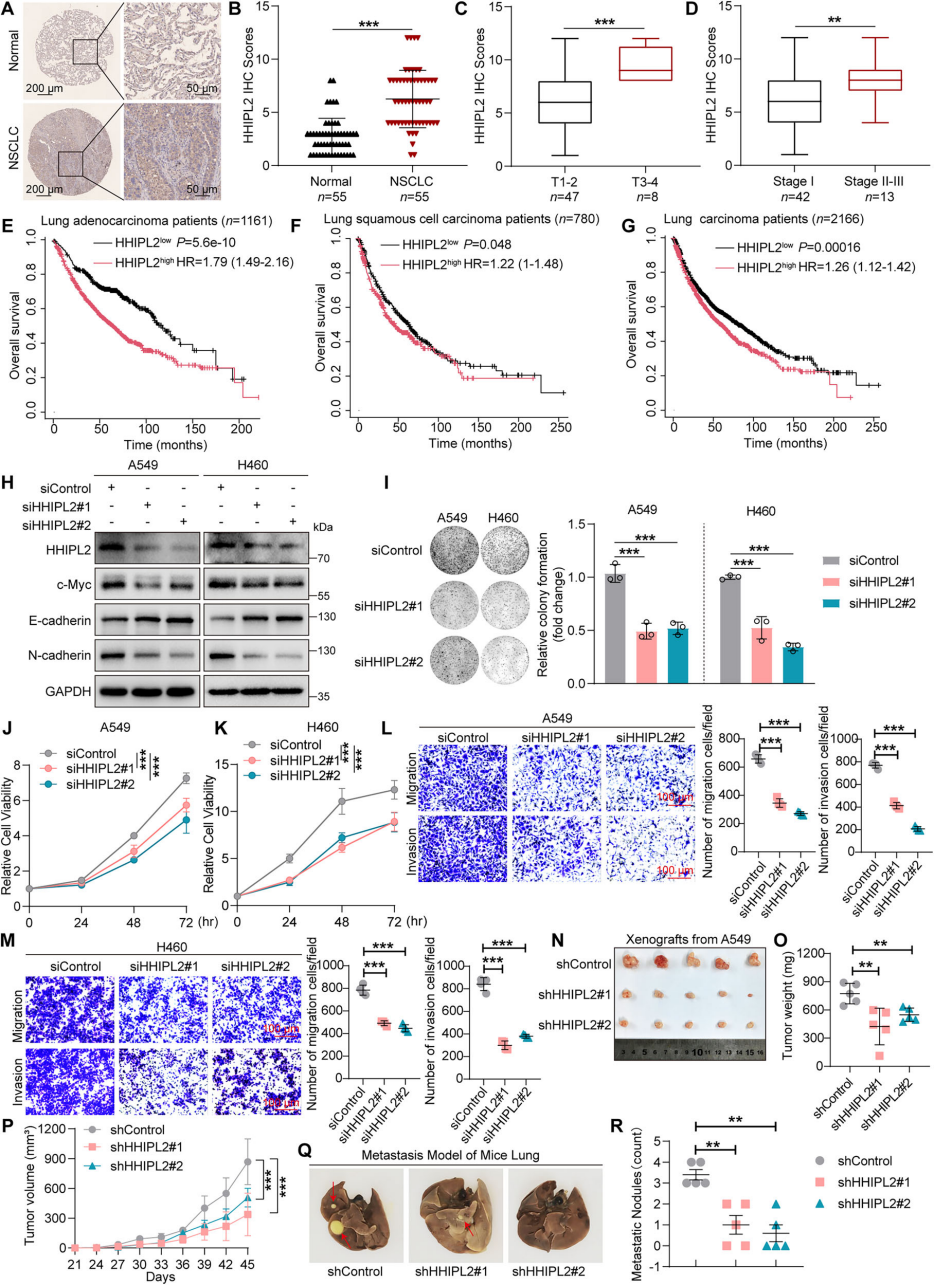
HHIPL2 is aberrantly elevated in NSCLC and promotes the proliferation and metastasis of NSCLC cells in vitro and in vivo. **A** Representative HHIPL2 IHC images of NSCLC tumor tissues and normal tissues (*n* = 55 per group). Scale bars as shown. **B** HHIPL2 IHC scores in NSCLC tumor tissues and normal tissues. HHIPL2 IHC scores of NSCLC tissues were compared between T1-2 and T3-4 (**C**) or Stage I and II-III (**D**). Kaplan–Meier plots of the overall survival of Lung adenocarcinoma patients (**E**), Lung squamous cell carcinoma patients (**F**), and Lung carcinoma patients (**G**) stratified by HHIPL2 expression. The data were acquired from the Kaplan-Meier plotter database. **H** Knockdown of HHIPL2 in A549 and H460 cells. Cell lysates were analyzed by western blotting with the indicated antibodies. **I** Colony formation assays in A549 and H460 cells with or without HHIPL2 knockdown. ImageJ was used to perform quantitative analysis (*n* = 3 in each group). **J**, **K** CCK-8 assays in A549 and H460 cells with or without HHIPL2 knockdown (*n* = 3 in each group). **L**, **M** Effects of HHIPL2 knockdown on migration and invasion in A549 and H460 cells using transwell assays. ImageJ was used to perform quantitative analysis (*n* = 4 in each group). Scale bar, 100 μm. Representative images of xenograft tumors after subcutaneous injection of A549 cells with HHIPL2 knockdown and controls (**N**), Tumor weights (**O**) and tumor volumes (**P**) were measured (*n* = 5 per group). Representative images of lung metastasis models in nude mice after tail injection of A549 cells with HHIPL2 knockdown (**Q**) and quantification of pulmonary metastatic nodules (**R**) (*n* = 5 per group). Data in (**B**–**D**, **I**–**M**, **O**, **P**, **R**) are presented as the mean ± SD. Statistical significance was assessed by the Wilcoxon matched-pairs signed rank test (**B**), the Mann–Whitney test (**C**, **D**), a one-way ANOVA (**I**, **L**, **M**, **O**, **R**), and a two-way ANOVA (**J**, **K**, **P**). **P < 0.01, ***P < 0.001. Experiments (**H**–**M**) were repeated at least three times.

We next investigated the protein levels of HHIPL2 in 55 pairs of surgically removed NSCLC specimens. The differences were dramatically reflected by the results of immunohistochemistry analysis, where upregulated protein expression of HHIPL2 was found in NSCLC tissues compared with adjacent normal lung tissues (Fig. 2A, B). Furthermore, a high HHIPL2 expression level was positively associated with the advanced stages of the primary tumor compared to those at the early stages (Fig. 2C, D). Detailed clinicopathological factors of 55 patients were summarized in Table S1. Moreover, our western blot assays showed that the HHIPL2 protein level increased in the tumor tissues compared with matched normal tissues in 5 pairs of NSCLC specimens (Fig. S2A). Based on the online database, using the Kaplan–Meier method followed by the log-rank test [30], we confirmed that higher expression of HHIPL2 correlated with shorter overall survival in NSCLC patients. More critically, HHIPL2 correlates with a worse prognosis in adenocarcinoma patients more than in squamous cell carcinoma patients (Fig. 2E–G). In addition, we explored the mRNA expression of HHIPL2 in NSCLC patients. Data from the UALCAN-TCGA online database [31, 32] showed HHIPL2 expression upregulated in lung adenocarcinoma (LUAD) and LUSC samples compared with normal lung tissues (Fig. S2B, C). In contrast to normal lung tissues, HHIPL2 expression was significantly elevated in both LUAD and LUSC with nodal metastasis. Notably, among cases exhibiting nodal metastasis, HHIPL2 expression levels were significantly higher in LUAD than in LUSC (Fig. S2D, E). Similarly, HHIPL2 expression was consistently upregulated in both LUAD and LUSC across all cancer stages compared to normal tissues, with higher levels observed in LUAD than in LUSC (Fig. S2F–H). Further analysis revealed that HHIPL2 expression was significantly higher in advanced stages (III-IV) compared to early stages (I-II) in LUAD, whereas no such difference was observed in LUSC. A combined analysis of LUAD and LUSC cases showed no significant difference in HHIPL2 expression between early (I-II) and advanced (III-IV) stages (Fig. S2I–K). Intriguingly, data from The Human Protein Atlas online database showed that mRNA expression of HHIPL2 was upregulated in the majority of human cancer cell lines compared to non-cancerous cell lines (Fig. S2L), indicating that higher expression of HHIPL2 is widespread in a pan-cancer manner. By analyzing the cBioPortal database [33], we identified that HHIPL2 is expressed across the majority of tumor types. Notably, its expression level is significantly elevated in LUAD compared to LUSC (Fig. S2M). Overall, these results indicated that HHIPL2 might play a facilitation role in NSCLC progression and is associated with poor prognosis.

According to the expression of HHIPL2 at protein levels in NSCLC cells and lung epithelial cells (Fig. S1B), we selected A549 and H460 cells, displaying a higher expression of HHIPL2 compared with other NSCLC cell lines, to perform the knockdown experiments. H157 cells, having lower protein levels of HHIPL2, were used to carry out overexpression experiments. Due to the wide utilization of A549, we also applied A549 to confirm the phenotypes of NSCLC cells with ectopic expression of HHIPL2. To examine the function of HHIPL2 in the progression of NSCLC cells, we carried out the depletion of HHIPL2 with siRNA, which resulted in a significant knockdown of HHIPL2 in A549 and H460 cells. We examined the protein expression levels of c-Myc, E-cadherin, and N-cadherin, which are essential for cell proliferation and metastasis. Western blot analysis indicated that HHIPL2 depletion occurred with the decreased protein levels of c-Myc and N-cadherin and upregulated expression of E-cadherin (Fig. 2H). Meanwhile, the colony formation assays and CCK-8 assays showed that the knockdown of HHIPL2 impaired NSCLC cell proliferation (Fig. 2I–K). Moreover, transwell assays showed a significantly reduced migration and invasion capability in HHIPL2-knockdown cells (Fig. 2L, M). In addition, we established A549 cell lines with stable HHIPL2 knockdown using the lentivirus containing shRNAs against HHIPL2 (shHHIPL2#1 and shHHIPL2#2) or vector control (shControl) (Fig. S3A). The tumor xenograft model was used to examine the effect of HHIPL2 knockdown on tumor growth. HHIPL2 knockdown led to much slower growth and smaller volume and weight in xenograft tumors than the control group (Fig. 2N–P), which was further supported by a remarkably reduced Ki67 expression in HHIPL2-knockdown tumors (Fig. S3B). We further evaluated the alteration induced by HHIPL2-knockdown on the ability of A549 cells to colonize the lung and form metastasis nodules when injecting the cells into the mouse caudal vein. As shown in Fig. 2Q, R, HHIPL2 depletion significantly reduced metastatic nodules compared with the control group. Correspondingly, H&E-stained sections of the lungs revealed a marked reduction of metastasis volume in the HHIPL2-knockdown group (Fig. S3C).

We further explored the consequences caused by HHIPL2 overexpression in NSCLC cells. As shown in Fig. S3D, we constructed stable HHIPL2 overexpressing cell lines in A549 and H157 cells. Exogenous expression of HHIPL2 consistently upregulated the protein levels of c-Myc and N-cadherin but downregulated the expression of E-cadherin protein. The CCK-8 assays showed that HHIPL2 overexpression facilitated cell proliferation (Fig. S3E, F). Meanwhile, the colony formation assays indicated that HHIPL2 overexpression remarkably increased the number of colonies derived from A549 and H157 cells (Fig. S3G). The transwell assays further demonstrated that HHIPL2 overexpression promoted migration and invasion ability (Fig. S3H–K). As expected, the HHIPL2-overexpression group displayed faster xenograft growth, larger tumor volume and weight, and increased Ki67 protein levels compared to the control group (Fig. S3L–O). Additionally, overexpression of HHIPL2 significantly increased lung metastasis nodules compared to the control group (Fig. S3P). H&E-stained sections of the lungs revealed a marked increase in metastasis volume in the HHIPL2-overexpression group (Fig. S3Q). Our results imply that dysregulation of HHIPL2 aggravates NSCLC progression by promoting proliferation and metastasis in vitro and in vivo.

### HHIPL2 accelerates NSCLC progression by positively regulating Sonic Hedgehog signaling

**Fig. 3:**
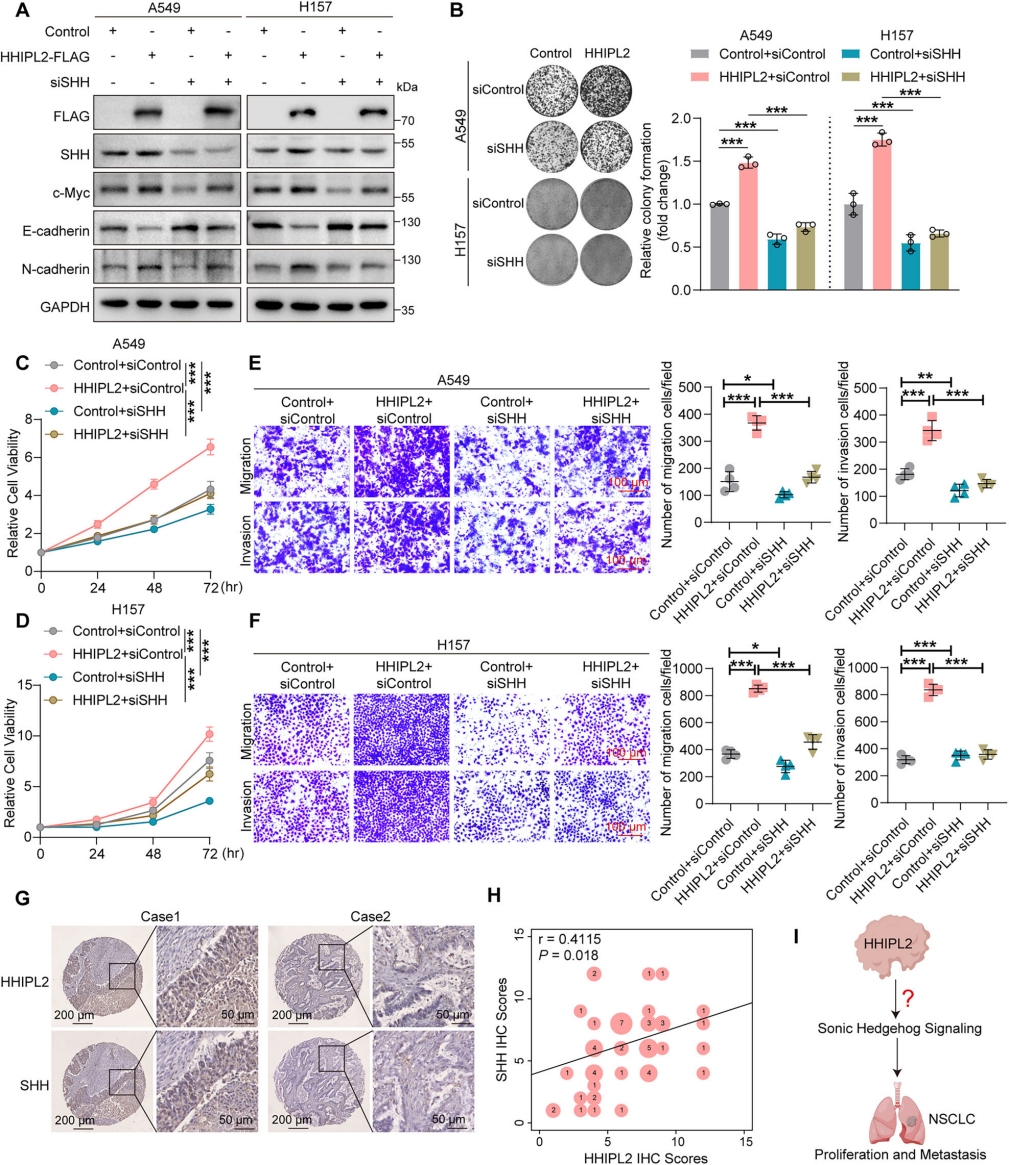
HHIPL2 accelerates NSCLC progression by positively regulating Sonic Hedgehog signaling. **A** Overexpression of HHIPL2 in A549 and H157 cells with or without SHH knockdown. Cell lysates were analyzed by Western blot. **B** Effects of HHIPL2 overexpression with or without SHH knockdown in A549 and H157 cells using colony formation assays. ImageJ was used to perform quantitative analysis (*n* = 3 in each group). Effects of HHIPL2 overexpression with or without SHH knockdown in A549 (**C**) and H157 (**D**) cells using CCK-8 assays (*n* = 3 in each group). Effects of HHIPL2 overexpression with or without SHH knockdown on migration and invasion in A549 (**E**) and H157 (**F**) cells using transwell assays. ImageJ was used to perform quantitative analysis (*n* = 4 in each group). Scale bar, 100 μm. **G** HHIPL2 expression was highly correlated with SHH expression in NSCLC tissues analyzed by IHC staining. Scale bars as shown. **H** Spearman correlation plot of HHIPL2 and SHH IHC scores in 55 NSCLC tissue samples. **I** Schematic showing that HHIPL2 accelerates NSCLC progression by regulating Sonic Hedgehog signaling. Data in (**B**–**F**) are presented as the mean ± SD. Statistical significance was assessed by a one-way ANOVA(**B**, **E**, **F**), a two-way ANOVA (**C**, **D**), and a Spearman’s rank correlation coefficient analysis (**H**). *P < 0.05, **P < 0.01, ***P < 0.001. Experiments (**A**–**F**) were repeated at least three times.

To further investigate the role of the Sonic Hedgehog signaling pathway in HHIPL2-promoted NSCLC proliferation and metastasis, we first evaluated the protein levels of c-Myc, E-cadherin, and N-cadherin upon SHH knockdown. Our data showed that HHIPL2-induced upregulation of c-Myc and N-cadherin and downregulation of E-cadherin was rescued by SHH depletion (Fig. 3A). In addition, knockdown of SHH significantly suppressed the characteristics of HHIPL2-accelerated cell colony formation (Fig. 3B). The CCK-8 assays showed that SHH knockdown partially attenuated the growth effect induced by HHIPL2 overexpression (Fig. 3C, D). Moreover, the transwell assays indicated that increased migration and invasion capacity induced by HHIPL2 overexpression could be decreased when SHH was knocked down (Fig. 3E, F). In addition, we also observed that the inhibiting effects of Cyc treatment on cell proliferation, migration, and invasion were pronounced in HHIPL2-overexpression (Fig. S4A–F). These data suggest that HHIPL2 promotes NSCLC progression by activating the Sonic Hedgehog signaling pathway.

To evaluate the clinical significance and correlation between HHIPL2 and Sonic Hedgehog signaling, we first analyzed the protein expression of SHH, a vital initiator for Sonic Hedgehog signaling activation, in 55 NSCLC patients’ tumors and normal tissues. Immunohistochemical evaluation indicated that SHH protein levels increased in tumors compared with normal lung tissues (Fig. S4G), and high SHH protein levels correlated with Stage II-III of NSCLC (Fig. S4H). Besides, we found that the patients with high HHIPL2 levels often displayed high SHH expression and vice versa, indicating that the expression of HHIPL2 positively correlated with that of the SHH protein (Fig. 3G, H). Furthermore, our tumor sections showed that SHH protein levels decreased remarkably in HHIPL2-knockdown groups and vice versa by immunohistochemical staining (Fig. S4I, J). Therefore, HHIPL2 accelerates NSCLC progression by positively regulating Sonic Hedgehog signaling. Next, we sought to uncover the underlying mechanism of HHIPL2 regulating Sonic Hedgehog signaling (Fig. 3I).

### HHIPL2 regulates the Sonic Hedgehog signaling and NSCLC progression through HNF1A

**Fig. 4:**
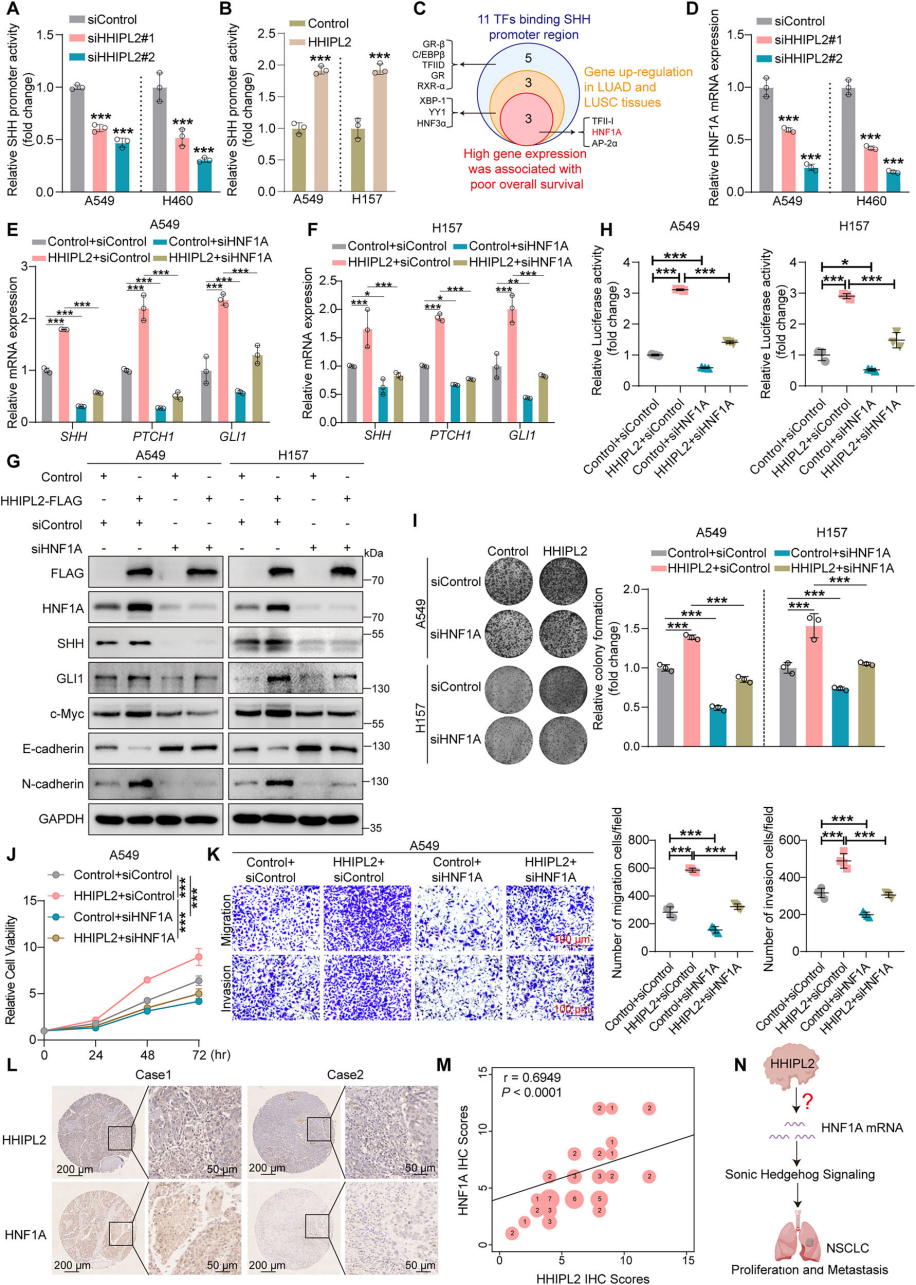
HHIPL2 regulates the Sonic Hedgehog signaling and NSCLC progression through HNF1A. **A** The dual-luciferase reporter assays of A549 and H460 cells analysis of the SHH promoter activity with HHIPL2 knockdown. The luciferase activity is normalized to Renilla (*n* = 3 in each group). **B** The dual-luciferase reporter assays of A549 and H157 cells analysis of the SHH promoter activity with HHIPL2 overexpression. The luciferase activity is normalized to Renilla (*n* = 3 in each group). **C** Schematic showing that the screening process of three transcription factors (TFs) binds the SHH promoter region. **D** Relative RT-qPCR analysis of the mRNA levels of HNF1A in A549 and H460 cells with or without HHIPL2 knockdown (*n* = 3 in each group). Relative RT-qPCR analysis of the mRNA levels of the genes related to the Sonic Hedgehog pathway in A549 (**E**) and H157 cells (**F**) with HHIPL2 overexpression and HNF1A knockdown (*n* = 3 in each group). **G** Overexpression of HHIPL2 in A549 and H157 cells with or without HNF1A knockdown. Cell lysates were analyzed by Western blot. **H** GLI luciferase assays of A549 and H157 cells with HHIPL2 overexpression and HNF1A knockdown. The GLI luciferase activity is normalized to Renilla (*n* = 3 in each group). **I** Effects of HHIPL2 overexpression with or without HNF1A knockdown in A549 and H157 cells using colony formation assays. ImageJ was used to perform quantitative analysis (*n* = 3 in each group). **J** Effects of HHIPL2 overexpression with or without HNF1A knockdown in A549 cells using CCK-8 assays (*n* = 3 in each group). **K** Effects of HHIPL2 overexpression with or without HNF1A knockdown on migration and invasion in A549 cells using transwell assays. ImageJ was used to perform quantitative analysis (*n* = 4 in each group). Scale bar, 100 μm. **L** HHIPL2 expression was highly correlated with HNF1A expression in NSCLC tissues analyzed by IHC staining. Scale bars as shown. **M** Spearman correlation plot of HHIPL2 and HNF1A IHC scores in 55 NSCLC tissue samples. **N** Schematic showing that HHIPL2 regulates Sonic Hedgehog signaling and NSCLC progression by HNF1A. Data in (**A**, **B**, **D**–**H**, **I**–**K**) are presented as the mean ± SD. Statistical significance was assessed by a two-sided Student’s *t* test (**B**), a one-way ANOVA (**A**, **D**–**F**, **H**, **I**, **K**), a two-way ANOVA(**J**), and a Spearman’s rank correlation coefficient analysis (**M**). *P < 0.05, **P < 0.01, ***P < 0.001. Experiments (**A**, **B**, **D**–**K**) were repeated at least three times.

Our earlier findings have implied that HHIPL2 regulates SHH, the upstream initiator of Sonic Hedgehog signaling. We hypothesized that HHIPL2 might upregulate the mRNA of SHH to accelerate Sonic Hedgehog signaling. To investigate the potential role of HHIPL2 on SHH mRNA, we first evaluated the transcriptional activity of the SHH promoter through a dual-luciferase assay, and the results showed that SHH transcriptional activity was decreased when HHIPL2 was knocked down, and vice versa, overexpression of HHIPL2 induced increased SHH transcriptional activity (Fig. 4A, B). Consistently, SHH mRNA and protein expression levels showed this trend when HHIPL2 was knocked down or overexpressed (Figs. 1C–F and S1D–G). Next, we used the PROMO website [34, 35] to predict the possible transcription factors (TFs) binding to the SHH promoter region. As shown in Fig. 4C, among 11 transcription factors predicted by PROMO, six transcription factor genes were upregulated in lung adenocarcinoma and lung squamous cell carcinoma based on UALCAN-TCGA online database [31, 32] (Fig. S5A-T). We next analyzed the effects of six transcription factors on overall survival by the Kaplan–Meier Plotter website [30]. Only three transcription factors (TFII-I, HNF1A, AP-2α) exhibited that high expression was associated with poor overall survival among lung carcinoma patients (Fig. S6A–J). After RT-qPCR and western blot validation, it was found that the knockdown of HHIPL2 resulted in the reduction of HNF1A mRNA levels, whereas HHIPL2-overexpression upregulated HNF1A mRNA levels. Consistently, the protein levels of HNF1A decreased when we used HHIPL2 siRNA to knock down HHIPL2 and vice versa (Figs. 4D and S7A–C). However, we did not observe any change in the mRNA and protein levels of TFII-I and AP-2α after HHIPL2 knockdown or overexpression (Fig. S7D–I). These findings suggested that HHIPL2 positively regulated HNF1A expression, most likely occurring at the mRNA levels. Interestingly, our previous study showed that HNF1A acted as a transcription factor of SHH to activate the Sonic Hedgehog signaling pathway [36]. We also observed that the mRNA levels of SHH could no longer be upregulated by HHIPL2 when HNF1A was knocked down (Fig. 4E, F). These findings suggested that HHIPL2 was most likely to positively regulate SHH by affecting HNF1A mRNA levels.

To explore the role of HNF1A in HHIPL2-activated Sonic Hedgehog signaling and oncogenicity, we first evaluated the mRNA and protein expression of Sonic Hedgehog signaling markers upon HNF1A knockdown. Our results showed that knockdown of HNF1A downregulated the mRNA and protein expression of Sonic Hedgehog signaling markers in HHIPL2-overexpression cells (Fig. 4E–G). Notably, the knockdown of HNF1A impaired the higher GLI luciferase activity induced by overexpressing HHIPL2 (Fig. 4H). Blocking assays were conducted to determine if HHIPL2 promoted NSCLC cell proliferation and metastasis through upregulating HNF1A. The proliferation and EMT-related markers could no longer be regulated by HHIPL2 when HNF1A was knocked down (Fig. 4G). Moreover, when we knocked down HNF1A, HHIPL2 did not increase cell proliferation, migration, and invasion (Figs. 4I–K and S7J, K). We further analyzed the levels of HNF1A in the 55 pairs of NSCLC specimens by immunohistochemical staining. The statistical results showed that the levels of HNF1A were significantly higher in NSCLC tissues compared with normal lung tissues (Fig. S7L). Elevated levels of HNF1A also correlated with stage II–III (Fig. S7M). The levels of HNF1A were positively associated with SHH protein expression in 55 NSCLC tumor tissues (Fig. S7N), which was consistent with our published study [36]. In addition, we found that the protein levels of HHIPL2 positively correlated with HNF1A expression (Fig. 4L, M). Meanwhile, the xenograft models revealed that the protein expression of HNF1A was decreased in HHIPL2-knockdown groups or increased in HHIPL2-overexpression groups by immunohistochemical staining (Fig. S7O, P). In summary, these results reveal that HHIPL2 upregulates the mRNA expression of HNF1A and promotes NSCLC progression through HNF1A-mediated Sonic Hedgehog signaling. Then, we sought to reveal the molecular mechanism of HHIPL2 regulating HNF1A mRNA (Fig. 4N).

### HHIPL2 controls the mRNA stability of HNF1A by interacting with HNRNPC

**Fig. 5:**
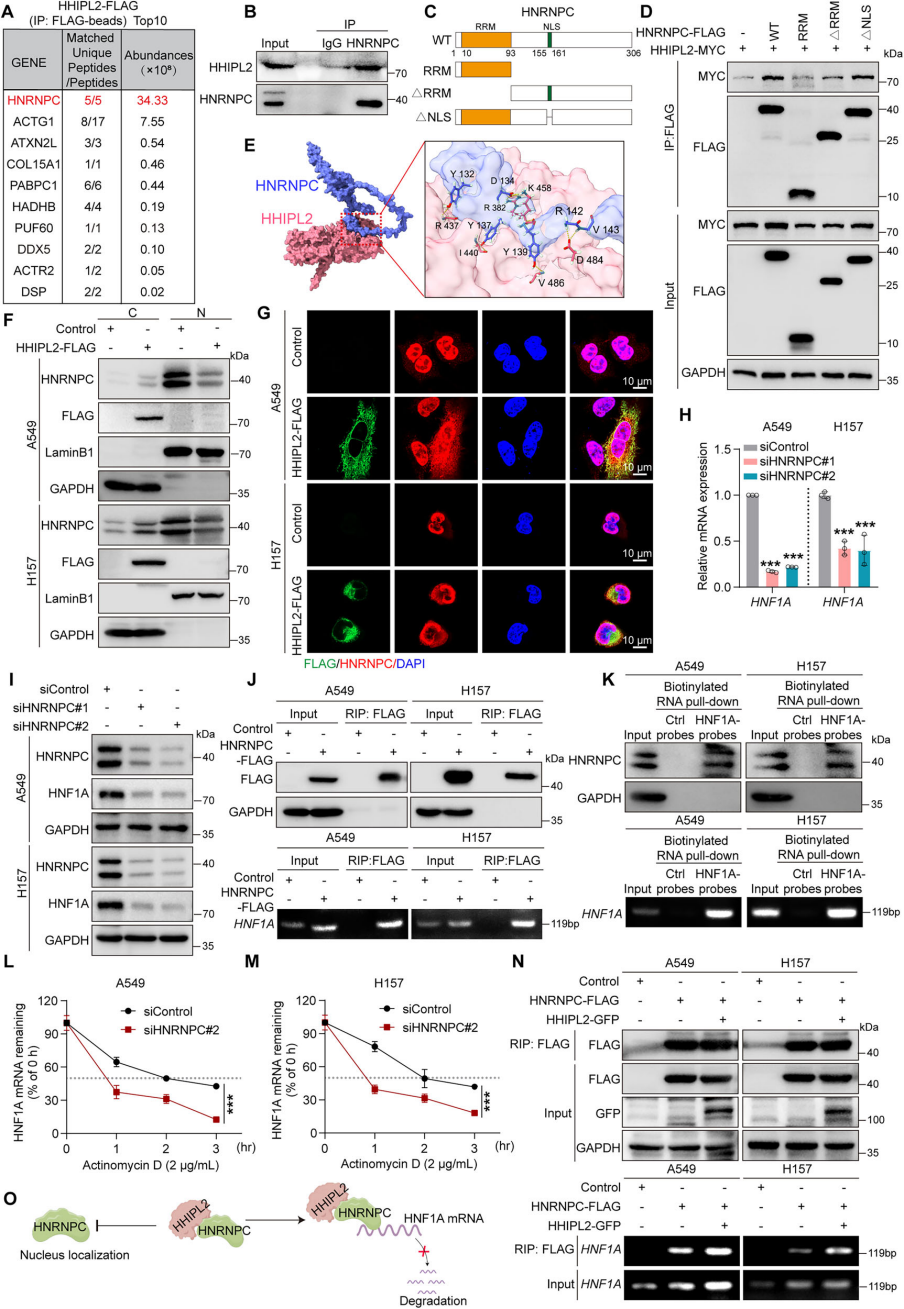
HHIPL2 controls the mRNA stability of HNF1A by interacting with HNRNPC. **A** Tandem affinity purification-mass spectrometry detection of HHIPL2-interacting proteins (obtained from FLAG-beads pull down) after A549 cells were transfected with HHIPL2-FLAG for 48 h. **B** Cell lysates of A549 cells were immunoprecipitated with IgG or HNRNPC antibodies, and immunoblot assays were performed using HHIPL2 and HNRNPC antibodies. **C** Schematic diagram of HNRNPC and the domain-deleted constructs. RRM, RNA recognition motif; NLS, nuclear localization signal. **D** Plasmids containing WT, RRM, ΔRRM, and ΔNLS of HNRNPC were co-expressed with HHIPL2-MYC in HEK293T cells. Lysates were immunoprecipitated with FLAG beads. **E** Best predicted pose of HHIPL2 and HNRNPC, with a prominent ZDOCK score of 1731.296. Active residues forming hydrogen bonds were indicated. **F** Cellular fractionation analysis of A549 and H157 cells overexpressing HHIPL2-FLAG. Cell fractions were analyzed by Western blot. C cytosol, N nucleus. **G** Immunofluorescence (IF) staining for endogenous HNRNPC and FLAG in A549 and H157 cells overexpressing FLAG-tagged HHIPL2. Nuclei were stained with DAPI. Scale bar, 10 μm. **H** Relative RT-qPCR analysis of the mRNA levels of HNF1A in A549 and H157 cells with or without HNRNPC knockdown (*n* = 3 in each group). **I** Knockdown of HNRNPC in A549 and H157 cells. Cell lysates were analyzed by western blotting with antibodies against HNRNPC, HNF1A, and GAPDH. **J** Binding of HNRNPC protein with HNF1A mRNA was determined by RNA immunoprecipitation (RIP) with FLAG beads in A549 cells and H157 cells overexpressing HNRNPC-FLAG. HNF1A mRNA enrichment was measured by RT-PCR analyses using the relevant primers for *HNF1A*. **K** Binding of HNF1A mRNA with HNRNPC protein was determined by RNA pull-down with HNF1A-probe in A549 and H157 cells. HNRNPC protein enrichment was measured by western blotting, and HNF1A mRNA enrichment was measured by RT-PCR analyses using the relevant primers for *HNF1A*. **L**, **M** The half-life of HNF1A mRNA was examined by RT-qPCR in A549 and H157 cells after HNRNPC knockdown and actinomycin D treatment (2 μg/mL) for the indicated times (*n* = 3 in each group). **N** Binding of HNRNPC protein with HNF1A mRNA was determined by RNA immunoprecipitation (RIP) with FLAG beads in A549 and H157 cells overexpressing HNRNPC-FLAG and HHIPL2-GFP. HNF1A mRNA enrichment was measured by RT-PCR analyses using the relevant primers for *HNF1A*. **O** Schematic showing that HNRNPC controls HNF1A mRNA stability by HHIPL2-mediated HNRNPC nucleo-cytoplasmic translocation. Data in (**H**, **L**, **M**) are presented as the mean ± SD. Statistical significance was assessed by a one-way ANOVA (**H**) and a two-way ANOVA(**L**, **M**). ***P < 0.001. Experiments (**B**, **D**–**N**) were repeated at least three times.

We reasoned that HHIPL2 increased HNF1A mRNA levels by either activating HNF1A transcription or enhancing its mRNA stabilization, in which HHIPL2 is almost impossible to exercise these regulations directly due to its lack of domains with relevant functions. Hence, revealing the potential partners’ binding to HHIPL2 is crucial to figuring out the molecular mechanism underlying HHIPL2-induced HNF1A mRNA alteration. To this end, we isolated the HHIPL2-associated protein complex through tandem affinity purification followed by mass spectrometry analysis. Interestingly, HNRNPC, an RNA-binding protein (RBP), was efficiently copurified with HHIPL2 in the prey list (Fig. 5A). We thus hypothesized that HHIPL2 might interact with HNRNPC to regulate HNF1A mRNA stabilization in NSCLC cells. We first evaluated the interaction between HHIPL2 and HNRNPC in HEK293T cells by Co-immunoprecipitations (Co-IPs). As shown in Fig. S8A, B, exogenously expressed either HNRNPC or HHIPL2 showed the interaction of HHIPL2 and HNRNPC. Moreover, endogenous HNRNPC protein was present in immunoprecipitates with antibodies against endogenous HHIPL2 (Fig. 5B). To identify the specific region of HNRNPC that interacts with HHIPL2, we prepared three truncated mutants of HNRNPC, a fragment containing only the RNA recognition (RRM, residues 1–93 aa, designated HNRNPC-RRM), a fragment lacking the RRM domain (residues 94–306 aa, designated HNRNPC-ΔRRM), and a construct with the nuclear localization signal (NLS) deleted (designated HNRNPC-ΔNLS) (Fig. 5C). HHIPL2-MYC and full-length or truncated HNRNPC with different deletions were co-transfected into HEK293T cells. Co-IPs demonstrated that containing only the RRM domain blocked the interaction between HHIPL2 and HNRNPC, indicating that the non-RRM domain of HNRNPC might be critical for its interaction with HHIPL2 (Fig. 5D). Meanwhile, molecular docking analysis also revealed that HHIPL2 interacted with HNRNPC through the non-RRM domain (Fig. 5E). In addition, we found that the knockdown or overexpression of HHIPL2 could not affect the protein level of HNRNPC (Fig. S8C, D). Previous studies showed that HNRNPC, an RNA-binding protein, could be localized in the cytoplasm and nucleus to regulate roles [23, 26]. We further speculated that HHIPL2 might be essential for HNRNPC nucleo-cytoplasmic translocation. Interestingly, overexpression of HHIPL2 resulted in increased levels of HNRNPC in the cytoplasm and decreased levels in the nucleus (Fig. 5F). Similar results were also observed in the immunofluorescence assay, where more endogenous HNRNPC colocalized with HHIPL2 in the cytoplasm upon HHIPL2 overexpression (Fig. 5G), suggesting that HHIPL2 promoted the localization of HNRNPC in the cytoplasm. These results showed that the interaction of HHIPL2 with HNRNPC increases the localization of HNRNPC in the cytoplasm.

In light of these findings, we hypothesized that HHIPL2 could increase the expression of HNF1A by enhancing HNF1A mRNA stability through cytoplasm-localized HNRNPC. To verify our hypothesis, we first used HNRNPC siRNA to inhibit the expression of HNRNPC in A549 and H157 cells (Fig. S8E), and the results showed that the knockdown of HNRNPC decreased HNF1A expression, especially its mRNA levels, and vice versa, overexpression of HNRNPC resulted in elevated mRNA and protein expression levels of HNF1A (Figs. 5H, I and S8F, G). We further undertook RNA pull-down and RNA immunoprecipitation (RIP) assays to validate the interaction between the HNRNPC protein and HNF1A mRNA. Those data showed that the HNRNPC protein physically interacted with HNF1A mRNA (Fig. 5J, K). In addition, to confirm that HNRNPC changed the transcript abundance of HNF1A by influencing its mRNA stability, Actinomycin D, an RNA synthesis inhibitor, was used to block de novo mRNA synthesis. The half-life of HNF1A mRNA was markedly decreased in the HNRNPC-knockdown cells, indicating that downregulation of HNRNPC decreased HNF1A expression by destabilizing HNF1A mRNA (Fig. 5L, M). Then, we found that the interaction between the HNRNPC protein and the HNF1A mRNA was enhanced by HHIPL2 overexpression and impaired when HHIPL2 was knocked down (Figs. 5N and S8H). We further explored whether HHIPL2 upregulates HNF1A through HNRNPC. To conclude, these results support the crucial role of HNRNPC in controlling HNF1A mRNA stability by HHIPL2-mediated HNRNPC nucleo-cytoplasmic translocation (Fig. 5O).

### HHIPL2 positively regulates Sonic Hedgehog signaling and promotes NSCLC progression via the HNRNPC/HNF1A axis

**Fig. 6:**
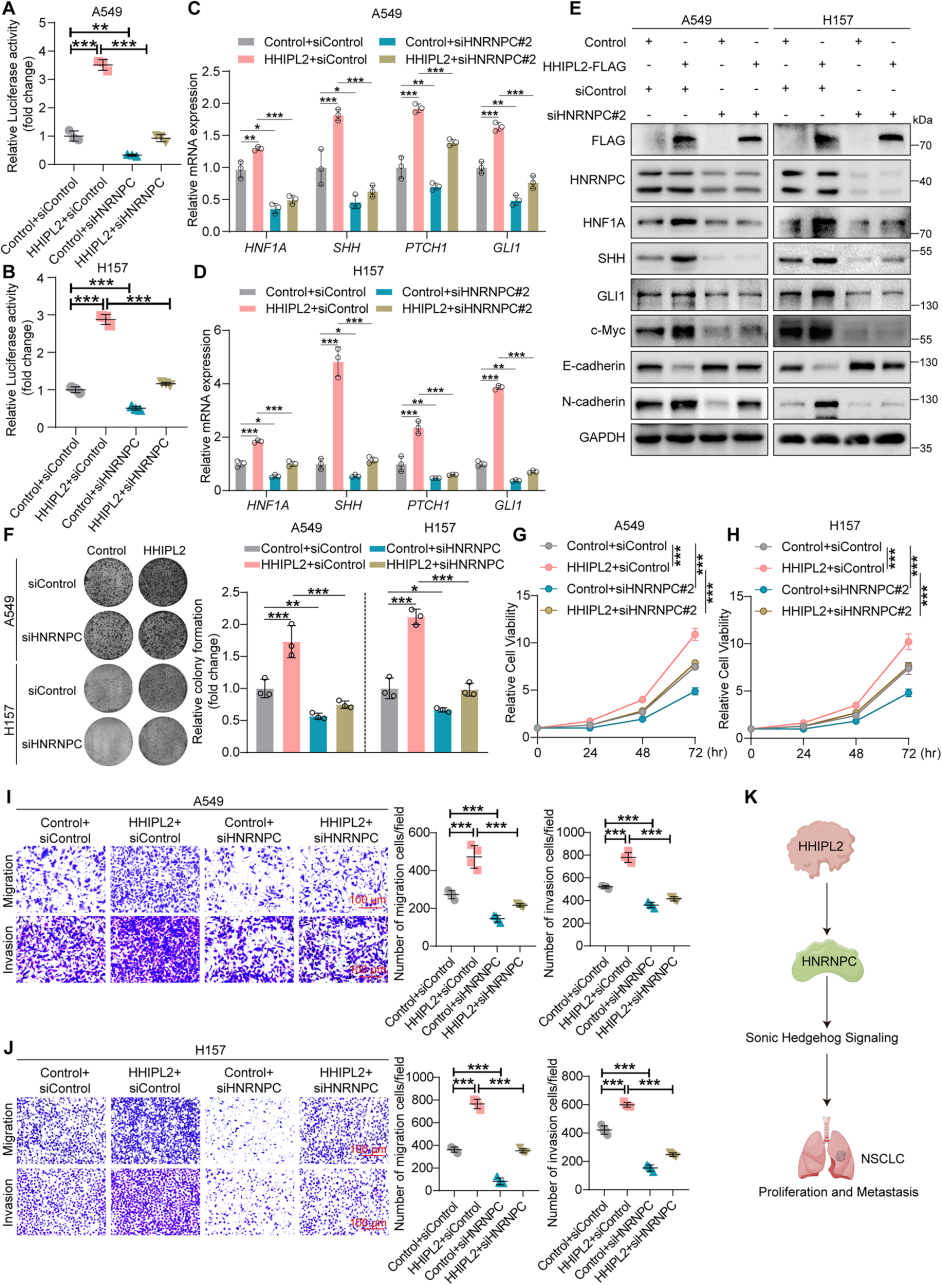
HHIPL2 positively regulates Sonic Hedgehog signaling and promotes NSCLC progression through HNRNPC. Effects of HHIPL2 overexpression with or without HNRNPC knockdown in A549 (**A**) and H157 cells (**B**) using GLI luciferase assays. The GLI luciferase activity is normalized to Renilla (*n* = 3 in each group). Relative RT-qPCR analysis of the mRNA levels of the genes related to the Sonic Hedgehog pathway in A549 (**C**) and H157 cells (**D**) with HHIPL2 overexpression and HNRNPC knockdown (*n* = 3 in each group). **E** Overexpression of HHIPL2 in A549 and H157 cells with or without HNRNPC knockdown. Cell lysates were analyzed by western blotting with the indicated antibodies. **F** Effects of HHIPL2 overexpression with or without HNRNPC knockdown in A549 and H157 cells using Colony formation assays. ImageJ was used to perform quantitative analysis (*n* = 3 in each group). Effects of HHIPL2 overexpression with or without HNRNPC knockdown in A549 (**G**) and H157 cells (**H**) using CCK-8 assays (*n* = 3 in each group). Effects of HHIPL2 overexpression with or without HNRNPC knockdown on migration and invasion in A549 (**I**) and H157 cells (**J**) using transwell assays and ImageJ was used to perform quantitative analysis (*n* = 4 in each group). Scale bar, 100 μm. **K** Schematic showing that HHIPL2 regulates Sonic Hedgehog signaling and NSCLC progression by HNRNPC. Data in (**A**–**D**, **F**–**J**) are presented as the mean ± SD. Statistical significance was assessed by a one-way ANOVA (**A**–**D**, **F**, **I**, **J**) and a two-way ANOVA(**G**, **H**). *P < 0.05, **P < 0.01, ***P < 0.001. Experiments (**A**–**J**) were repeated at least three times.

To explore the role of the HNRNPC in HHIPL2-driven Sonic Hedgehog signaling and oncogenicity in NSCLC cells, we first investigated the role of HNRNPC in Sonic Hedgehog signaling, proliferation, and metastasis of NSCLC cells. As shown in Fig. S9A, silencing of HNRNPC inhibited Sonic Hedgehog signaling protein expression. Furthermore, the knockdown of HNRNPC dramatically inhibited the protein levels of c-Myc and N-cadherin and increased E-cadherin expression. Conversely, overexpression of HNRNPC positively regulated the expression of SHH, GLI1, c-Myc, and N-cadherin, while HNRNPC negatively regulated E-cadherin levels (Fig. S9B). Next, we examined whether HNRNPC plays a role in HHIPL2-mediated regulation of Sonic Hedgehog signaling and NSCLC progression. As shown in Fig. 6A, B, the knockdown of HNRNPC dramatically impaired HHIPL2-mediated higher GLI luciferase activity. In addition, the knockdown of HNRNPC repressed HHIPL2-induced mRNA upregulation of HNF1A, SHH, PTCH1, and GLI1 (Fig. 6C, D). Silencing of HNRNPC markedly blocked HHIPL2 overexpression-induced changes in the expression of proteins, which were related to Sonic Hedgehog signaling, proliferation, and metastasis (Fig. 6E). These results suggested that HHIPL2 positively regulates the Sonic Hedgehog signaling dependent on HNRNPC. We then investigated the role of HNRNPC in HHIPL2-driven proliferation, migration, and invasion of NSCLC cells. Colony formation assays and CCK-8 assays showed that silencing of HNRNPC significantly inhibited HHIPL2 overexpression-induced proliferation (Fig. 6F–H). Significantly, silencing of HNRNPC blocked the increase in migration and invasion caused by HHIPL2 overexpression (Fig. 6I, J), which was consistent with western blot analyses (Fig. 6E). These data demonstrate that HHIPL2 positively regulated Sonic Hedgehog signaling and promoted NSCLC progression through HNRNPC (Fig. 6K).

**Fig. 7:**
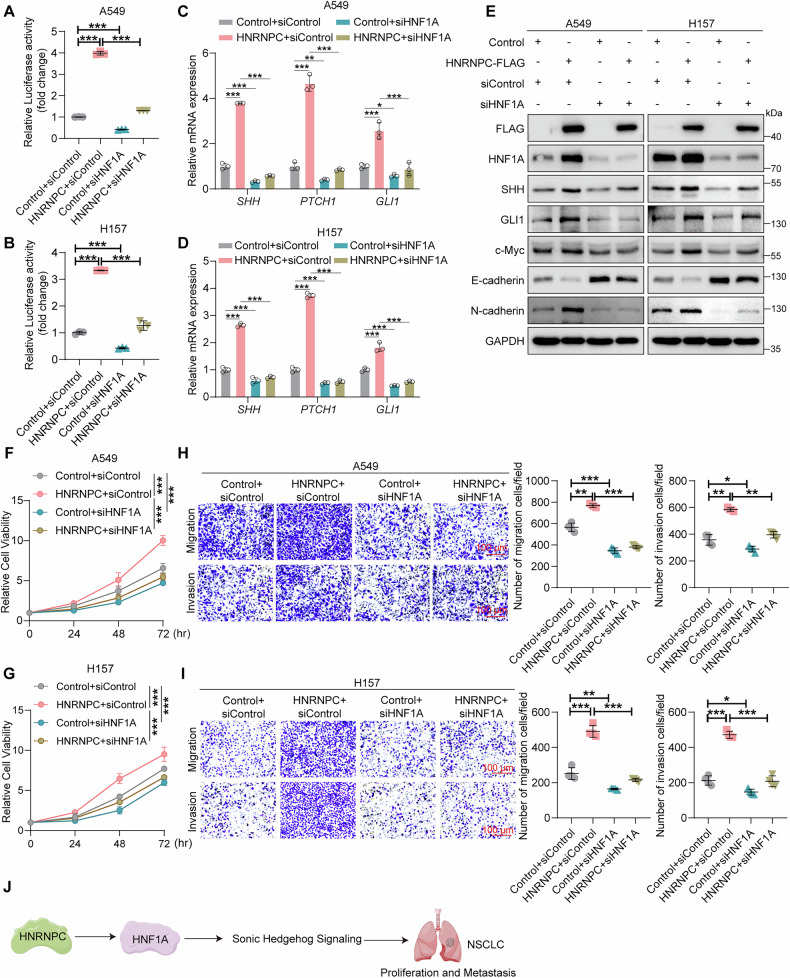
HNRNPC regulates Sonic Hedgehog signaling and promotes NSCLC progression through HNF1A. Effects of HNRNPC overexpression with or without HNF1A knockdown in A549 (**A**) and H157 cells (**B**) using GLI luciferase assays. The GLI luciferase activity is normalized to Renilla (*n* = 3 in each group). Relative RT-qPCR analysis of the mRNA levels of the genes related to the Sonic Hedgehog pathway in A549 (**C**) and H157 cells (**D**) with HNRNPC overexpression and HNF1A knockdown (*n* = 3 in each group). **E** Overexpression of HNRNPC in A549 and H157 cells with or without HNF1A knockdown. Cell lysates were analyzed by western blotting with the indicated antibodies. Effects of HNRNPC overexpression with or without HNF1A knockdown in A549 (**F**) and H157 cells (**G**) using CCK-8 assays (*n* = 3 in each group). Effects of HNRNPC overexpression with or without HNF1A knockdown on migration and invasion in A549 (**H**) and H157 cells (**I**) using transwell assays and ImageJ was used to perform quantitative analysis (*n* = 4 in each group). Scale bar, 100 μm. **J** Schematic showing that HNRNPC regulates Sonic Hedgehog signaling and NSCLC progression by HNF1A. Data in (**A**–**D**, **F**–**I**) are presented as the mean ± SD. Statistical significance was assessed by a one-way ANOVA (**A**–**D**, **H**, **I**) and a two-way ANOVA (**F**, **G**). *P < 0.05, **P < 0.01, ***P < 0.001. Experiments (**A**–**I**) were repeated at least three times.

We further examined the effects of HNF1A on HNRNPC-mediated Sonic Hedgehog signaling and tumor progression. Our results showed that silencing of HNF1A impaired the HNRNPC-mediated increase of GLI luciferase activity and mRNA upregulation of Hedgehog signaling-associated markers (Fig. 7A–D). Meanwhile, silencing of HNF1A blocked the protein level increase of SHH, GLI1, c-Myc, and N-cadherin or the decrease of E-cadherin in HNRNPC-overexpression cells (Fig. 7E). Moreover, the knockdown of HNF1A inhibited the cell proliferation, migration, and invasion induced by HNRNPC overexpression (Fig. 7F–I). These data reveal that HNRNPC positively regulated Sonic Hedgehog signaling and promoted NSCLC progression through HNF1A (Fig. 7J). Altogether, HHIPL2 positively regulates Hedgehog signaling and promotes NSCLC progression via the HNRNPC-HNF1A axis.

### Triptolide impedes HHIPL2-mediated proliferation and metastasis via its targeted inhibition of HNF1A expression

**Fig. 8:**
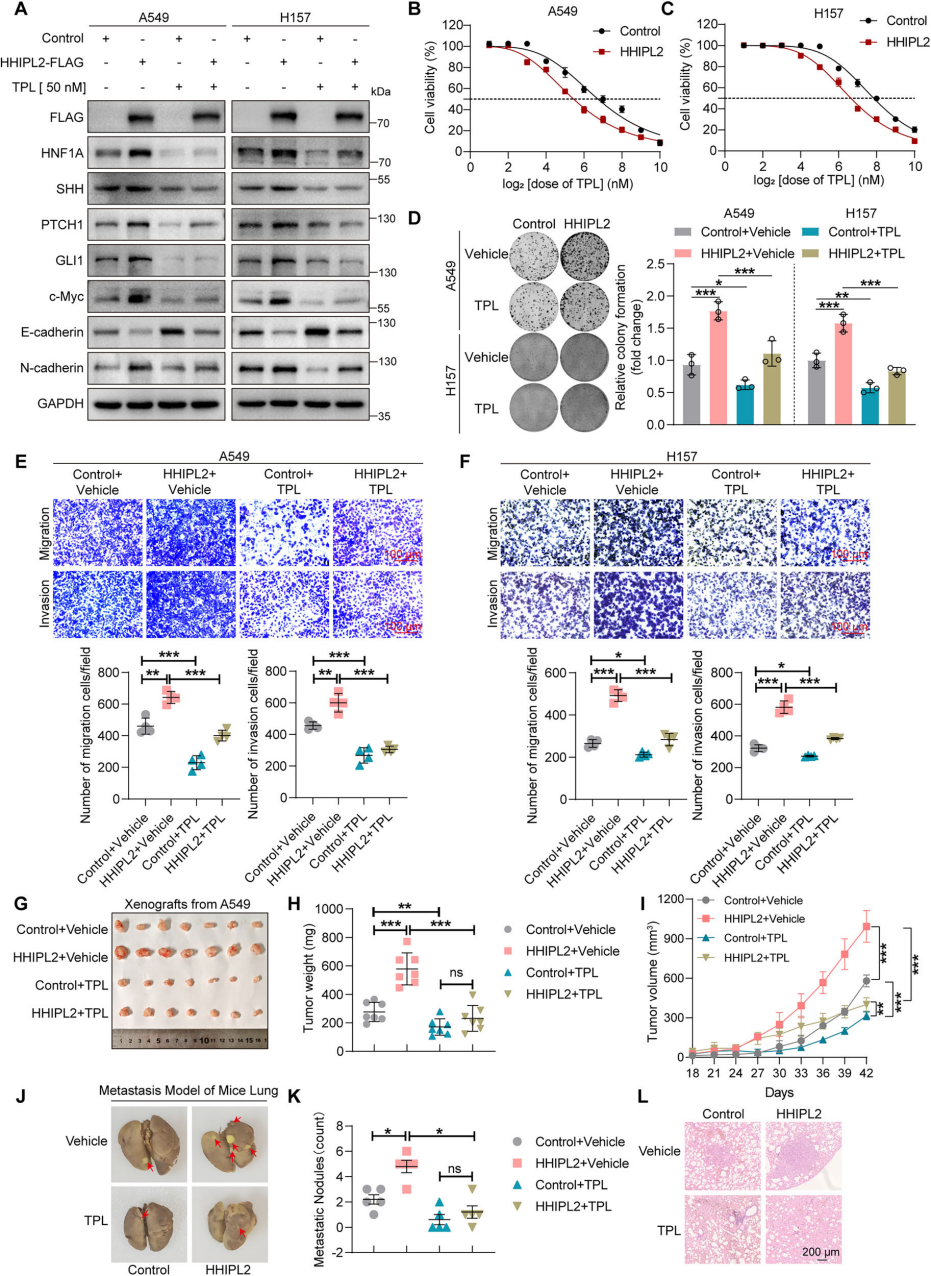
Triptolide impedes HHIPL2-mediated proliferation and metastasis via its targeted inhibition of HNF1A expression. **A** Overexpression of HHIPL2 in A549 and H157 cells in the presence or absence of triptolide (TPL) (50 nM) for 24 h. Cell lysates were analyzed by Western blot. **B**, **C** Cell viability was detected by the CCK-8 method, and the dose-response curves of triptolide (TPL) in HHIPL2-overexpression and control cells after 24 h of TPL (0, 2, 4, 8, 16, 32, 64, 128, 256, 512, and 1024 nM) treatment were plotted. The IC_50_ value was calculated according to the fitting curves. **D** Colony formation assays of A549 and H157 cells overexpressing HHIPL2 after treatment with DMSO and triptolide (TPL) (50 nM). ImageJ was used to perform quantitative analysis (*n* = 3 in each group). Effects of HHIPL2 overexpression in the presence or absence of triptolide (TPL) (50 nM) on migration and invasion in A549 (**E**) and H157 cells (**F**) using transwell assays, and ImageJ was used to perform quantitative analysis (*n* = 4 in each group). Scale bar, 100 μm. BALB/c nude mice were injected subcutaneously with stably transduced A549-Control and A549-HHIPL2 cells. After 27 days, triptolide (TPL) (0.1 mg/kg) was intraperitoneally injected at intervals of 2 days. After 42 days, the mice were then euthanized. The transplanted tumors were removed and photographed (**G**), Tumors were isolated, and the weight (**H**) and volumes (**I**) were measured (*n* = 7 per group). Representative images of lung metastasis models in nude mice after tail injection of A549 cells with HHIPL2 overexpression and triptolide (TPL) (0.1 mg/kg) treatment (**J**) and quantification of pulmonary metastatic nodules (**K**) (*n* = 5 per group). Representative H&E staining in lung sections (**L**). Data in (**D**–**F**, **H**, **I**, **K**) are presented as the mean ± SD. Statistical significance was assessed by a one-way ANOVA (**D**–**F**, **H**, **K**) and a two-way ANOVA (**I**). *P < 0.05, **P < 0.01, ***P < 0.001. Experiments (**A**–**F**) were repeated at least three times.

Triptolide is a diterpenoid epoxide isolated from the traditional Chinese medicinal herb *Tripterygium wilfordii* Hook F. It has been reported that triptolide exerts an anti-tumor effect [37]. Our previous study found that triptolide directly targeted and inhibited HNF1A to impede Sonic Hedgehog signaling activation [36]. To further assess the role of triptolide in HHIPL2-mediated Sonic Hedgehog signaling and progression in NSCLC, we examined the effect of triptolide in the HHIPL2-overexpression cells. As shown in Fig. S10A, B, triptolide repressed HHIPL2-induced mRNA upregulation of Sonic Hedgehog signaling markers. We also found that triptolide treatment blocked HHIPL2 overexpression-induced changes in the expression of proteins, which were related to Sonic Hedgehog signaling, proliferation, and metastasis (Fig. 8A). To further assess the role of triptolide in HHIPL2-mediated progression in NSCLC, we first determined Half Maximal Inhibitory Concentration (IC_50_) values of triptolide. The results showed that IC_50_ values of triptolide dramatically reduced in the HHIPL2-overexpression cells, suggesting that HHIPL2 increased cells’ sensitivity to triptolide (Fig. 8B, C). Meanwhile, triptolide significantly suppressed cell proliferation induced by HHIPL2 overexpression (Fig. 8D). In addition, increased migration and invasion capacity induced by HHIPL2 overexpression could be impaired by triptolide treatment (Figs. 8E, F), which was consistent with western blot analyses (Fig. 8A). To further verify the effect of triptolide in HHIPL2-regulated cell proliferation in vivo, A549 cells overexpressing HHIPL2 were subcutaneously injected into BALB/c nude mice to generate xenograft models. Following treatment with triptolide, we measured the tumor weight and growth rate. We found that triptolide treatment dramatically inhibited HHIPL2-induced tumor growth, as evidenced by the reduced tumor weight and growth rate (Fig. 8G–I). Immunohistochemistry analysis showed that the triptolide reversed the expression increase of Ki67, HNF1A, and SHH induced by overexpressing HHIPL2 (Fig. S10C). Moreover, triptolide treatment abolished the accelerated role of HHIPL2 in tumor metastasis, as supported by the decreased number of lung metastasis nodes (Fig. 8J–L). Altogether, these results demonstrate that triptolide, as an inhibitor against HNF1A expression, can effectively overcome HHIPL2-induced NSCLC progression.

## Discussion

NSCLC is a common malignant tumor with a poor prognosis. However, exploring key targets that drive its progression is a vital challenge in NSCLC therapy [38]. Aberrant activation of Hedgehog signaling can result in tumorigenesis in multiple human cancers, including NSCLC [39]. Hedgehog signaling regulates the proliferation, metastasis, and drug resistance of NSCLC [36, 40, 41]. The HHIP family consists of HHIP, HHIPL1, and HHIPL2. HHIP is known to negatively regulate Hedgehog signaling [14]. In addition, HHIPL1 is a secreted protein that interacts with SHH and increases Hedgehog signaling activity [20]. However, it is unknown whether HHIPL2 regulates the Hedgehog signaling pathway. In the present study, we first proposed that HHIPL2 plays a vital role in regulating Sonic Hedgehog signaling in NSCLC. Interestingly, HHIPL2 exhibited stronger positive effects compared to HHIPL1. We showed that HHIPL2 promotes NSCLC proliferation and metastasis by positively regulating Sonic Hedgehog signaling.

In the previous study, the *HHIPL2* gene showed a higher copy number in gastric cancers than in normal tissues [22]. In LUSC, the expression of HHIPL2 is considered to be correlated with immune cells and drug response [21]. So far, however, the molecular mechanisms responsible for HHIPL2 are poorly understood in cancers. In this study, we observed that HHIPL2 expression was elevated in NSCLC compared with normal tissues, and high HHIPL2 levels correlated with poor prognosis. Furthermore, bioinformatics analysis using the cBioPortal database revealed significantly elevated HHIPL2 expression levels in lung adenocarcinoma compared to lung squamous cell carcinoma. Notably, HHIPL2 exhibited a stronger association with adverse prognosis, nodal status, and tumor stages in lung adenocarcinoma patients relative to both lung squamous cell carcinoma and broader lung carcinoma cohorts, a correlation potentially attributable to its comparatively higher expression in lung adenocarcinoma. Moreover, our study found that HHIPL2 promoted proliferation and metastasis in NSCLC. Meanwhile, we found that higher expression of HHIPL2 is widespread in a pan-cancer manner. Mechanistically, we observed that HHIPL2 positively regulated Sonic Hedgehog signaling by increasing the Hedgehog luciferase activity and upregulating the mRNA expression of its essential molecules, such as SHH, PTCH1, and GLI1, further promoting NSCLC progression. In addition, we found that the protein expression of HHIPL2 was correlated positively with SHH, the upstream initiator of Sonic Hedgehog signaling [42], in NSCLC tumor tissues. Inhibition of Sonic Hedgehog signaling abolished the proliferation, migration, and invasion caused by HHIPL2 overexpression in NSCLC cells. Overall, our work found that HHIPL2, in contrast to HHIP, promotes NSCLC progression by positively regulating Sonic Hedgehog signaling.

HNF1A is a transcription factor that binds to multiple target genes’ promoter regions to elicit various biological functions related to lipoprotein metabolism, bile acids, cholesterol, and cancers [43–45]. In one recent study, we found that HNF1A was a novel transcription factor of SHH that positively regulates the Sonic Hedgehog signaling [36]. In the current study, we found that HHIPL2 affects the mRNA expression of HNF1A. Moreover, we showed that HHIPL2 positively regulated SHH-mediated Sonic Hedgehog signaling and NSCLC progression through HNF1A. In addition, the protein expression of HHIPL2 was positively correlated with HNF1A through the analysis of NSCLC tumor tissues, indicating that HNF1A accelerated HHIPL2-mediated Sonic Hedgehog signaling and NSCLC progression. We previously identified triptolide as an inhibitor of HNF1A that impairs Sonic Hedgehog signaling. Here, we explored the role of triptolide in HHIPL2-mediated NSCLC proliferation and metastasis. This current study showed that triptolide dramatically inhibited tumor growth and metastasis caused by HHIPL2 overexpression in NSCLC cells and xenograft models. Our work strongly indicates the therapeutic potential benefits of this traditional Chinese medicine monomer in HHIPL2-high expression NSCLC patients.

The altered mRNA levels are involved in gene transcription and mRNA stabilization [46]. However, HHIPL2 is almost impossible to enforce these regulations to affect HNF1A mRNA directly due to its lack of domains with relevant functions. Hence, we tried to look for potential partners binding to HHIPL2, which could reveal the molecular mechanism underlying HHIPL2-upregulated HNF1A mRNA. Interestingly, we found that HNRNPC, an RNA-binding protein, interacted with HHIPL2. Moreover, HNRNPC was found to interact with HNF1A mRNA and regulate its stability. More specifically, our mass spectrometry analysis and Co-IPs assays in the present study showed that HNRNPC interacted with HHIPL2. We used the AlphaFold Protein Structure Database [47] to obtain the protein structure of HHIPL2 and HNRNPC and subsequently conducted molecular docking analysis. Combined with the structural analysis and our Co-IP assays, we inferred that HNRNPC interacted with HHIPL2 through the non-RRM domain. Notably, we also observed that HHIPL2 did not affect the protein expression of HNRNPC but interfered with its cell localization. HHIPL2 increased the cytoplasmic localization of HNRNPC and decreased its accumulation in the nucleus. However, the specific molecular mechanisms of HHIPL2 affecting HNRNPC nucleo-cytoplasmic translocalization need further investigation. As an RBP, HNRNPC is well known for its regulatory roles in translation, sequence-unspecific RNA exportation, RNA splicing, and RNA stability [48–50]. In this study, we found that HNRNPC interacted with HNF1A mRNA and boosted its stability. HHIPL2 promotes the interaction between HNRNPC and HNF1A mRNA. Furthermore, HNRNPC positively regulated the HNF1A-dependent Sonic Hedgehog signaling. In ESCC, HNRNPC has been reported to positively regulate Hedgehog signaling by promoting GLI2 mRNA stability [27]. In NSCLC, we found a new mechanism by which HNRNPC regulates the Sonic Hedgehog signaling pathway.

Previously, multiple studies have shown that HNRNPC acts as an oncogene to promote tumorigenesis and progression of several cancers, including NSCLC [51–54]. However, the detailed function of HNRNPC in NSCLC is still unclear. This study found that HNRNPC knockdown abolished Sonic Hedgehog signaling activation and NSCLC progression caused by HHIPL2 overexpression. In addition, HNF1A knockdown also impaired the HNRNPC-induced Sonic Hedgehog signaling activity and NSCLC progression. All these results suggest that HNRNPC promoted the HHIPL2-mediated Sonic Hedgehog signaling and NSCLC progression in an HNF1A/SHH axis-dependent manner.

In summary, we elucidated that HHIPL2, as an oncogene in NSCLC, positively regulates Sonic Hedgehog signaling and promotes NSCLC proliferation and metastasis by the HNRNPC-HNF1A-SHH axis. Thus, based on our present findings, we propose that combination treatment with Sonic Hedgehog signaling inhibitors and potential anticancer agents targeting HHIPL2 may present an effective strategy for NSCLC treatment.

## Materials and methods

### Cell lines and cell culture

The HEK293T, A549, H1975, BEAS-2B, and H460 cells were purchased from Shanghai Fuheng Biological (Shanghai, China). H157 and H1792 cells were kindly provided by Dr. Xiangguo Liu. Short tandem repeat (STR) was used to authenticate all cell lines. HEK293T cells were cultured in Dulbecco’s modified Eagle’s medium (DMEM, Corning, USA) supplemented with 10% (V/V) fetal bovine serum (FBS, ExCell Bio, China). H1975, BEAS-2B, H460, H157, and H1792 cells were cultured in RPMI-1640 (Corning, USA) supplemented with 10% FBS. A549 cells were cultured in Ham’s F12-K (BasalMedia, China) supplemented with 10% FBS. All cell lines were maintained at 37 °C in a humidified atmosphere of 5% CO2 and tested to ensure no contamination from mycoplasma.

### Reagents and antibodies

The chemicals used in our experiments were Triptolide (HY-32735), Cyclopamine (HY-17024), and Actinomycin D (HY-17559), which were purchased from MedChemExpress. Puromycin Dihydrochloride (ST551) and Hygromycin B (ST1389) were purchased from Beyotime. The primary antibodies used in the western blot and immunoprecipitation assays are listed in Supplementary Table 2.

### Plasmids and siRNA transfection

The human HHIP-FLAG, HHIPL1-FLAG, HHIPL2-FLAG, HHIPL2-GFP, HHIPL2-MYC, HNRNPC-FLAG, and HNRNPC mutations (RRM, ΔRRM, NLS-deletion) coding regions were subcloned into the PLVX-puro vector. All siRNAs and shRNAs were synthesized by Tsingke Biotechnology. Detailed information on the siRNAs and shRNAs is listed in Supplementary Table 3. For transient transfection, cells were transfected with plasmids or siRNA using LipoMax^TM^ reagent (32012, Sudgen Biotechnology) or siTran2.0^TM^ siRNA transfection reagent (TT320002, Origene) in serum-free Opti-MEM (Gibco) according to the instruction manual.

### Reverse transcription quantitative polymerase chain reaction (RT-qPCR)

Total RNA was extracted from cells or fresh tissues with Trizol reagents (TIANGEN) following the manufacturer’s instructions. Reverse transcription was performed using the lnRcute lncRNA First-Strand cDNA Kit (KR202, TIANGEN). Real-time quantitative PCR analysis of cDNA was performed using the SYBR Green Fluorescence Quantification Kit (TSE201, Tsingke). qPCR was performed using a QuantStudio^TM^ 5 Real-time PCR System (Thermo Scientific). The comparative cycle threshold method (△△Ct) was used to quantify the relative expression of each gene. The samples were loaded in triplicate, and the results of each sample were normalized to GAPDH. The primers used in the present study are listed in Supplementary Table 4.

### Western blot analysis and co-immunoprecipitation (Co-IP)

The cells were lysed in RIPA lysis buffer (WB3100, New Cell and Molecular) on ice for 10 min and then purified via centrifugation for 10 min at 4 °C. Equivalent protein quantities were subjected to 8–15% SDS-PAGE, transferred to PVDF membranes, and probed with primary antibodies, followed by the relevant peroxidase-conjugated anti-mouse secondary antibodies (ZB2305, ZSGB-BIO) or anti-rabbit secondary antibodies (ZB2301, ZSGB-BIO). Immunoreactive bands were visualized using a chemiluminescence kit (WBKLS0500, Millipore) with a Tanon Imaging System (Tanon, China). The full and uncropped images for Western blots were uploaded to the Supplementary file. The bands from the three replicate experiments were quantified using ImageJ software [55].

For Co-IP, briefly, cells were lysed in IP lysis buffer for 30 min and then purified via centrifugation for 10 min at 4 °C. Anti-FLAG magnetic beads (HY-K0207, MedChemExpress) were incubated with supernatant for 4 h at 4 °C, or the lysates were immunoprecipitated with the specific antibody and protein A/G magnetic beads (HY-K0202, MedChemExpress) for 4 h. The beads were washed with 1 mL lysis buffer five times and eluted in 50 μL of 2× SDS loading buffer. Then, samples were analyzed using the Western blot.

### Identification of proteins interacting with HHIPL2 by LC-MS/MS

A549 cells were transfected with HHIPL2-FLAG plasmids for 48 h, and anti-FLAG magnetic beads were used for Co-IP assays. Then, the identification and enrichment of proteins interacting with HHIPL2 by LC-MS/MS analyses were conducted and supported by the Novogene Company.

### RNA stability assessment

Cells were transfected with siRNA and then treated with Actinomycin D (2 μg/mL) for the indicated durations (0, 1, 2, and 3 h). Samples collected with Trizol were extracted to total mRNA and subjected to RT-qPCR to evaluate HNF1A mRNA degradation. The samples were loaded in triplicate, and the results of each sample were normalized to GAPDH. Experiments were repeated at least three times.

### RNA immunoprecipitation (RIP) assay and RNA pull-down assay

RNA immunoprecipitation kit (P0102, Geneseed, China) was used for RIP and RNA pull-down assays. For RIP assays, anti-FLAG magnetic beads were incubated with supernatant for 6 h at 4 °C. Afterward, it was washed with 1 mL of RIP lysis buffer five times. Then, half of the beads were resuspended in the 2× SDS loading buffer using the western blot. The other half of the beads were resuspended in Trizol to extract RNA and subjected to RT-PCR. For RNA pull-down assays, the streptavidin magnetic beads (HY-K0208, MedChemExpress) were first mixed with 100 pmol of HNF1A probe and incubated for 2 h at 4 °C. Then, the RNA-protein mixture was added to the beads and incubated for 6 h at 4 °C. Lastly, the western blot resuspended half of the beads in the 2× SDS loading buffer. The other half of the beads were resuspended in Trizol to extract RNA and subjected to RT-PCR. The sequence of the HNF1A probe: 5′-Biotin-ATTCCGCCCTATTGCACTCCTCCACTAGCGT-Cy3-3′.

### Protein-protein docking analysis

The fast Fourier transform-based docking algorithm ZDOCK 3.0.2 [56] was applied to predict the binding interface of HHIPL2 and HNRNPC. PDB files of HHIPL2 (AF-Q6UWX4-F1) and HNRNPC (AF-P07910-F1) were obtained from the AlphaFold Protein Structure Database [47] due to the incomplete experimental structure resolution. Intrinsically disordered regions (IDRs) domains were removed to elevate the rigid body docking performance. Then, receptor HHIPL2 (residues 1–642 aa) and ligand HNRNPC (residues 1–220 aa) were submitted to the ZDOCK server. Residue selection of the complex interface was skipped to avoid result manipulation. The pose with the highest ZDOCK score was chosen for further analysis. Energy minimization with 10,000 steps of steepest descent in the CHARMM36 force field was conducted using GROMACS [57] to remove side chain clashes. The refined pose was sent to UCSF Chimera X [58] to visualize and find hydrogen bonds.

### Immunofluorescence microscopy

Cells were fixed and permeabilized with 4% paraformaldehyde and 0.3% Triton X-100 for 10 min. Then, cells were blocked with 5% BSA and stained using primary antibodies overnight at 4 °C. After washing with PBS three times, the fluorescence-conjugated secondary antibodies (ab150078, Abcam; SA00013-1, Proteintech) were incubated at room temperature in the dark for 1 h, and an anti-fade DAPI solution was used for 10 min. After mounting, cells were visualized using a confocal microscope (63× oil, ZEISS LSM 800). Experiments were repeated at least three times.

### Cell counting kit-8 (CCK-8) assay and colony formation assay

Cells were seeded in 96-well culture plates at 3000, then cultured for 0, 24, 48, and 72 h. After treatment, the medium was removed, and 100 μL of CCK-8 mixture (90 μL of culture medium + 10 μL of CCK-8 reagent [C6005, New Cell and Molecular]) was added to each well and incubated for 1 h at 37 °C. The absorbance of each well was measured at 450 nm. All experiments were repeated at least three times.

For the colony formation assay, cells were seeded in 6-well plates at 1000 and continuously cultured for two weeks, after fixation with 4% paraformaldehyde for 10 min and staining with 0.1% crystal violet for 1 h, photographed, and scored. ImageJ was used to perform quantitative analysis. Experiments were repeated at least three times.

### Transwell migration assay and transwell invasion assay

The transwell assays were performed as previously described [59] to assess the migration and invasion capacity of NSCLC cells. Cells were visualized using an inverted microscope (20×, OLYMPUS). ImageJ was used to perform quantitative analysis. Cell migration and invasion values were expressed as the mean number of cells per microscopic field over four fields per insert for triplicate experiments. Experiments were repeated at least three times.

### Dual-luciferase reporter assay

Cells were seeded in 24-well plates. Then, 900 ng Gli-promoter-luciferase plasmids and 15 ng pRL-TK plasmids were co-transfected into cells, or 900 ng SHH-pGL3 (the SHH promoter sequence (from −499 to 101 bp) subcloned into pGL3-basic reporter gene vector) plasmids and 15 ng pRL-TK plasmids were co-transfected into cells. Luciferase activity was determined by a dual luciferase assay kit (RG029S, Beyotime) according to the manufacturer’s protocol. The Gli firefly luciferase/Renilla luciferase ratio was taken as a metric for Sonic Hedgehog signaling activity. The SHH promoter firefly luciferase/Renilla luciferase ratio was taken as a metric of SHH promoter activity. All experiments were repeated at least three times.

### Xenograft assay and metastasis assay in vivo

BALB/c nude mice (male, 4–5 weeks old) were purchased from Vital River Laboratories Animal Technology Co., Ltd (Beijing, China) and kept in a pathogen-free environment. The Second Hospital of Shandong University Animal Care Committee approved the experimental protocol, and all procedures complied with the institutional guidelines.

For xenograft assays, 5 × 10^6^ cells were injected subcutaneously into the mice. Mouse weight and tumor size were measured at intervals of 3 days. For triptolide administration experiments, after tumor inoculation, triptolide (0.1 mg/kg) was intraperitoneally injected every 3 days. Tumor volume was calculated using the formula π/6 × length × width^2^. The tumors were extracted and weighed when the maximum tumor volume approached 1 cm^3^. All tumors were excised and flash-frozen at −80 °C or formalin-fixed until further use.

For metastasis in vivo assays, a total of 1 × 10^6^ cells were injected through the tail vein of each mouse. After 12 weeks, the mice were euthanized to assess the metastasis of lung nodules. The lung tissues were collected, fixed with formalin, and embedded with paraffin for further analysis.

### Data mining

The HHIPL2 or transcription factors mRNA expression of normal or NSCLC tumor tissues, and the HHIPL2 mRNA expression in NSCLC on nodal metastasis status or individual cancer stages were obtained by the UALCAN database (https://ualcan.path.uab.edu/analysis.html) and the UCSC Xena database (https://xenabrowser.net/datapages/). The relationship between HHIPL2 or transcription factors mRNA expression and the overall survival of lung cancer patients was obtained from the Kaplan–Meier plotter database (https://kmplot.com/analysis/). The HHIPL2 mRNA expression in human cancer cell lines and non-cancerous cell lines was obtained from The Human Protein Atlas online database (https://www.proteinatlas.org/). The HHIPL2 mRNA expression in human cancers was obtained from the cBioPortal (https://www.cbioportal.org/). The data processing and mapping were carried out using R 4.5.1. The schematic diagram and the graphical abstract were created by Figdraw (https://www.figdraw.com/).

### Clinical sample

Tissue microarrays (TMAs) of human NSCLC clinical samples were created in the laboratory of Thoracic Cancer, Shandong University. TMAs involved matched normal tissues and tumor tissue samples from 55 patients. We also gathered another five pairs of fresh NSCLC clinical samples removed surgically for protein detection using Western blot. Written informed consent was obtained from all enrolled NSCLC patients. The postoperative stage was defined based on the tumor-node-metastasis (TNM) classification criteria for the eighth edition of the International Union Against Cancer (UICC). We acquired ethics approval from the Ethics Committee of the Second Hospital of Shandong University.

### Immunohistochemistry (IHC), tissue microarrays (TMAs) scoring, and hematoxylin and eosin (H&E) staining

Tumor xenografts were fixed in 4% paraformaldehyde and followed by paraffin embedding. The 4 μm thickness section was used for IHC and H&E staining. IHC and H&E staining were performed as previously described [60]. For TMAs scoring, the protein expression levels of HHIPL2, SHH, and HNF1A were scored semiquantitatively based on staining intensity and distribution using the immunoreactive score (IRS). IRS = SI (staining intensity) × PP (percentage of positive cells). SI: negative, 0; weak, 1; moderate, 2; strong, 3. PP: 0 = 0%; 1 = 1–25%; 2 = 26–50%; 3 = 51–75%; 4 = 76–100%. We chose a cutoff point for categorizing the continuous IRS values into high and low (range 0-12, IRS ≤ 4, low; IRS > 4, high). Clinicopathological correlations of HHIPL2, SHH, and HNF1A expression in NSCLC are listed in Supplementary Table 1.

### Statistics

All statistical data were analyzed using GraphPad Prism 9.5.1. Differences between the two groups were identified using a two-sided Student’s *t* test, and one-way ANOVA or two-way ANOVA was used for three or more groups. Spearman’s rank correlation coefficient analysis was used to assess the expression relationship among HHIPL2 and SHH or HNF1A in the NSCLC TMAs. Other significant differences were used in the Wilcoxon matched-pairs signed rank test and the Mann–Whitney test. All experiments for cell cultures were performed independently at least three times and in triplicate each time. In all cases, *P* < 0.05 was considered statistically significant. Statistical significance was also taken as *P < 0.05, **P < 0.01, and ***P < 0.001, ns, no significance.

## Supplementary information

Original Western Blots

Statistical Analysis of the Western Blot Bands

Supplementary Figure 1–10 and Table 2–4

Supplementary Table 1

Checklist

## Data Availability

All data are available in the main text or the supplementary materials.
