# Supplementary figures and images for: HHIPL2 positively governs Hedgehog signaling to accelerate non-small cell lung cancer progression via enhancing HNRNPC-mediated HNF1A mRNA stabilization
Source: Cell Death Dis. 2025 Dec 18;17(1):103. doi: 10.1038/s41419-025-08331-3 (PMC12848035; doi:10.1038/s41419-025-08331-3)

**Original western blots**


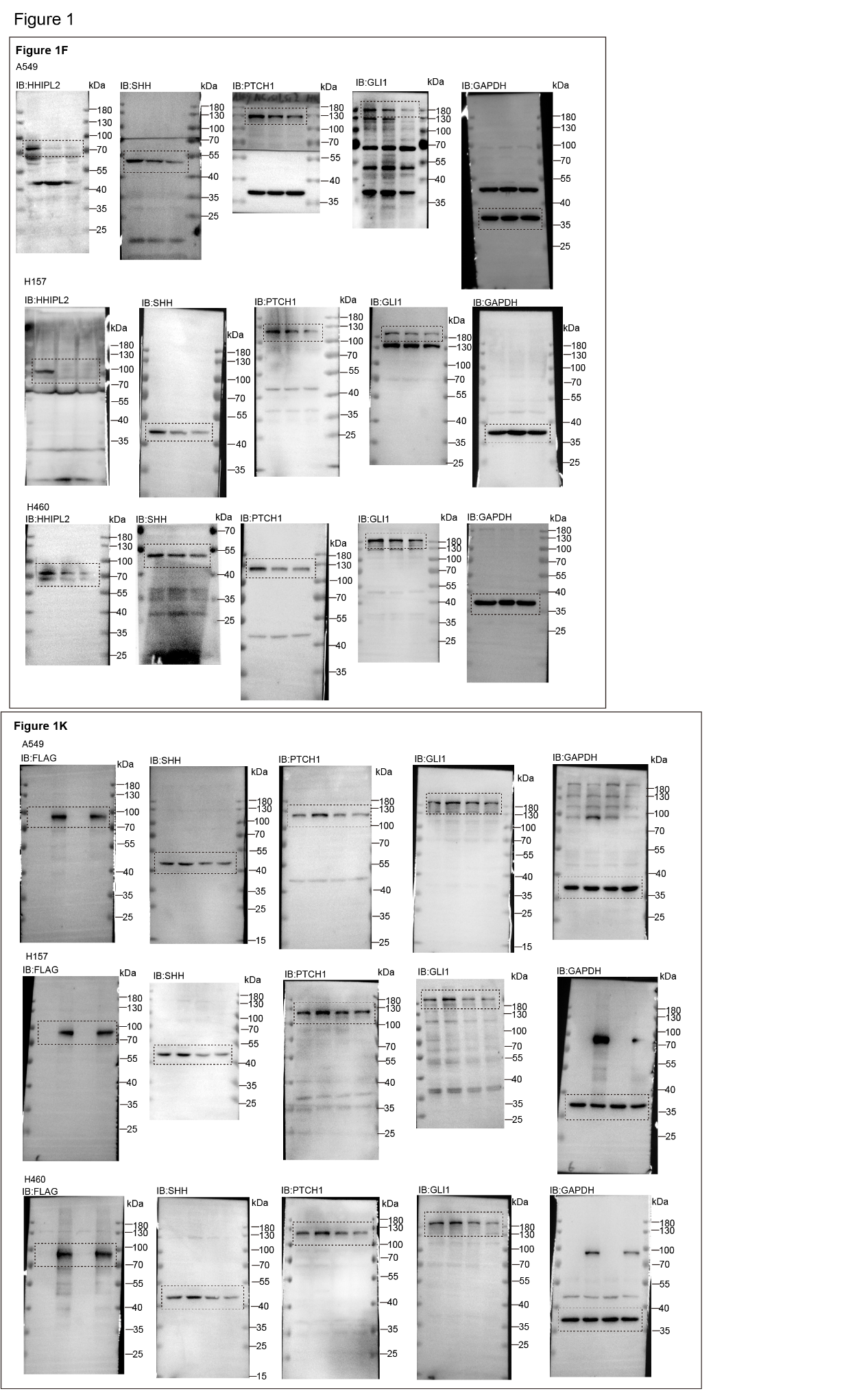


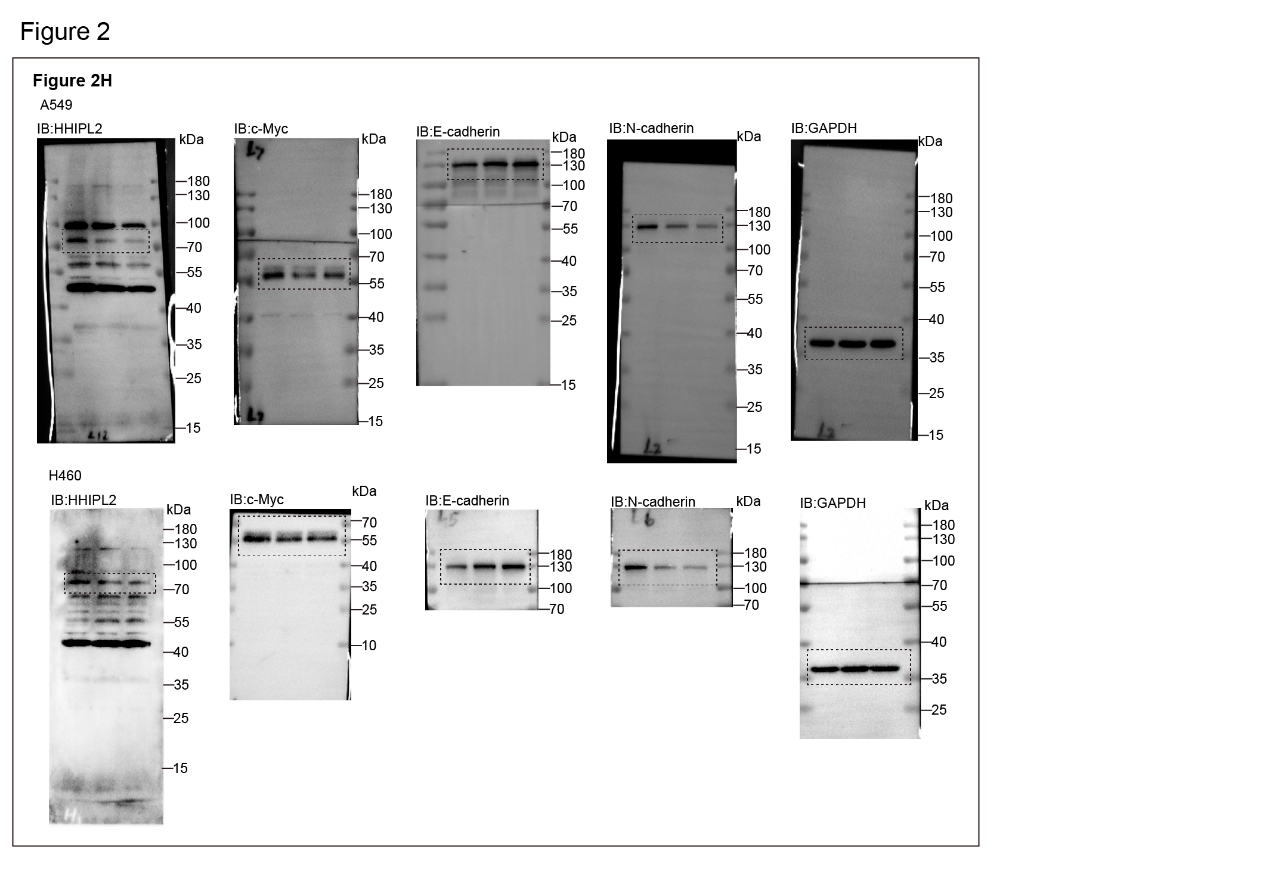


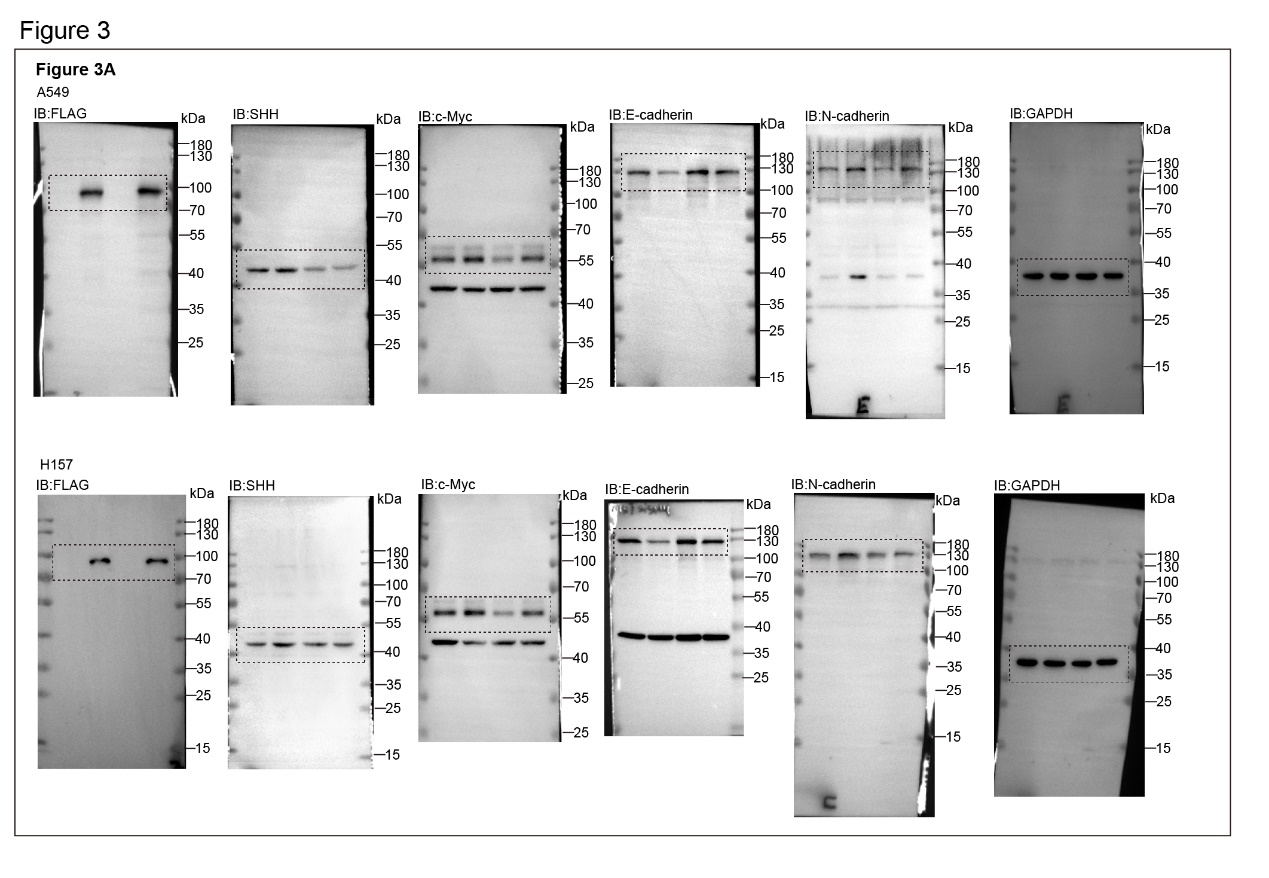


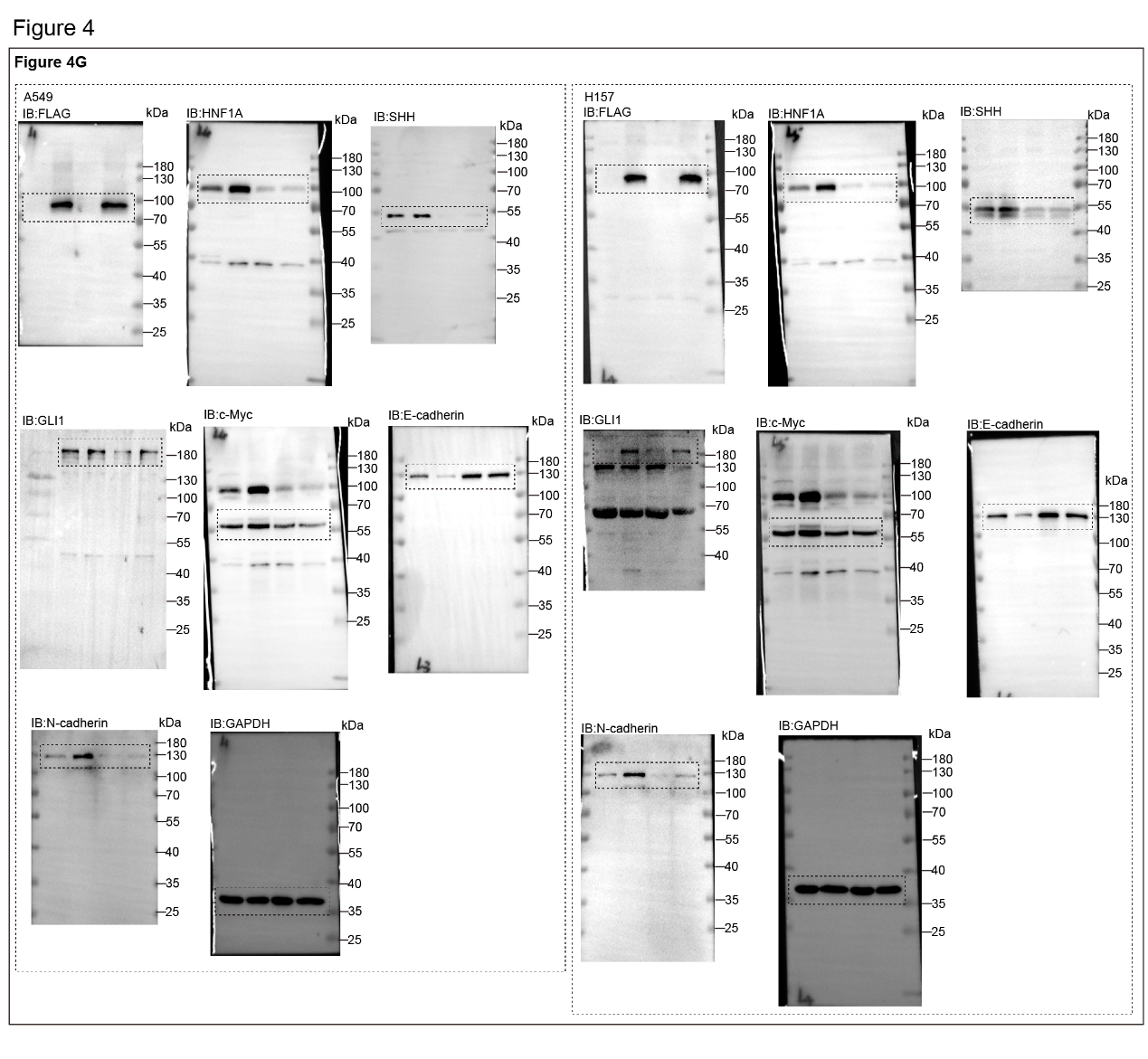


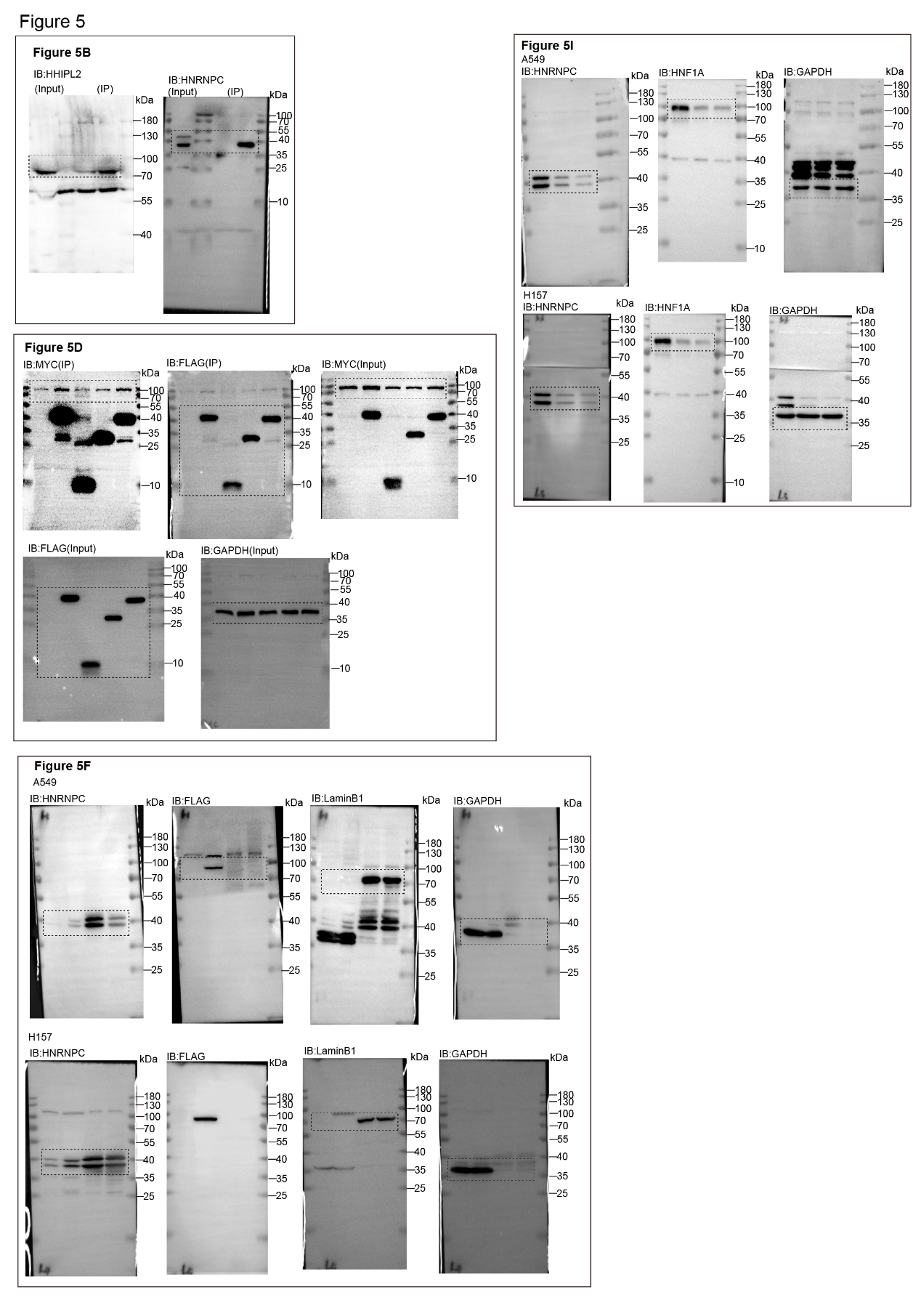


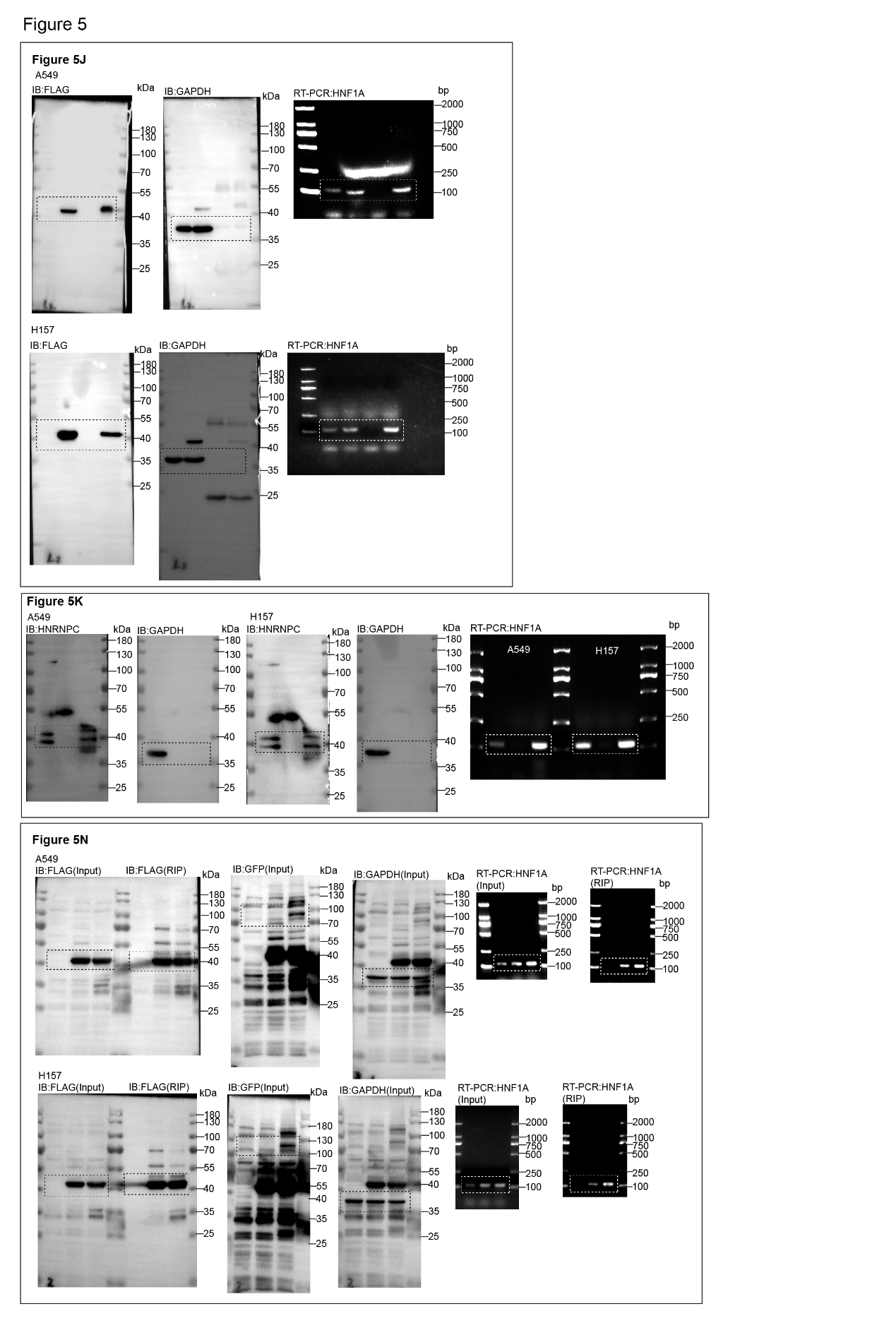


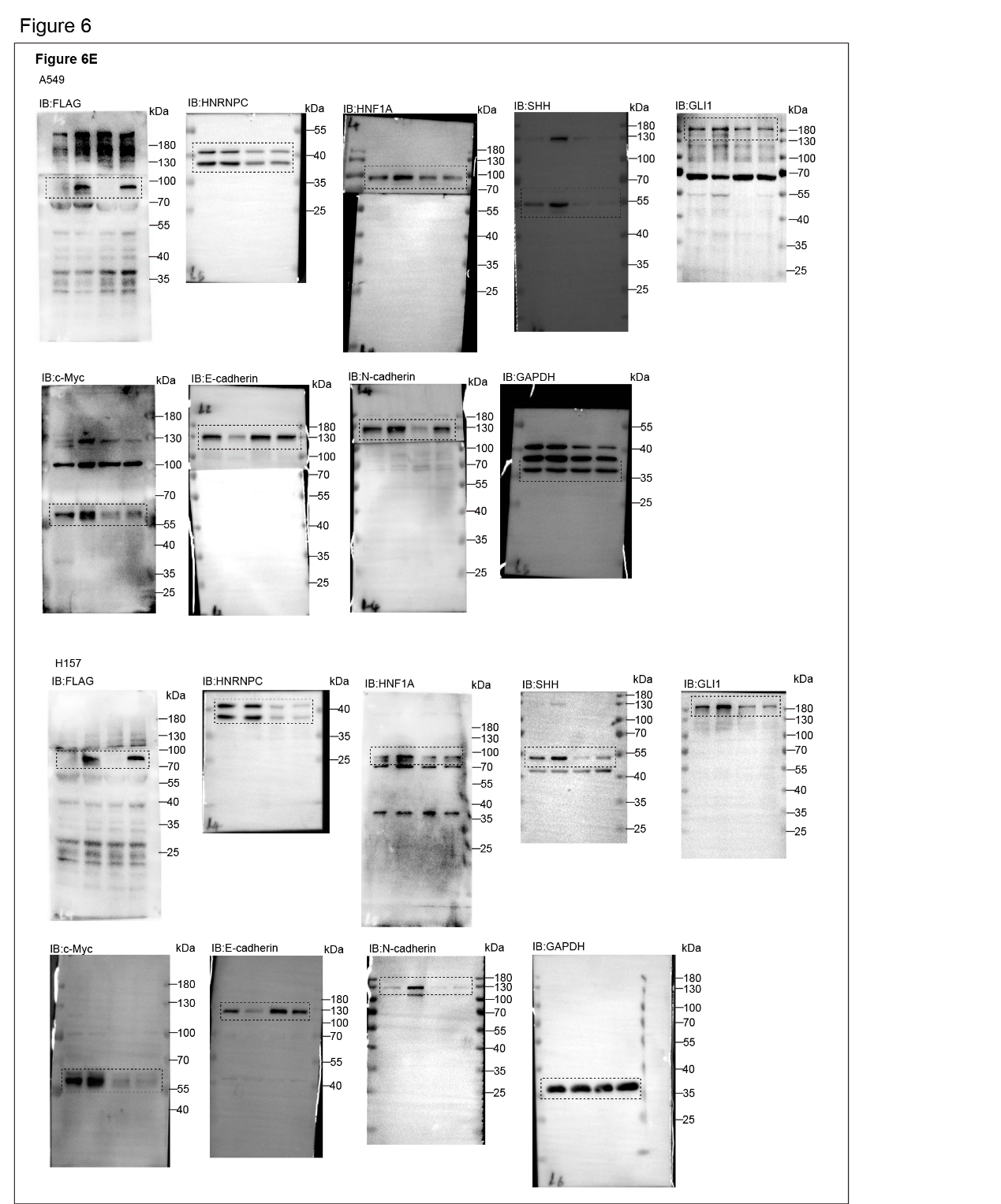


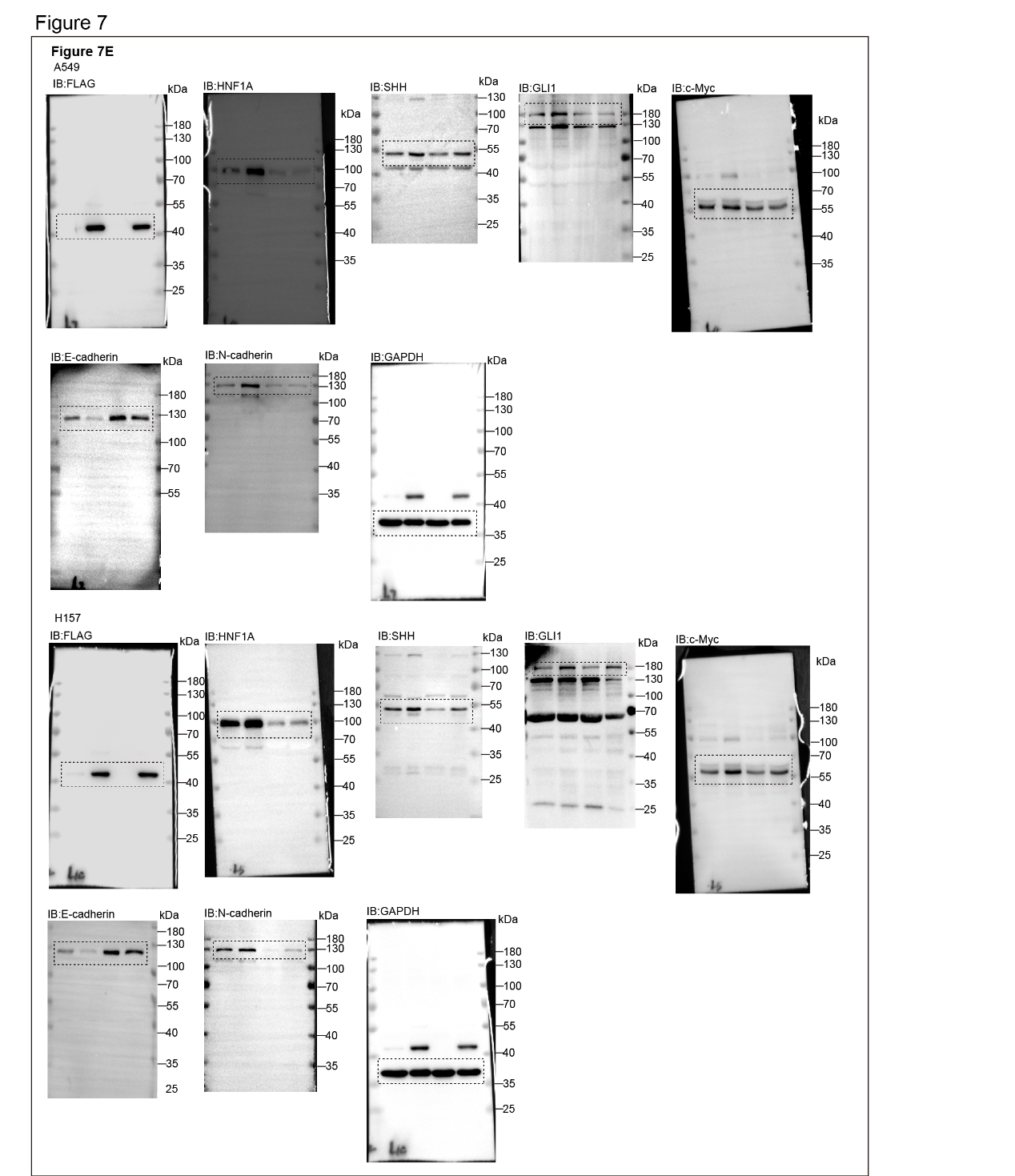


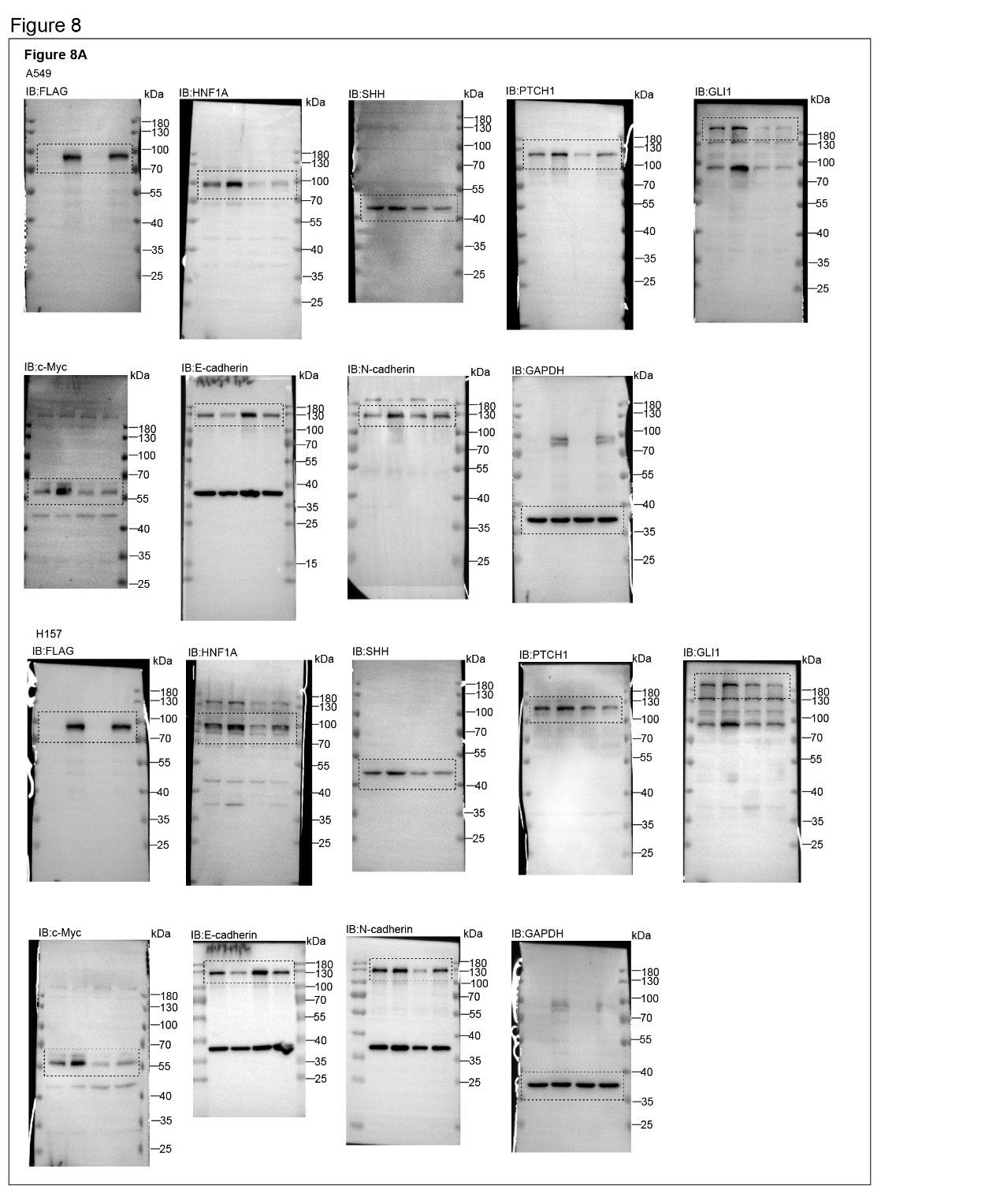


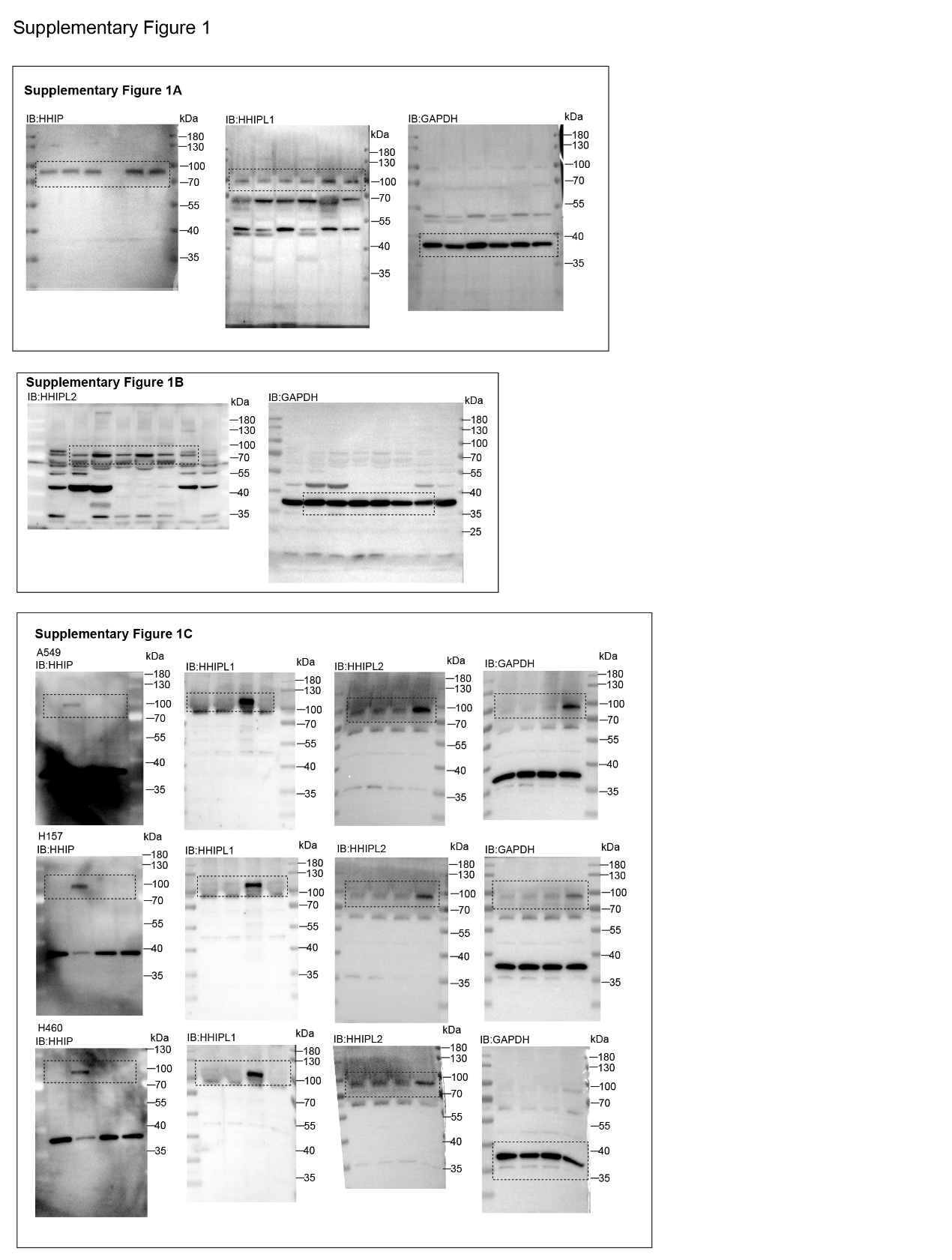


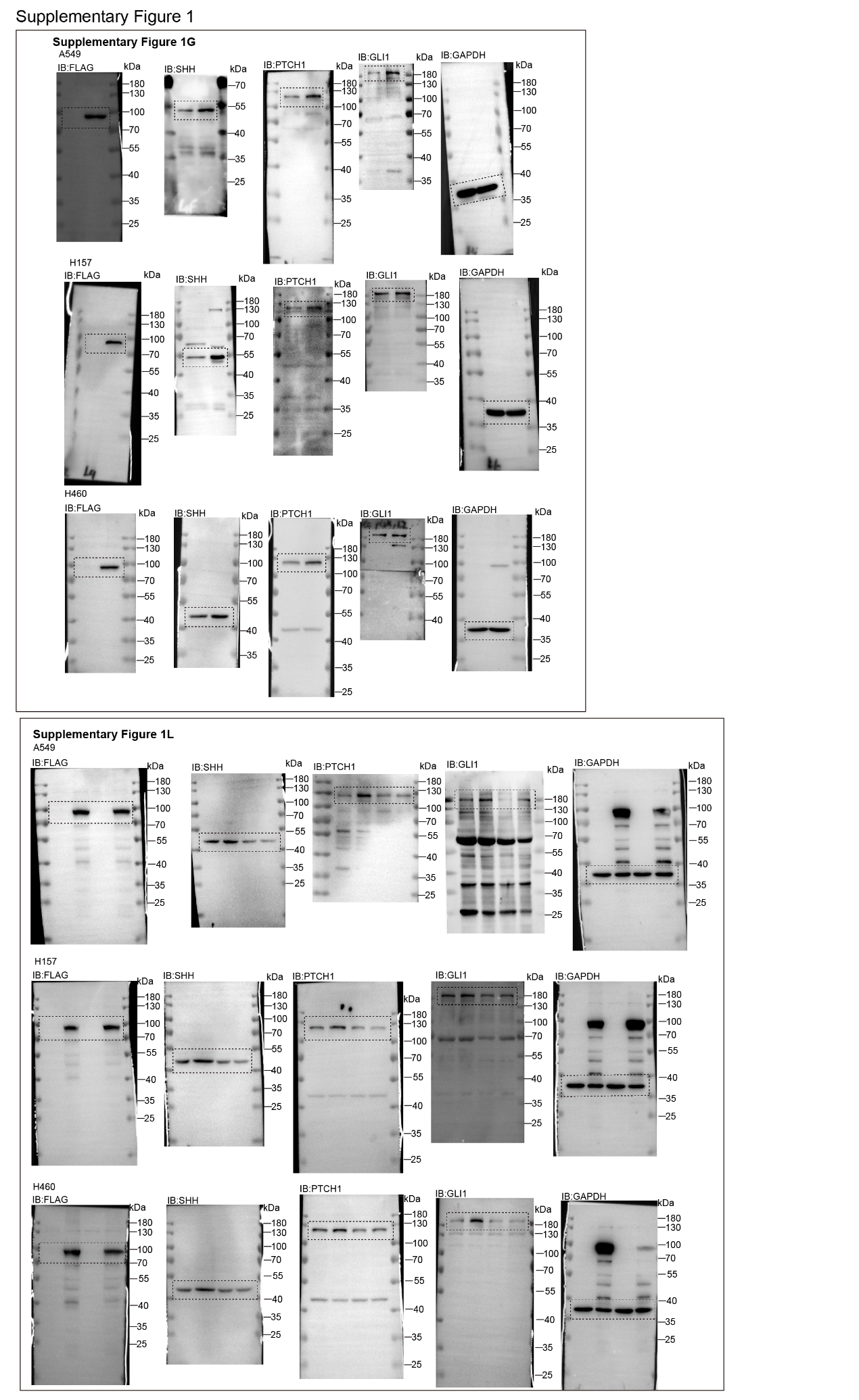


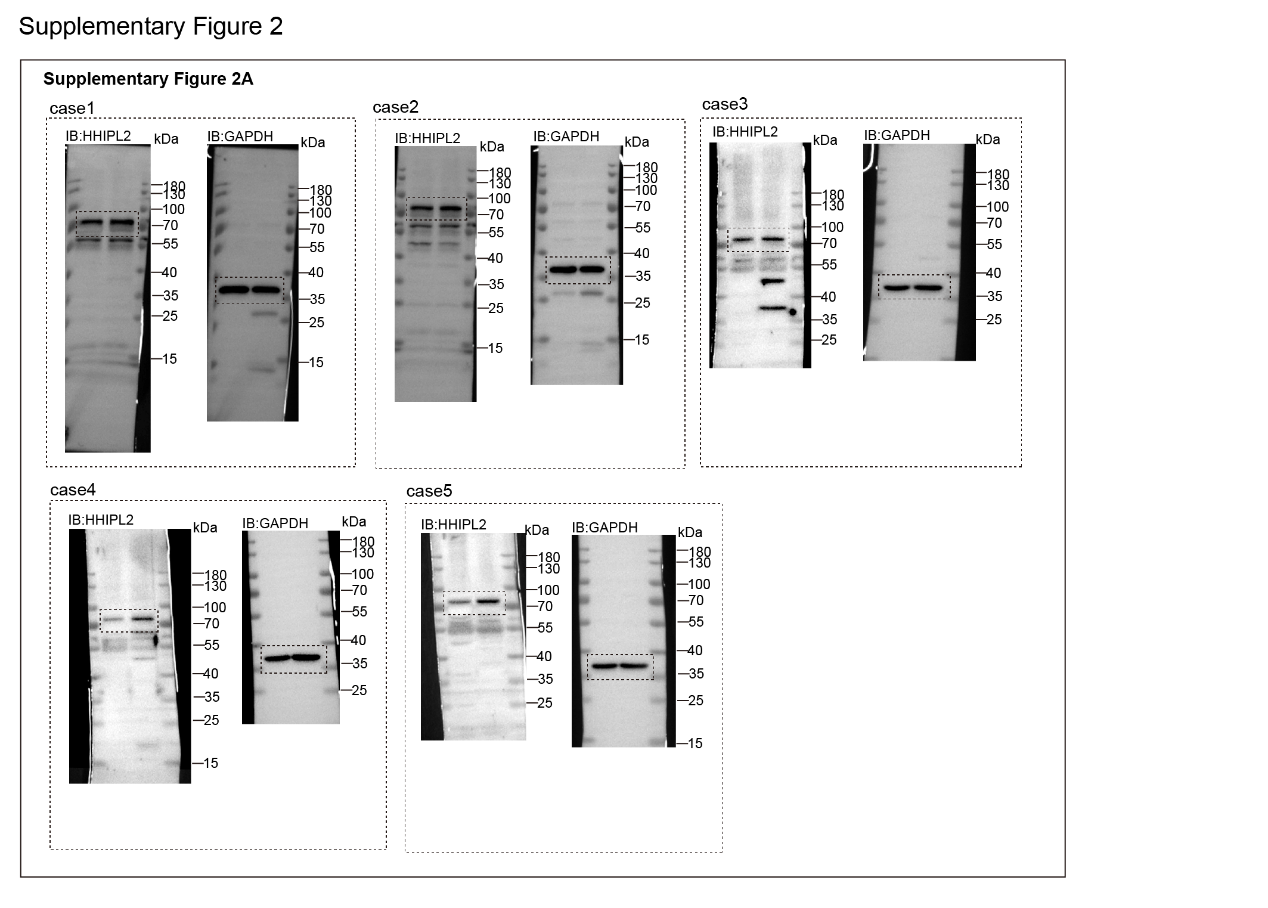


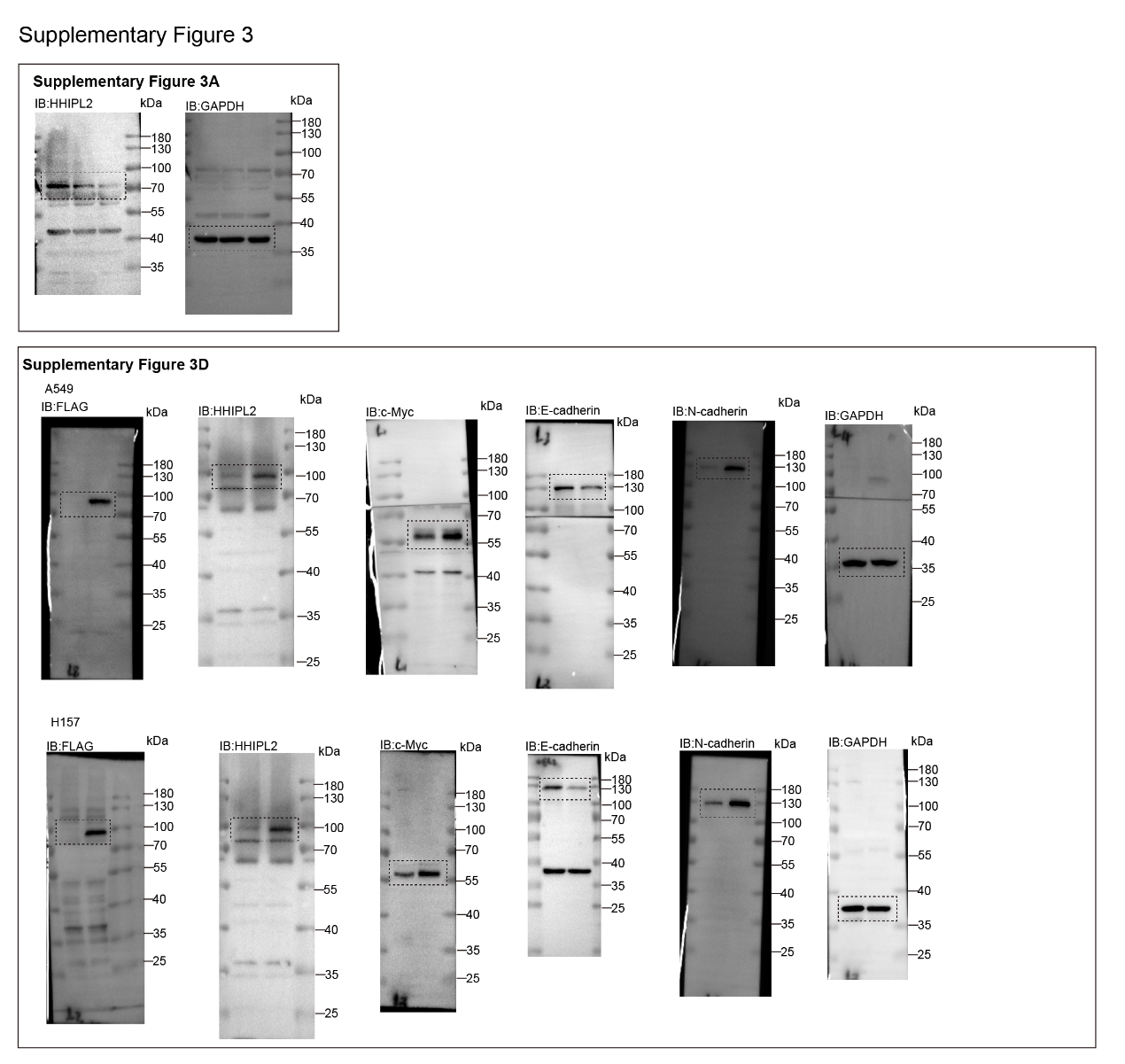


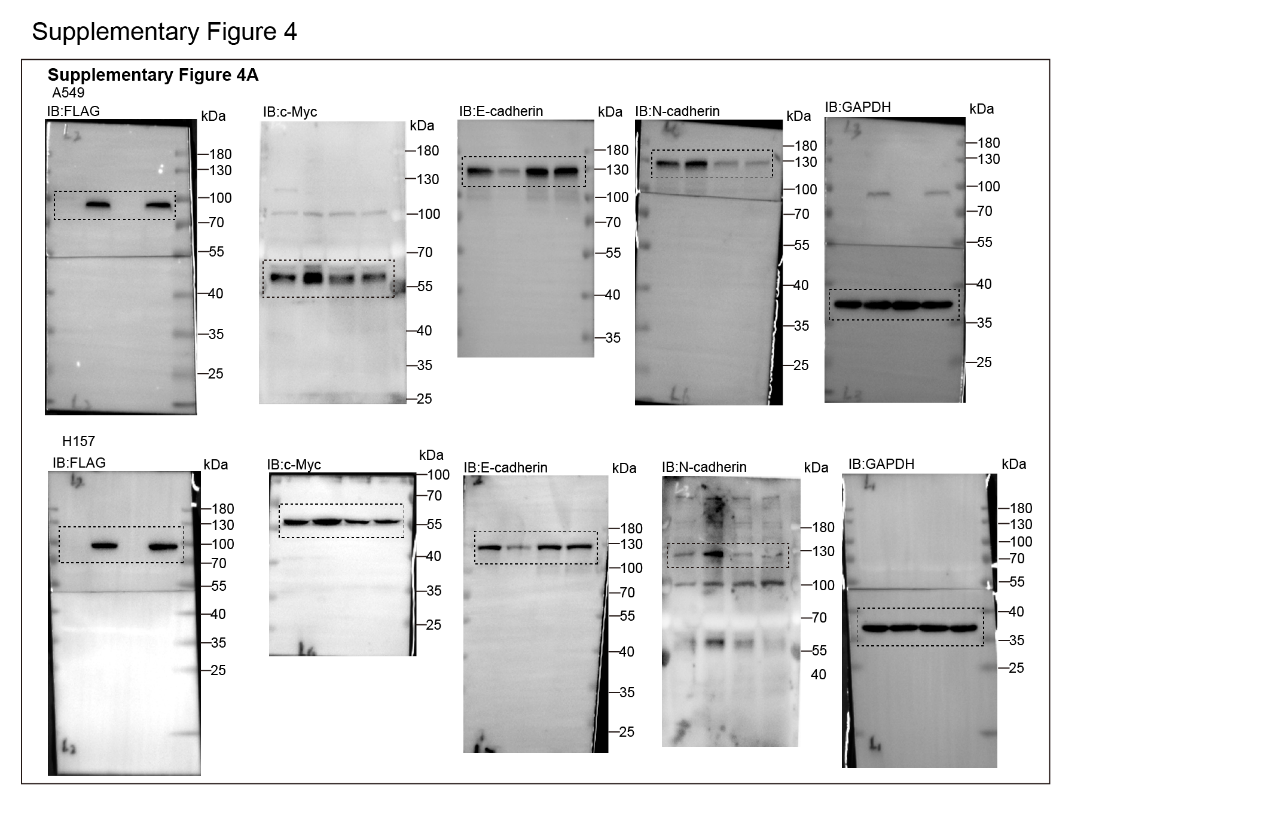


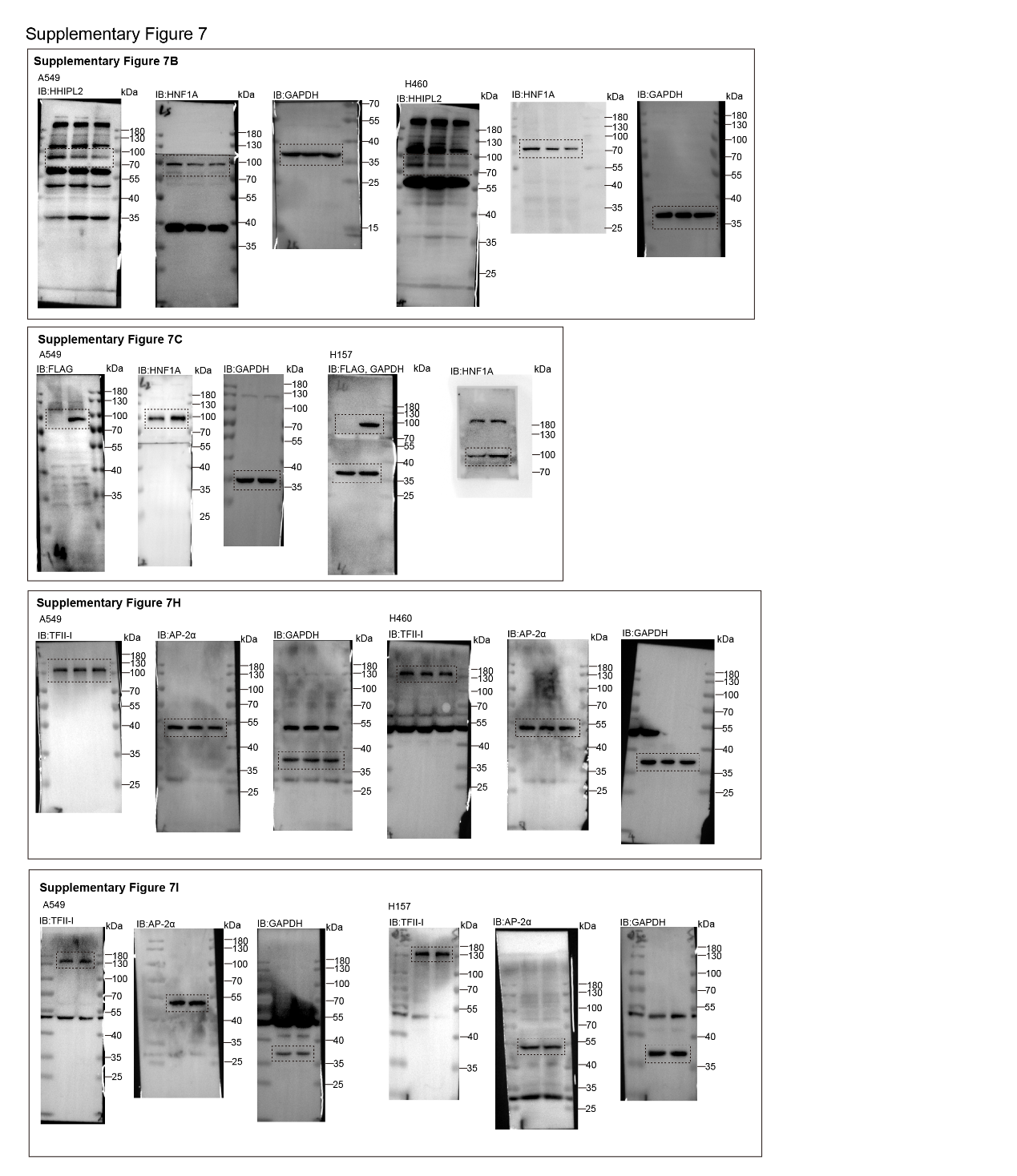


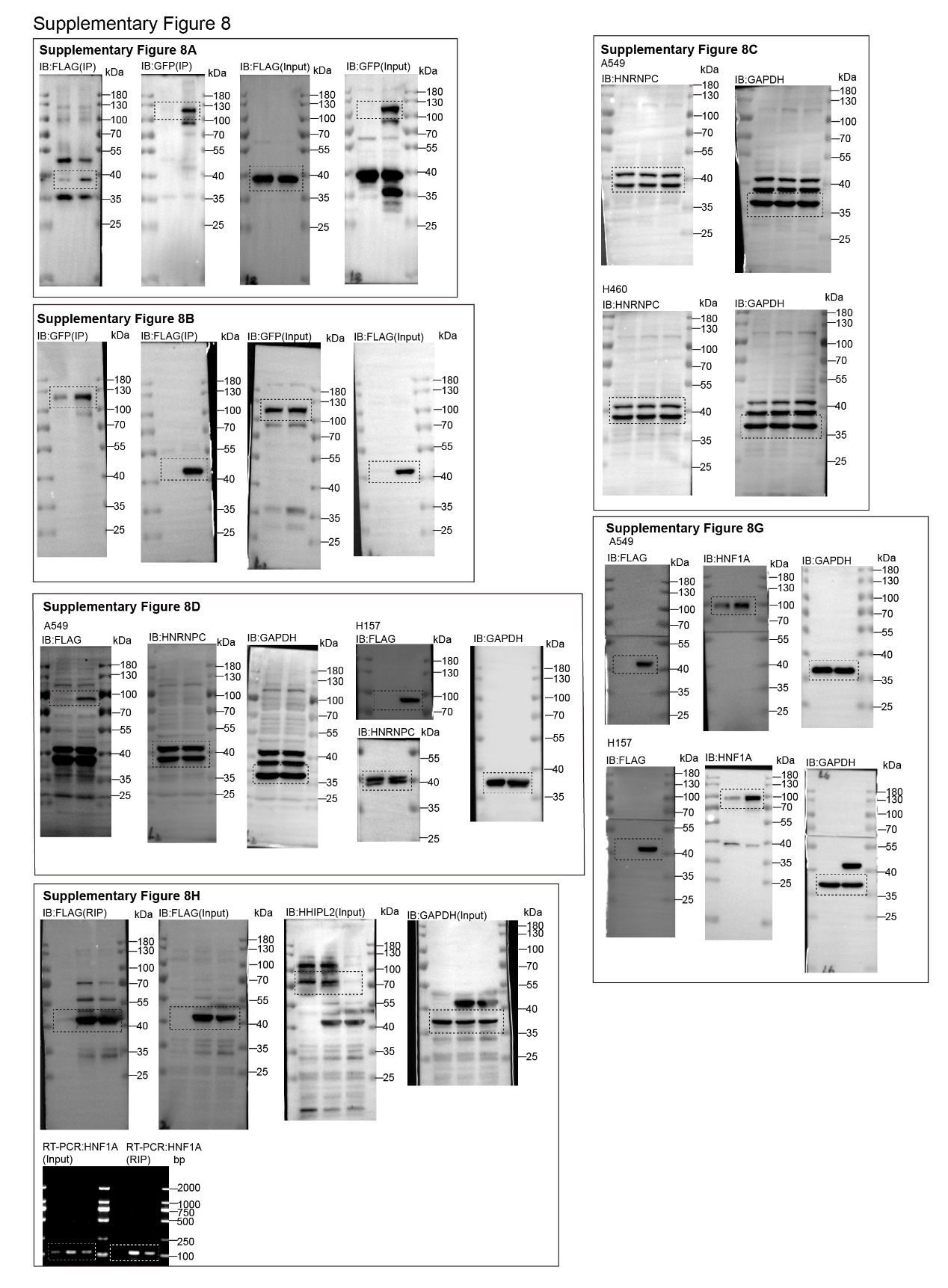


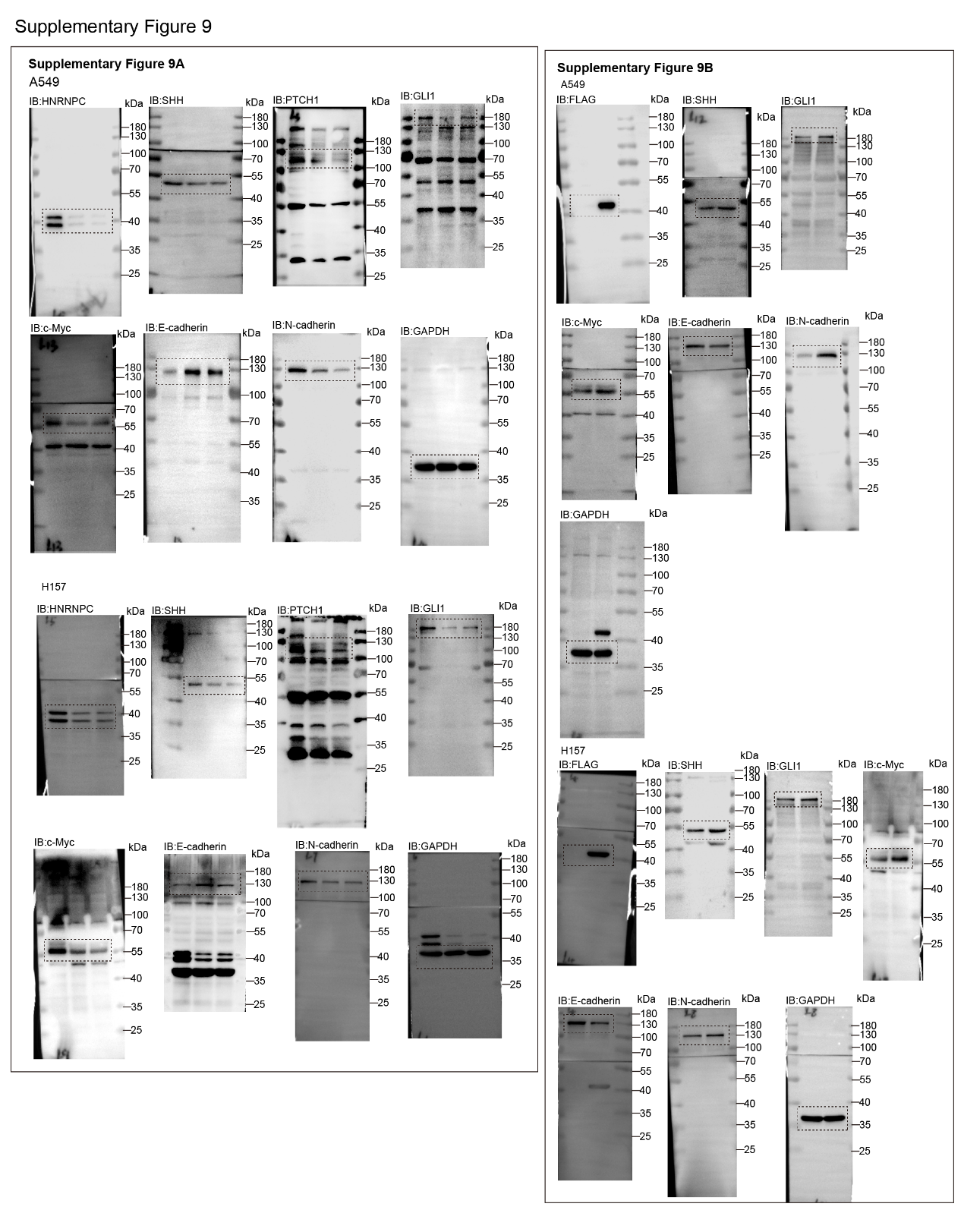

Supplement: Supplementary file 1 — Original Western Blots [file 41419_2025_8331_MOESM1_ESM.docx]
