# Supplementary material for: HHIPL2 positively governs Hedgehog signaling to accelerate non-small cell lung cancer progression via enhancing HNRNPC-mediated HNF1A mRNA stabilization
Source: Cell Death Dis. 2025 Dec 18;17(1):103. doi: 10.1038/s41419-025-08331-3 (PMC12848035; doi:10.1038/s41419-025-08331-3)
Supplement: Supplementary file 2 — Statistical Analysis of the Western Blot Bands [file 41419_2025_8331_MOESM2_ESM.docx]

**Statistical analysis of the Western blot bands**


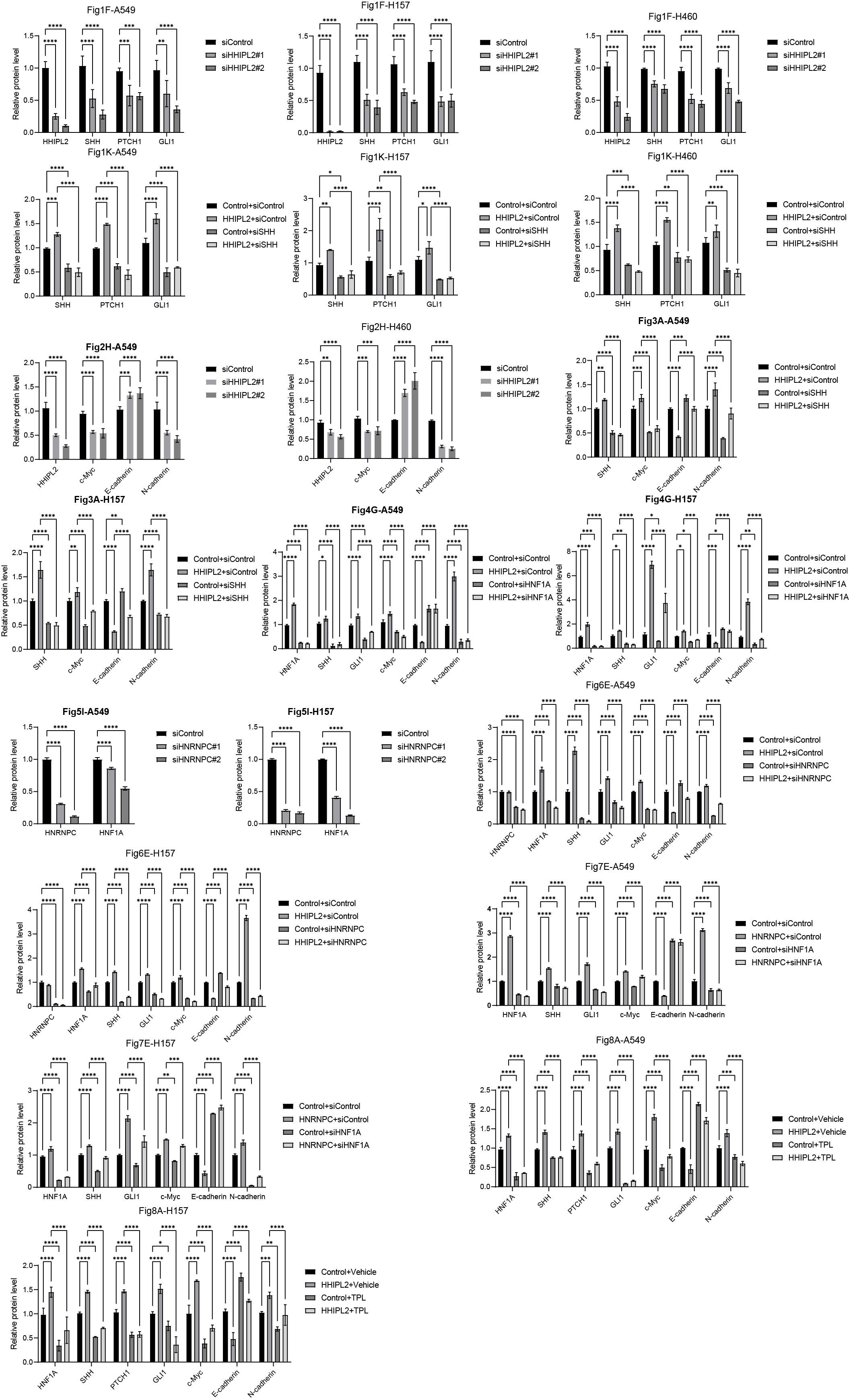


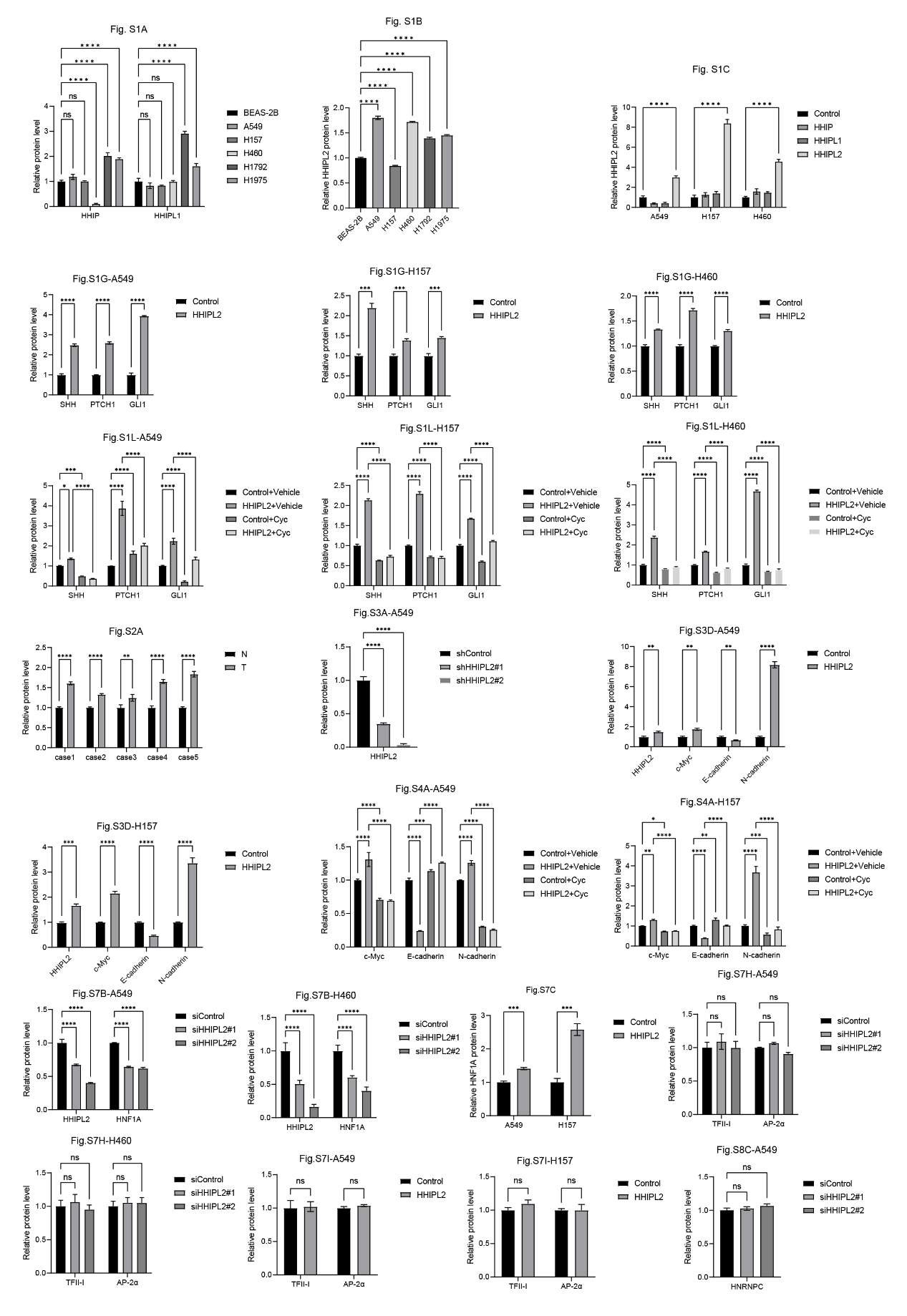

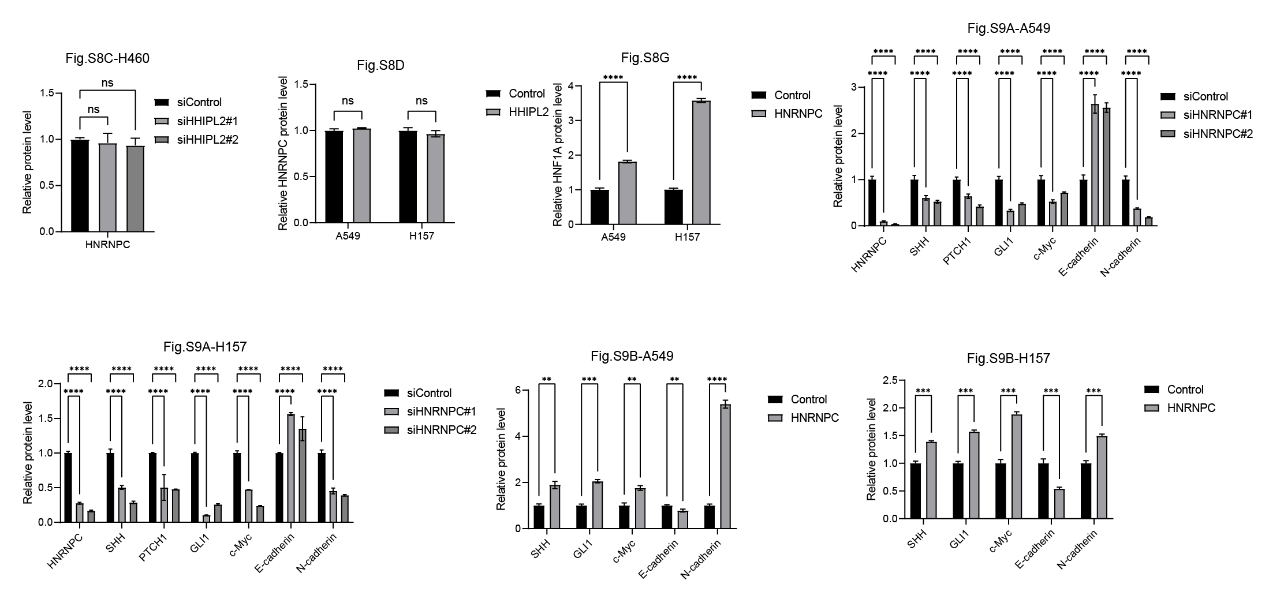


The signal intensity of each Western blot band was measured and standardised to the GAPDH expression level with ImageJ, and statistical data were analyzed using GraphPad Prism. Data are presented as the mean ± SD. Statistical significance was assessed by a one-way ANOVA or a two-sided Student’s *t*-test. **P* < 0.05, ***P* < 0.01, ****P* < 0.001, *****P* < 0.0001, ns, no significance.
