# Supplementary material for: HHIPL2 positively governs Hedgehog signaling to accelerate non-small cell lung cancer progression via enhancing HNRNPC-mediated HNF1A mRNA stabilization
Source: Cell Death Dis. 2025 Dec 18;17(1):103. doi: 10.1038/s41419-025-08331-3 (PMC12848035; doi:10.1038/s41419-025-08331-3)
Supplement: Supplementary file 3 — Supplementary Figure 1–10 and Table 2–4 [file 41419_2025_8331_MOESM3_ESM.docx]

**Supplementary information**

**HHIPL2 positively governs Hedgehog signaling to accelerate non-small cell lung cancer progression via enhancing HNRNPC-mediated HNF1A mRNA stabilization**

Ning Mu, Fanrong Liu, Xiangqing Song, et al

Correspondence

Xiaogang Zhao, PhD. and Peichao Li, PhD., The Second Hospital, Shandong University, Jinan, 250012, P. R. China. E-mail: [zhaoxiaogang@sdu.edu.cn](mailto:zhaoxiaogang@sdu.edu.cn) and [lipeichao@email.sdu.edu.cn](mailto:lipeichao@email.sdu.edu.cn)

**Supplementary information included in this file:**

Supplementary Figures 1 to 10

Supplementary Tables 2 to 4

**Additional Supplementary information:**

Supplementary Table 1. Clinicopathological correlation of HHIPL2, SHH, and HNF1A expression in NSCLC.

Original Western blots.

Statistical analysis of the Western blot bands.


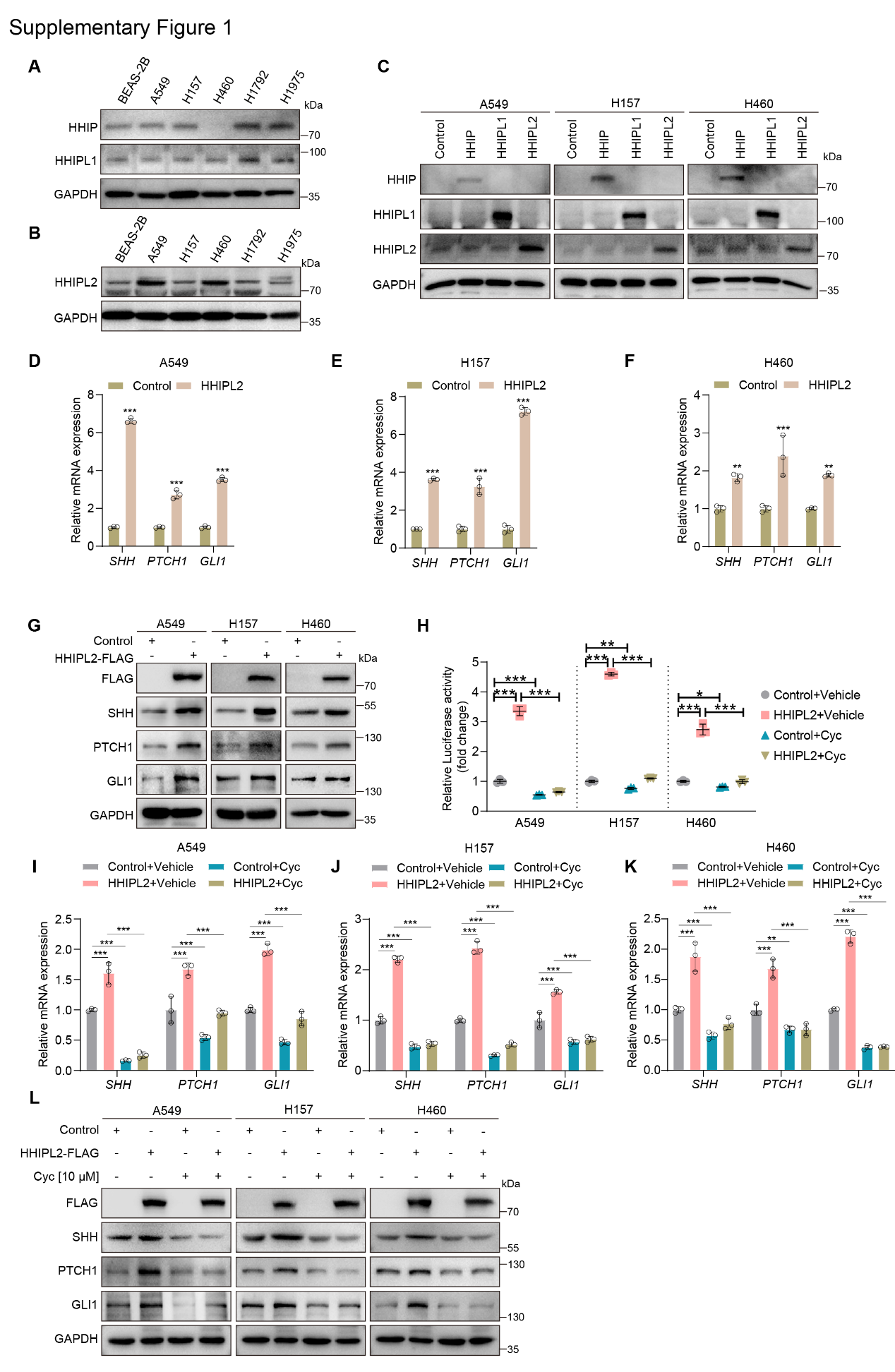


**Supplementary Figure 1. HHIPL2 expression positively correlates with Hedgehog signaling activity, related to Figure 1.**

**A, B.** Relative HHIP, HHIPL1, and HHIPL2 protein levels in five NSCLC cell lines (A549, H157, H460, H1792, H1975) and BEAS-2B cells were determined using western blotting. **C.** Overexpression of HHIP, HHIPL1, and HHIPL2 in A549, H157, and H460 cells. Cell lysates were analyzed by western blotting. **D-F.** Relative RT-qPCR analysis of the mRNA levels of the genes related to the Hedgehog signaling in A549 (**D**), H157 (**E**), and H460 (**F**) cells with or without HHIPL2 overexpression (*n* = 3 in each group). **G.** Overexpression of HHIPL2 in A549, H157, and H460 cells. Cell lysates were analyzed by western blotting. **H.** GLI luciferase assays of A549, H157, and H460 cells overexpressing HHIPL2 with or without Cyc (10 μM) treatment for 24 h. The GLI luciferase activity is normalized to Renilla (*n* = 3 in each group). **I-K.** Relative RT-qPCR analysis of the mRNA levels of the genes related to the Hedgehog signaling in A549 (**I**), H157 (**J**), and H460 (**K**) cells overexpressing HHIPL2 with or without Cyc (10 μM) treatment for 24 h (*n* = 3 in each group). **L.** Overexpression of HHIPL2 in A549, H157, and H460 cells with or without Cyc (10 μM) treatment for 24 h. Cell lysates were analyzed by Western blot. Data in (**D-F, H-K)** are presented as the mean ± SD. Statistical significance was assessed by a two-sided Student’s *t*-test (**D-F**) and a one-way ANOVA (**H-K**). **P* < 0.05, ***P* < 0.01, ****P* < 0.001. Experiments (**A-L**) were repeated at least three times.


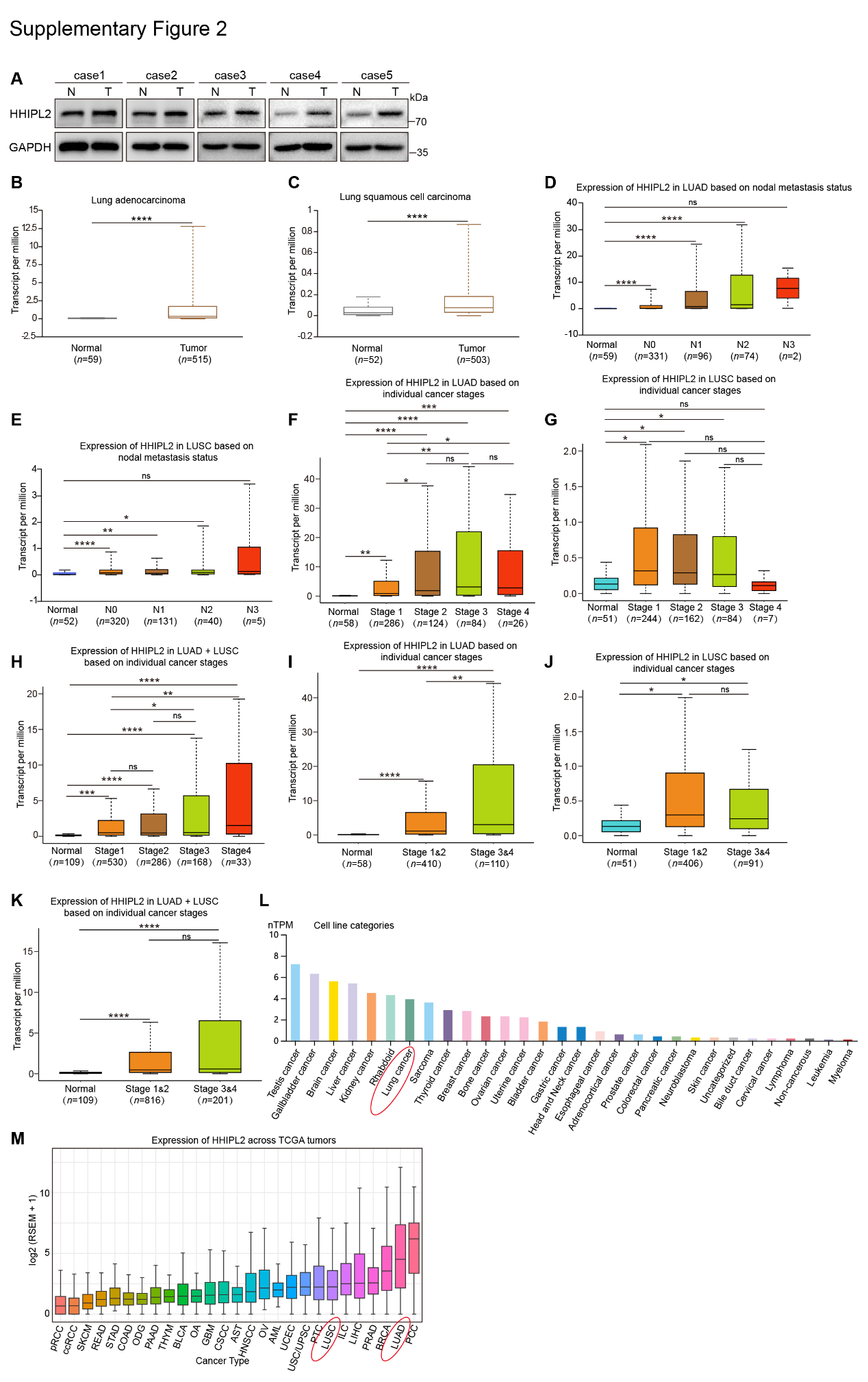


**Supplementary Figure 2. HHIPL2 is aberrantly elevated in NSCLC, related to Figure 2.**

**A.** Western blotting to measure the protein levels of HHIPL2 in 5 pairs of NSCLC tissues and adjacent normal tissues. **B, C.** The HHIPL2 mRNA expression of normal or LUAD (**B**) and LUSC (**C**) tissues from the UALCAN-TCGA online database. **D, E.** The HHIPL2 mRNA expression in LUAD (**D**) and LUSC (**E**) on nodal metastasis status from the UALCAN-TCGA online database. **F-H.** The HHIPL2 mRNA expression in LUAD (**F**), LUSC (**G**), and LUAD + LUSC (**H**) on individual cancer stages from the UCSC Xena-TCGA online database. **I-K.** The HHIPL2 mRNA expression in LUAD (**I**), LUSC (**J**), and LUAD + LUSC (**K**) on cancer stages (I-II, III-IV) from the UCSC Xena-TCGA online database. **L.** The HHIPL2 mRNA expression in human cell lines from the Human Protein Atlas online database. **M.** The HHIPL2 mRNA expression in human tumors from the cBioPortal database. Data in (**B-K)** are presented as the mean ± SD. Statistical significance was assessed by a two-sided Student’s *t*-test. **P* < 0.05, ***P* < 0.01, ****P* < 0.001, *****P* < 0.0001, ns, no significance. Experiments (**A**) were repeated at least three times.


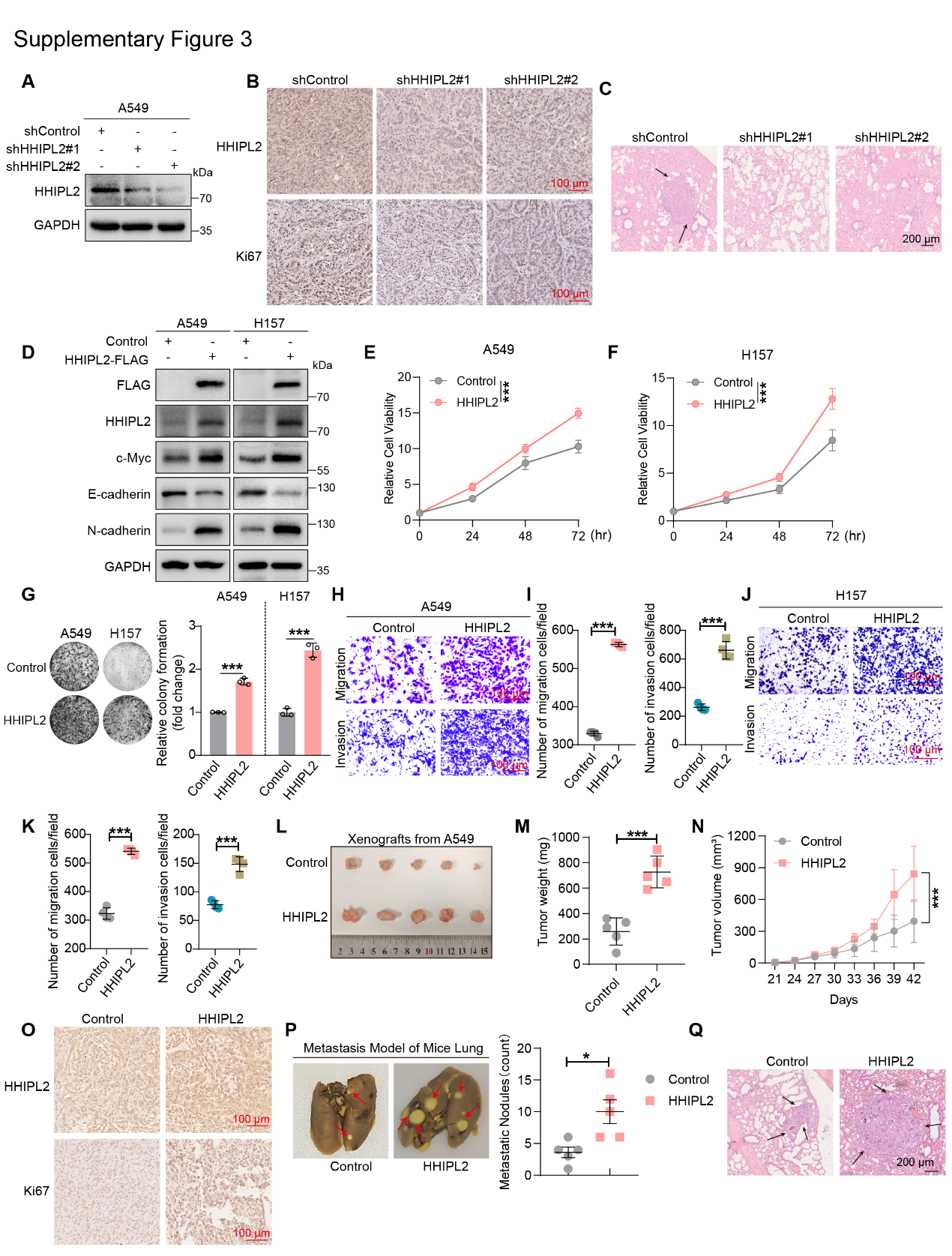


**Supplementary Figure 3. HHIPL2 promotes the proliferation and metastasis of NSCLC cells *in vitro* and *in vivo*, related to Figure 2.**

**A.** Relative HHIPL2 protein levels in A549 cells with HHIPL2 stable knockdown were determined using western blotting. **B.** Representative IHC staining for HHIPL2 and Ki67 in tumor tissue sections with or without HHIPL2 knockdown. **C.** Representative H&E staining in lung sections with or without HHIPL2 knockdown. **D.** Overexpression of HHIPL2 in A549 and H157 cells. Cell lysates were analyzed by western blotting with the indicated antibodies. **E, F.** CCK-8 assays in A549 (**E**) and H157 (**F**) cells with or without HHIPL2 overexpression (*n* = 3 in each group). **G.** Colony formation assays in A549 and H157 cells with or without HHIPL2 overexpression. ImageJ was used to perform quantitative analysis (*n* = 3 in each group). **H-K.** Effects of HHIPL2 overexpression on migration and invasion in A549 cells (**H**, **I**) and H157 cells (**J, K**) using transwell assays. ImageJ was used to perform quantitative analysis (*n* = 4 in each group). Scale bar, 100 μm. **L-N.** Representative images of xenograft tumors after subcutaneous injection of A549 cells with HHIPL2 overexpression and controls (**L**), Tumor weights (**M**), and tumor volumes (**N**) were measured (*n* = 5 per group). **O.** Representative IHC staining for HHIPL2 and Ki67 in tumor tissue sections with or without HHIPL2 overexpression. **P.** Representative images of lung metastasis models in nude mice after tail injection of A549 cells with HHIPL2 overexpression and quantification of pulmonary metastatic nodules (*n* = 5 per group). **Q.** Representative H&E staining in lung sections with or without HHIPL2 overexpression. Data in (**E-G, I, K, M, N, P)** are presented as the mean ± SD. Statistical significance was assessed by a two-way ANOVA (**E, F, N**) and a two-sided Student’s *t*-test (**G, I, K, M, P**), **P* < 0.05, ***P* < 0.01, ****P* < 0.001. Experiments (**A-K, O, Q**) were repeated at least three times.


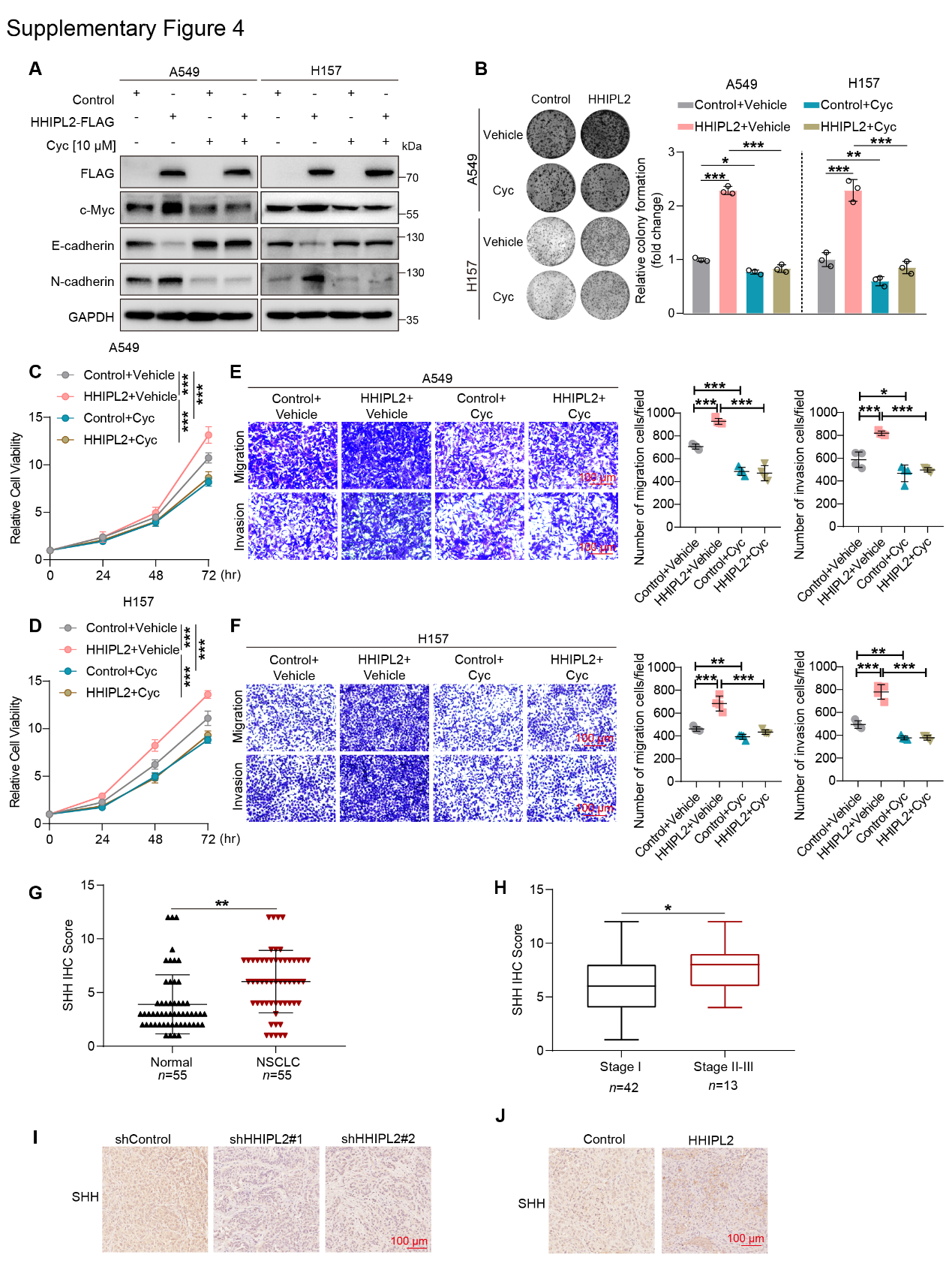


**Supplementary Figure 4. HHIPL2 accelerates NSCLC progression by positively regulating Sonic Hedgehog signaling, related to Figure 3.**

**A.** Overexpression of HHIPL2 in A549 and H157 cells in the presence or absence of Cyc (10 μM) for 24 h. Cell lysates were analyzed by western blotting. **B.** Colony formation assays of A549 and H157 cells overexpressing HHIPL2 after treatment with DMSO and Cyc (10 μM). ImageJ was used to perform quantitative analysis (*n* = 3 in each group). **C, D.** CCK-8 assays of A549 (**C**) and H157 cells (**D**) overexpressing HHIPL2 after treatment with DMSO and Cyc (10 μM) for 0, 24, 48, 72 h (*n* = 3 in each group). **E, F.** Effects of HHIPL2 overexpression in the presence or absence of Cyc (10 μM) on migration and invasion in A549 (**E**) and H157 cells (**F**) using transwell assays. ImageJ was used to perform quantitative analysis (*n* = 4 in each group). Scale bar, 100 μm. **G.** A comparison of SHH IHC scores was performed between NSCLC tumor tissues and normal tissues. **H.** A comparison of SHH IHC scores of NSCLC tissues was performed between Stage I and II-III. **I.** Representative IHC staining for SHH in tumor tissues with or without HHIPL2 knockdown. **J.** Representative IHC staining for SHH in tumor tissues with or without HHIPL2 overexpression. Data in (**B-H)** are presented as the mean ± SD. Statistical significance was assessed by a one-way ANOVA (**B, E, F**), a two-way ANOVA (**C, D**), the Wilcoxon matched-pairs signed rank test (**G**), and the Mann-Whitney test (**H**), **P* < 0.05, ***P* < 0.01, ****P* < 0.001. Experiments (**A-F, I, J**) were repeated at least three times.


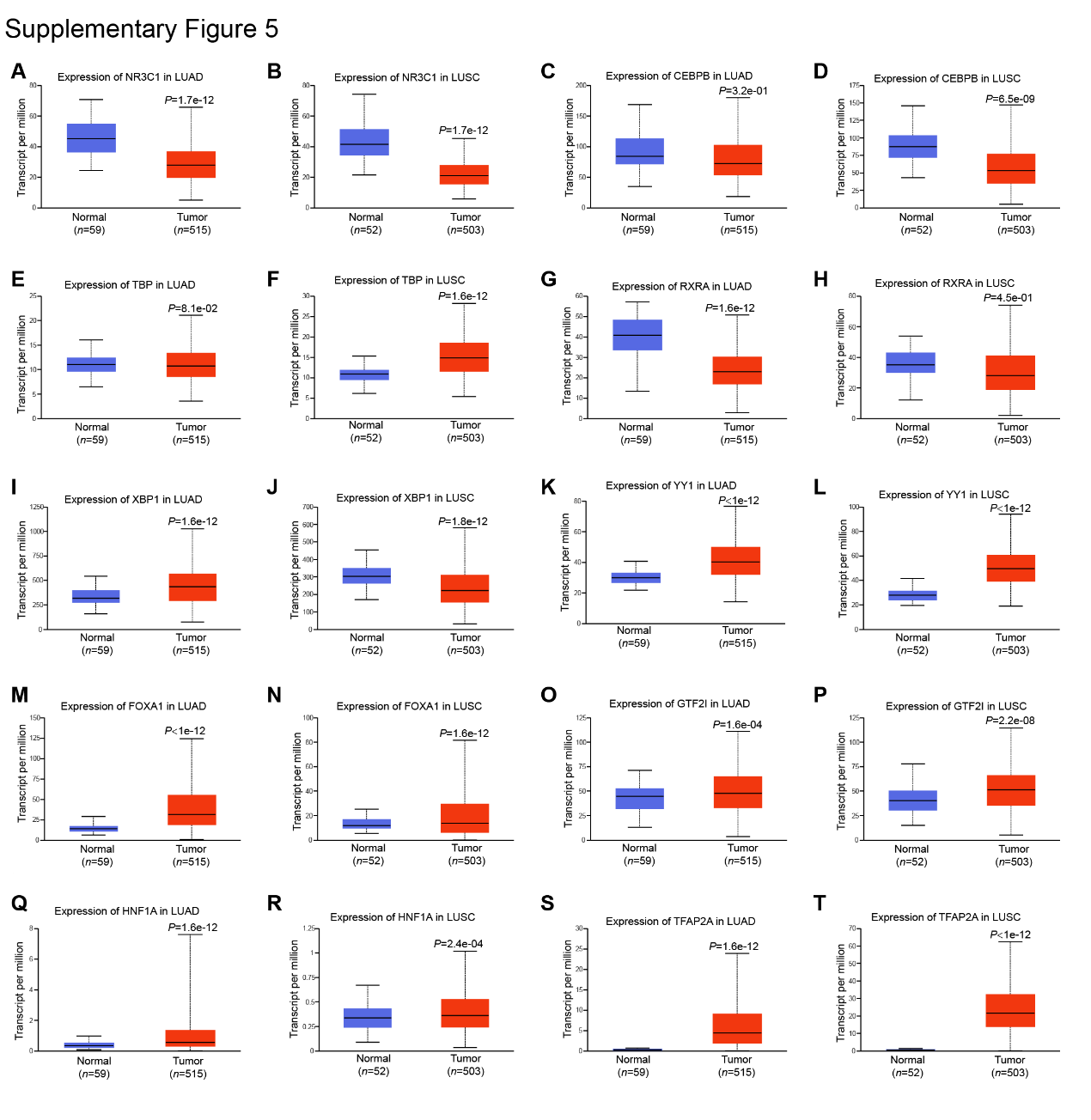


**Supplementary Figure 5. The mRNA expression of possible transcription factors (TFs) binding to the SHH promoter region in normal or LUAD and LUSC tissues, related to Figure 4.**

**A-T.** The mRNA expression of possible 11 transcription factors binding to the SHH promoter region in normal or LUAD and LUSC tissues from the UALCAN-TCGA online database (GR-β and GR, gene: NR3C1; C/EBPβ, gene: CEBPB; TFIID, gene: TBP; RXR-α, gene: RXRA; XBP-1, gene: XBP1; YY1, gene: YY1; HNF3α, gene: FOXA1; TFII-I, gene: GTF2I; HNF1A, gene: HNF1A; AP-2α, gene: TFAP2A).


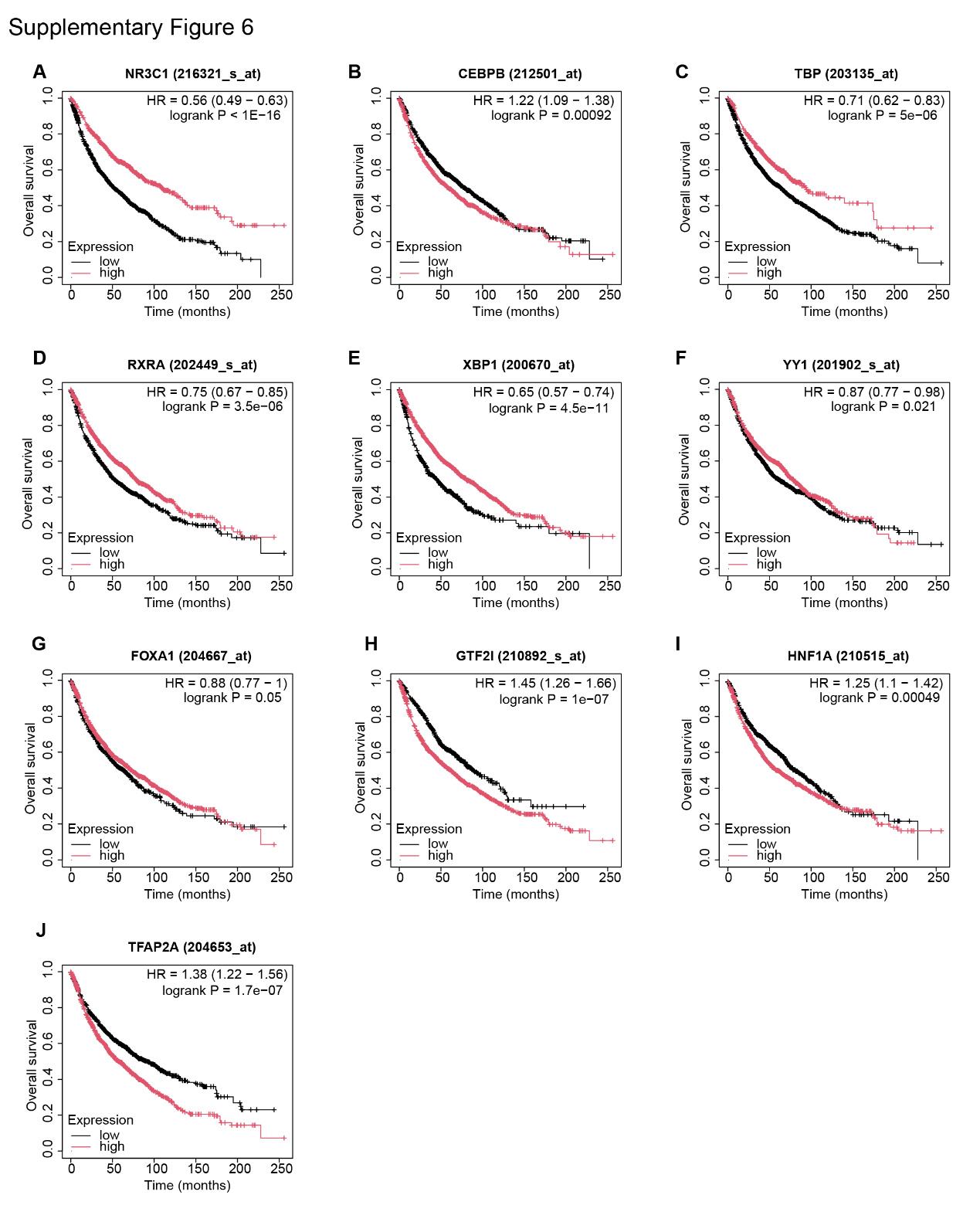


**Supplementary Figure 6. The correlation between mRNA expression of possible transcription factors (TFs) and overall survival in NSCLC, related to Figure 4.**

**A-J.** The correlation between mRNA expression of possible transcription factors (TFs) binding to the SHH promoter region and overall survival in NSCLC from the Kaplan-Meier Plotter online database (GR-β and GR, gene: NR3C1; C/EBPβ, gene: CEBPB; TFIID, gene: TBP; RXR-α, gene: RXRA; XBP-1, gene: XBP1; YY1, gene: YY1; HNF3α, gene: FOXA1; TFII-I, gene: GTF2I; HNF1A, gene: HNF1A; AP-2α, gene: TFAP2A).


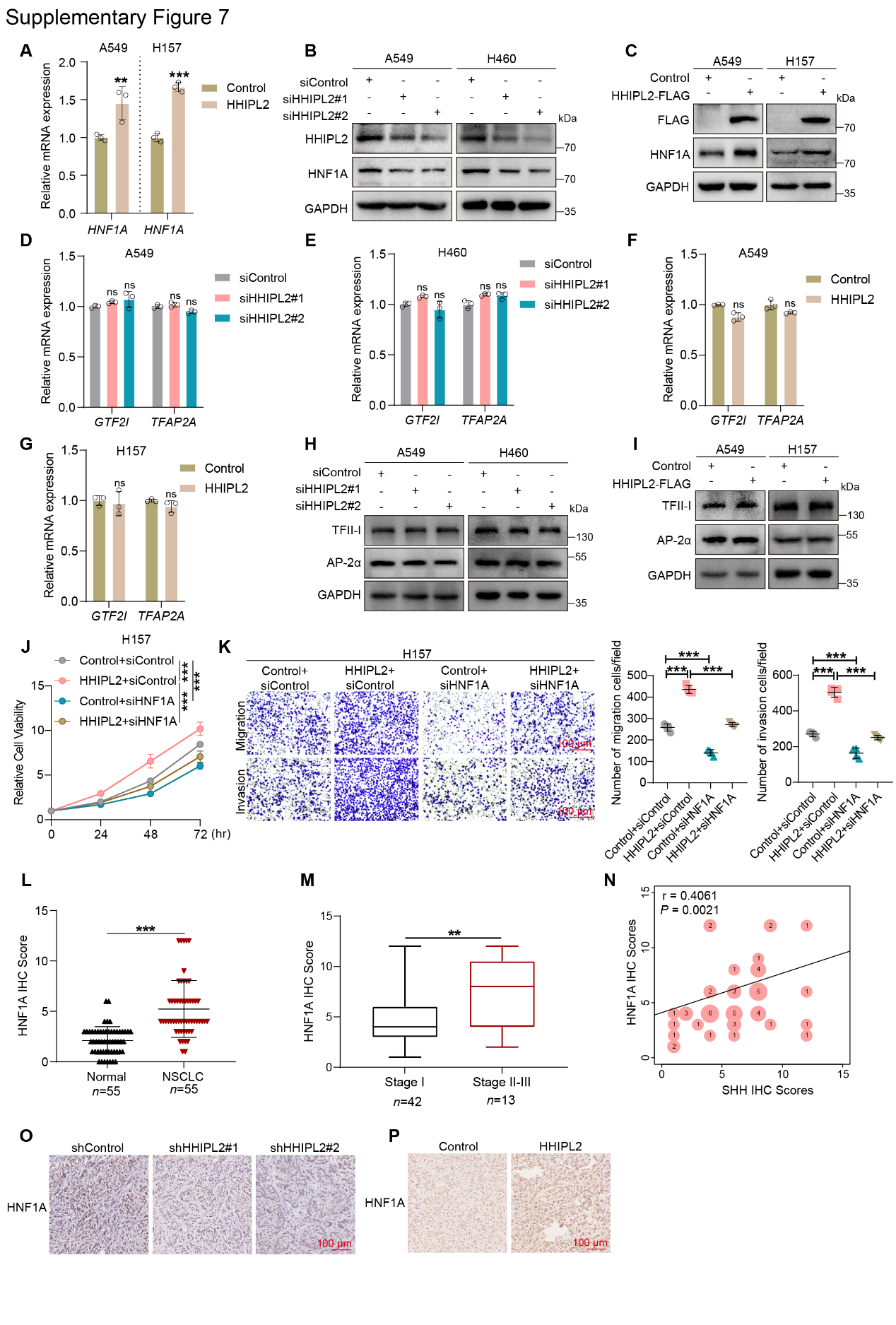


**Supplementary Figure 7.HHIPL2 regulates the Sonic Hedgehog signaling and NSCLC progression through HNF1A, related to Figure 4.**

**A.** Relative RT-qPCR analysis of the HNF1A mRNA levels in A549 and H157 cells with or without HHIPL2 overexpression (*n* = 3 in each group). **B**. Knockdown of HHIPL2 in A549 and H460 cells. Cell lysates were analyzed by western blotting. **C.** Overexpression of HHIPL2 in A549 and H157 cells. Cell lysates were analyzed by western blotting. **D, E.** Relative RT-qPCR analysis of the GTF2I (protein name: TFII-I) and TFAP2A (protein name: AP-2α) mRNA levels in A549 (**D**) and H460 (**E**) cells with or without HHIPL2 knockdown (*n* = 3 in each group). **F, G.** Relative RT-qPCR analysis of the GTF2I and TFAP2A mRNA levels in A549 (**F**) and H157 (**G**) cells with or without HHIPL2 overexpression (*n* = 3 in each group). **H**. Knockdown of HHIPL2 in A549 and H460 cells. Cell lysates were analyzed by western blotting. **I.** Overexpression of HHIPL2 in A549 and H157 cells. Cell lysates were analyzed by western blotting. **J.** Effects of HHIPL2 overexpression with or without HNF1A knockdown in H157 cells using CCK-8 assays (*n* = 3 in each group). **K.** Effects of HHIPL2 overexpression with or without HNF1A knockdown on migration and invasion in H157 cells using transwell assays. ImageJ was used to perform quantitative analysis (*n* = 4 in each group). Scale bar, 100 μm. **L.** A comparison of HNF1A IHC scores was performed between NSCLC tumor tissues and adjacent normal tissues. **M.** A comparison of HNF1A IHC scores of NSCLC tissues was performed between Stage I and II-III. **N.** Statistical correlation analysis between SHH with HNF1A expression in 55 NSCLC tissues. **O.** Representative IHC staining for HNF1A in tumor tissues with or without HHIPL2 knockdown. **P.** Representative IHC staining for HNF1A in tumor tissues with or without HHIPL2 overexpression. Data in (**A, D-G, J-N)** are presented as the mean ± SD. Statistical significance was assessed by a two-sided Student’s *t*-test (**A, F, G**), two-way ANOVA (**J**), a one-way ANOVA (**D, E, K**), the Wilcoxon matched-pairs signed rank test (**L**), the Mann-Whitney test (**M**), and a Spearman's rank correlation coefficient analysis (**N**). ***P* < 0.01, ****P* < 0.001, ns, no significance. Experiments (**A-K, O, P**) were repeated at least three times.

**
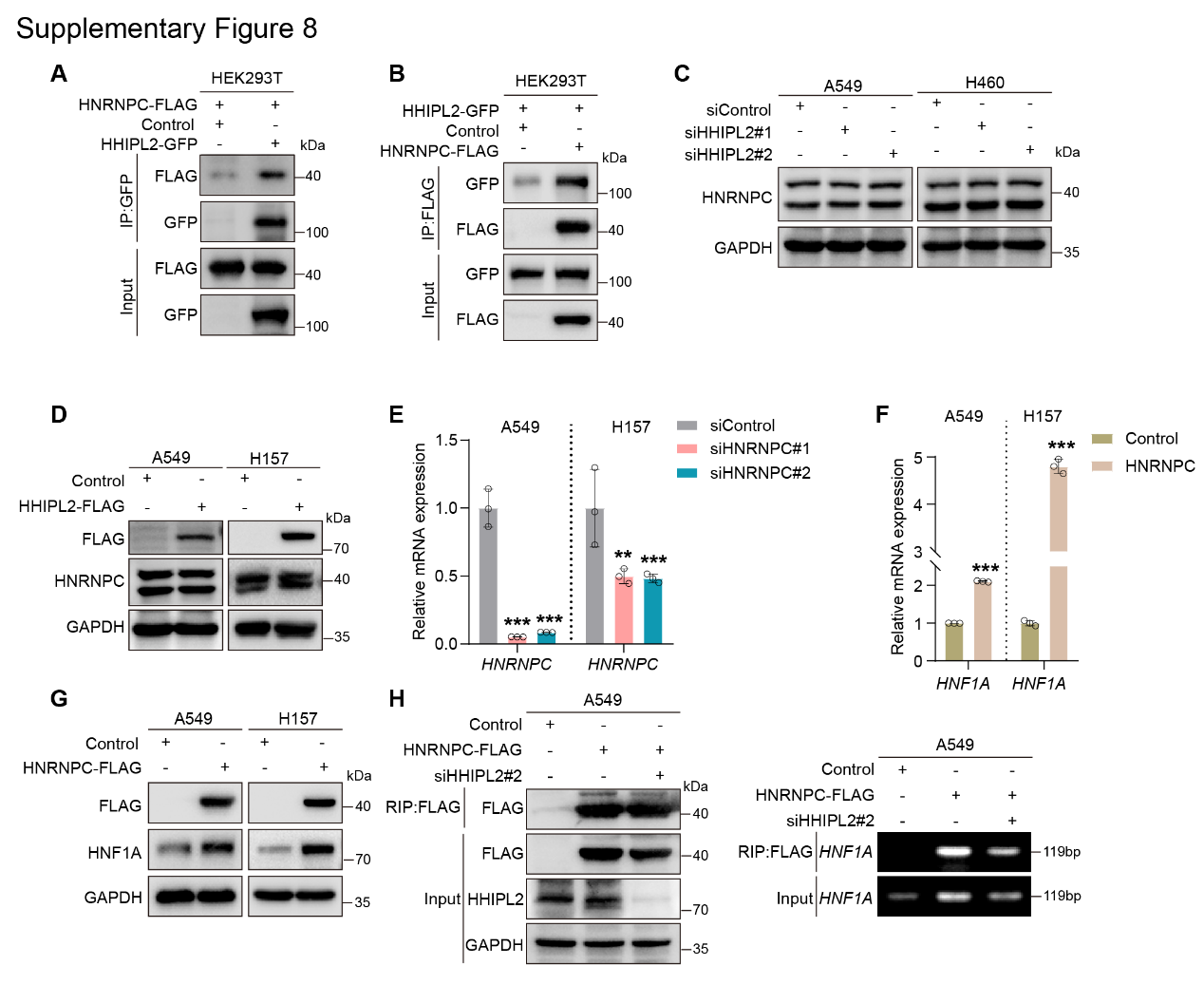
**

**Supplementary Figure 8. HHIPL2 controls the mRNA stability of HNF1A by interacting with HNRNPC, related to Figure 5.**

**A-B**. HEK293T cells were co-transfected with HNRNPC-FLAG and HHIPL2-GFP or control plasmids, and Co-IP assays were carried out with GFP antibody (**A**) or FLAG antibody (**B**), followed by western blotting using the indicated antibodies. **C**. HNRNPC protein levels in A549 cells and H460 cells with HHIPL2 knockdown were determined using the western blot. **D**. HNRNPC protein levels in A549 cells and H157 cells with HHIPL2 overexpression were determined using the western blot. **E**. Relative RT-qPCR analysis of the HNRNPC mRNA levels in A549 and H157 cells with HNRNPC knockdown. **F**. Relative RT-qPCR analysis of the HNF1A mRNA levels in A549 cells and H157 cells with HNRNPC overexpression. **G**. Relative HNF1A protein levels in A549 cells and H157 cells with HNRNPC overexpression were determined using western blotting. **H**. Binding of HNRNPC with HNF1A mRNA was determined by RIP assays with FLAG beads in A549 cells overexpressing HNRNPC-FLAG and knocking down HHIPL2. HNF1A mRNA enrichment was measured by RT-PCR. Data in (**E, F)** are presented as the mean ± SD. Statistical significance was assessed by a one-way ANOVA (**E**) and a two-sided Student’s *t*-test (**F**). ***P* < 0.01, ****P* < 0.001. Experiments (**A-H**) were repeated at least three times.

**
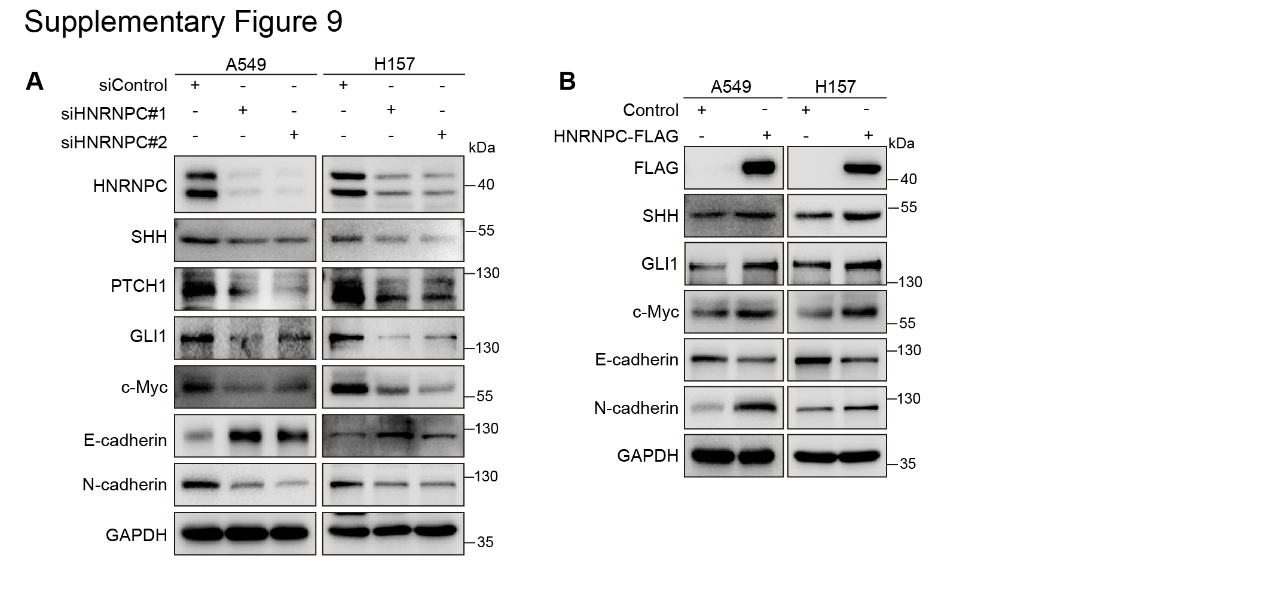
**

**Supplementary Figure 9. HNRNPC positively regulates Sonic Hedgehog signaling and promotes NSCLC progression, related to Figure 6.**

**A.** Knockdown of HNRNPC in A549 cells and H157 cells. Cell lysates were analyzed by western blotting with the indicated antibodies. **B.** Overexpression of HNRNPC in A549 cells and H157 cells. Cell lysates were analyzed by western blotting with the indicated antibodies. Experiments (**A, B**) were repeated at least three times.

**
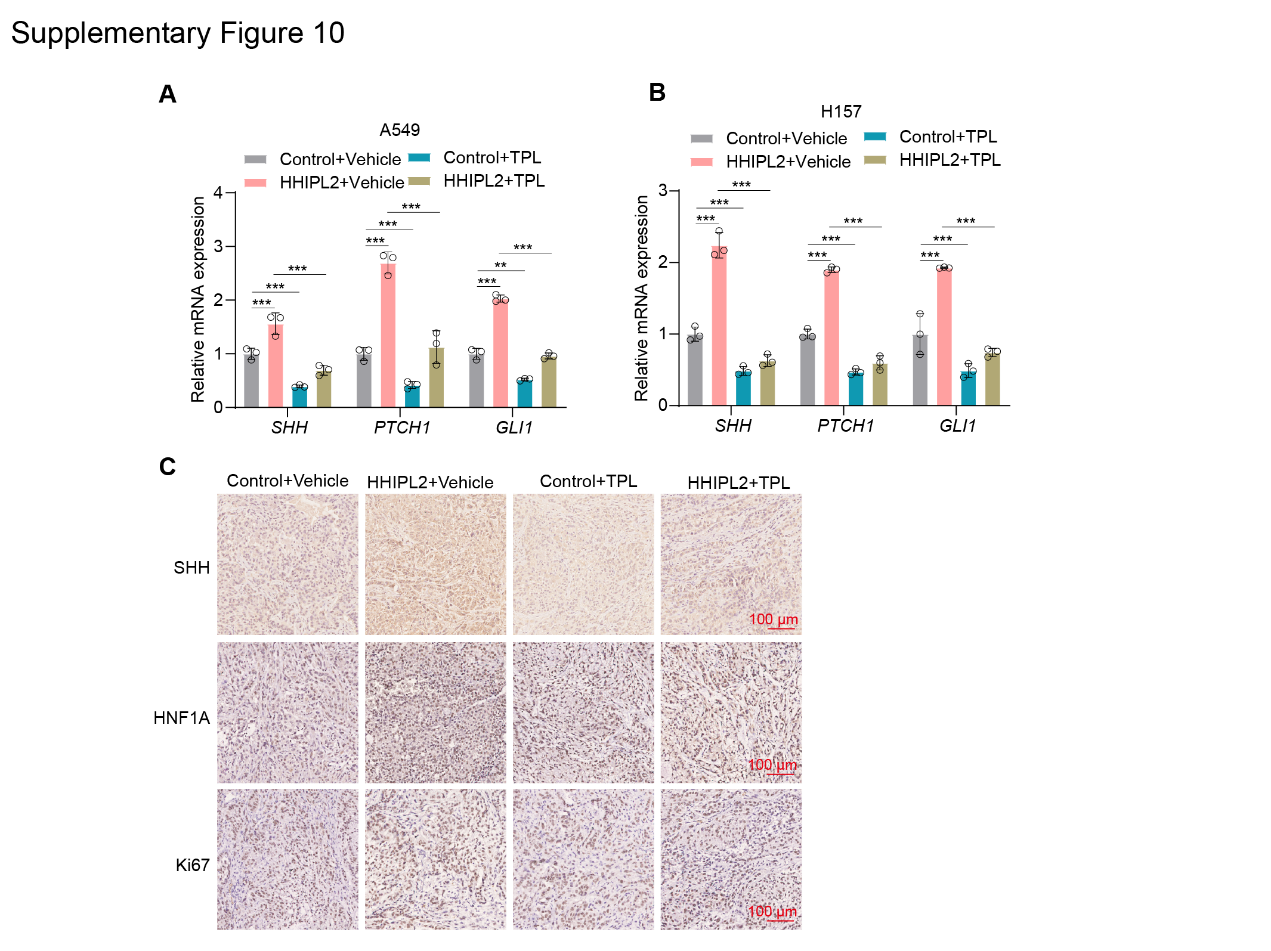
**

**Supplementary Figure 10. Triptolide impedes HHIPL2-mediated proliferation and metastasis via its targeted inhibition of HNF1A expression, related to Figure 8.**

**A, B.** Relative RT-qPCR analysis of the mRNA levels of the genes related to the Hedgehog signaling in A549 (**A**) and H157 (**B**) cells overexpressing HHIPL2 with or without triptolide (TPL, 50 nM) treatment for 24 h (*n* = 3 in each group). **C**. Representative IHC staining for SHH, HNF1A, and Ki67 in tumor tissues. Data in (**A,** **B)** are presented as the mean ± SD. Statistical significance was assessed by a one-way ANOVA. ***P* < 0.01, ****P* < 0.001. Experiments (**A-C**) were repeated at least three times.

**Supplementary Table 2. Antibodies used in the present study**

| **Antibody Name** | **Source** | **No. of Catalogue** | **Application** |
| --- | --- | --- | --- |
| HHIPL2 Antibody | Abclonal | N/A | WB |
| GAPDH Monoclonal antibody | Proteintech | 60004-1-Ig | WB |
| c-Myc Antibody | CST | #9402 | WB |
| E-cadherin Polyclonal antibody | Abcam | Ab40772 | WB |
| N-cadherin Polyclonal antibody | Abcam | Ab76011 | WB |
| Anti-Ki67 | Abcam | ab92742 | IHC |
| Shh Antibody | Abcepta | AP21229a | IHC |
| HHIPL2 Antibody | Abcepta | AP10367c | IHC |
| SHH (C9C5) Rabbit mAb | CST | #2207 | WB |
| Patched Polyclonal Antibody | Abcepta | AP71781 | WB |
| GLI1 Monoclonal antibody | Proteintech | 66905-1-Ig | WB |
| HNF1A Polyclonal antibody | Proteintech | 22426-1-AP | WB, IHC |
| DDDDK-Tag mAb | Abclonal | AE005 | WB, IF |
| DDDDK-Tag Rabbit mAb | Abclonal | AE092 | WB |
| HNRNPC Polyclonal antibody | Proteintech | 11760-1-AP | WB, IP, IF |
| GFP (D5.1) Rabbit mAb | CST | #2956 | WB, IP |
| Lamin B1 Polyclonal antibody | Proteintech | 12987-1-AP | WB |
| Myc-Tag Rabbit mAb | CST | #2278 | WB |
| HHIP Antibody | Sigma | WH0064399M1 | WB |
| HHIPL1 Antibody | Abclonal | N/A | WB |

**Supplementary Table 3. Sequences of shHHIPL2, siHHIPL2, siHNRNPC, siHNF1A**

| **siRNA** | **Sequence (5’to3’)** |
| --- | --- |
| shHHIPL2#1 | Forward: TGCTCTGCCTTCCATTCTAAC |
|  | Reverse: GTTAGAATGGAAGGCAGAGCA |
| shHHIPL2#2 | Forward: CACAATCGCAAGTTCTATATT |
|  | Reverse: AATATAGAACTTGCGATTGTG |
| siHHIPL2#1 | Forward: GCUCUGCCUUCCAUUCUAA (dT)(dT) |
|  | Reverse: UUAGAAUGGAAGGCAGAGC (dT)(dT) |
| siHHIPL2#2 | Forward: CACAAUCGCAAGUUCUAUA (dT)(dT) |
|  | Reverse: UAUAGAACUUGCGAUUGUG (dT)(dT) |
| siHNRNPC#1 | Forward: CAACGGGACUAUUAUGAUA (dT)(dT) |
|  | Reverse: UAUCAUAAUAGUCCCGUUG (dT)(dT) |
| siHNRNPC#2 | Forward: GAAGGAGCUGACCCAGAUA (dT)(dT) |
|  | Reverse: UAUCUGGGUCAGCUCCUUC (dT)(dT) |
| siHNF1A | Forward: GGUCCUACGUUCACCAACA (dT)(dT) |
|  | Reverse: UGUUGGUGAACGUAGGACC (dT)(dT) |
| siSHH | Forward: GGUGUAAGGACAAGUUGAA (dT)(dT) |
|  | Reverse: UUCAACUUGUCCUUACACC (dT)(dT) |

**Supplementary Table 4. Primer sequences for RT-qPCR**

| **Gene** | **Sequence (5’to3’)** |
| --- | --- |
| HHIPL2 | Forward: AAGCAGGATCTTTGCCTGGG |
|  | Reverse: GTGCTCGCCTTGAGGGGTC |
| HNRNPC | Forward: GCAGAGCCAAAAGTGAACCG |
|  | Reverse: ACGTTTCGAGGGCACTACAG |
| HNF1A | Forward: AGACGCTAGTGGAGGAGTGCAA |
|  | Reverse: GGCAAACCAGTTGTAGACACGC |
| SHH | Forward: CCGAGCGATTTAAGGAACTCACC |
|  | Reverse: AGCGTTCAACTTGTCCTTACACC |
| PTCH1 | Forward: GCTGCACTACTTCAGAGACTGG |
|  | Reverse: CACCAGGAGTTTGTAGGCAAGG |
| GLI1 | Forward: AGCCTTCAGCAATGCCAGTGAC |
|  | Reverse: GTCAGGACCATGCACTGTCTTG |
| GTF2I | Forward: GTCAAGAGGCGAACACACAAC |
|  | Reverse: TTGGACGGACAGGATGTATGC |
| TFAP2A | Forward: GACTCGGAGACCTCTCGATCC |
|  | Reverse: GACGGCATTGCTGTTGGAC |
| GAPDH | Forward: GTCTCCTCTGACTTCAACAGCG |
|  | Reverse: ACCACCCTGTTGCTGTAGCCAA |
